# Effects of NMR Spectral Resolution on Protein Structure Calculation

**DOI:** 10.1371/journal.pone.0068567

**Published:** 2013-07-16

**Authors:** Suhas Tikole, Victor Jaravine, Vladislav Yu. Orekhov, Peter Güntert

**Affiliations:** 1 Institute of Biophysical Chemistry, Center for Biomolecular Magnetic Resonance, and Frankfurt Institute of Advanced Studies, Goethe University Frankfurt am Main, Frankfurt am Main, Germany; 2 Swedish NMR Centre, Gothenburg University, Gothenburg, Sweden; 3 Graduate School of Science, Tokyo Metropolitan University, Hachioji, Tokyo, Japan; Spanish National Cancer Center, Spain

## Abstract

Adequate digital resolution and signal sensitivity are two critical factors for protein structure determinations by solution NMR spectroscopy. The prime objective for obtaining high digital resolution is to resolve peak overlap, especially in NOESY spectra with thousands of signals where the signal analysis needs to be performed on a large scale. Achieving maximum digital resolution is usually limited by the practically available measurement time. We developed a method utilizing non-uniform sampling for balancing digital resolution and signal sensitivity, and performed a large-scale analysis of the effect of the digital resolution on the accuracy of the resulting protein structures. Structure calculations were performed as a function of digital resolution for about 400 proteins with molecular sizes ranging between 5 and 33 kDa. The structural accuracy was assessed by atomic coordinate RMSD values from the reference structures of the proteins. In addition, we monitored also the number of assigned NOESY cross peaks, the average signal sensitivity, and the chemical shift spectral overlap. We show that high resolution is equally important for proteins of every molecular size. The chemical shift spectral overlap depends strongly on the corresponding spectral digital resolution. Thus, knowing the extent of overlap can be a predictor of the resulting structural accuracy. Our results show that for every molecular size a minimal digital resolution, corresponding to the natural linewidth, needs to be achieved for obtaining the highest accuracy possible for the given protein size using state-of-the-art automated NOESY assignment and structure calculation methods.

## Introduction

Determining three-dimensional structures of biomolecules experimentally by nuclear magnetic resonance (NMR) spectroscopy has become an invaluable tool for structural and functional studies of proteins at atomic resolution in solution [1]. Well-resolved nuclear Overhauser effect (NOE) signals are critical for obtaining inter-nuclear upper distance limits for three-dimensional (3D) structure calculations [2]. The number and types of experiments necessary for the resonance assignment and structure calculation can vary depending on the complexity and behavior of protein molecules [3]–[5]. In practice, the availability and stability of NMR spectrometers, as well as short lifetimes, molecular stability, and proteolytic degradation of protein molecules limit the total measurement time, and the number of experiments [6]. If the multidimensional experiments are sampled uniformly, there is often not enough time to measure an adequate number of sampled points, which results in poor spectral resolution and ultimately low-quality of the calculated protein structures. It is common practice to use the maximal resolution limited either by the allocated spectrometer time or the sample conditions.

When used in conventional context, NMR spectroscopy often represents a major time bottleneck for sampling adequate number of points for ultimate success of peaks resolution [7], [8]. The digital resolution in the frequency domain refers to the minimum separation between two adjacent points. For a given spectral width, it is proportional to the number of time-domain sampled points. NOESY spectra are notorious for being often crowded with many peaks. A common problem is the presence of many overlapped peaks, with especially severe overlap in the aliphatic area. Consequently, for a successful protein structure calculation it is critical to choose an appropriate digital resolution, aiming to eliminate or minimize peak overlap to an acceptable degree. However, the relation between spectral resolution and the quality of the resulting structures has not been studied thoroughly.

In multi-dimensional NMR, high digital resolution requires many sampled points in the time domain and thus may come at enormous cost of experiment time. The number of measured data points scales up polynomially with the spectrometer field and spectral resolution, and exponentially with the number of dimensions. Furthermore, using long evolution times needed for the high resolution may significantly reduce the sensitivity of the spectrum. Non-uniform sampling (NUS) is known to largely eliminate the above problems [9]–[15]. In combination with appropriate signal processing, NUS allows achieving high digital resolution without blowing up duration of the experiment and compromising sensitivity. Resolving peak overlap is contingent on using high resolution which requires sampling higher numbers of points with longer evolution times. NUS can accommodate longer evolution times even at high magnetic fields and generate an acquisition time grid as fine as needed [16]. It thus becomes the method of choice for resolving peak overlap in NOESY spectra of medium size [17]–[19] and large biomolecules [20]–[26]. However, in some cases of severe overlap or for some specific types of protein folds, this advantage does not result in significant improvement in the structure calculation because a complete separation of peaks in overlapping areas is difficult to achieve. In particular, peak overlap results in resonance assignment ambiguity [27] and may subsequently lead to inaccurate structures. Nonetheless, the resolution of peak overlap in protein NMR spectra can be improved when NUS is utilized instead of uniform sampling. We will present some of the results in this work.

The key feature of non-uniform sampling of NMR signals is that it allows collecting signal data at unequal evolution time intervals. This allows savings in total measurement time relative to the measurement time required for data uniformly sampled on a grid. Each time interval in experimental context is referred to as one data point. Owing to the characteristic signal intensity levels, if a signal is sampled on a decaying profile, an efficient way is that more points should be sampled more when the signal is more intense. This results in a gain in signal sensitivity. The time saving benefits of NUS accrue mainly in indirect evolution dimensions. A series of non-consecutively sampled points in one or more indirect dimensions is called a *sampling schedule*. For instance, an NUS sampling schedule can be prepared to sample *n* out of *N* points of a full linear schedule. Since most NMR signals are sampled while undergoing an exponential decay, sampling schedules often have an exponentially decaying density of samples in indirect dimensions. The maximal possible spectral resolution is defined by the largest evolution time (*t*_max_) of the time-domain sample. This is defined as the last point of the time-domain sampling schedule. Thus, for exponentially decaying signal profiles, the *n* points are mostly spread at the beginning of the evolution time and fewer points are taken at its end so that a higher spectral resolution can be obtained.

Protein NMR structure calculations are now performed predominantly using distance restraints from 3D ^13^C- and ^15^N-resolved NOESY peak lists [28]. Since benefits of improved spectral resolution and signal sensitivity of NUS data accrue mainly in indirect dimensions, and one of the dimensions in 3D NOESY spectra refers to either ^13^C or ^15^N, we chose to study in these spectra the effect of increasing the digital resolution in the ^1^H indirect dimension when NUS was applied simultaneously to both indirect dimensions. In experimental context, since a much wider range of spectral width settings is possible with NUS, the question arises what number of indirect increments is adequate to obtain the highest possible resolution for a protein molecule. The effect of setting the digital resolution has important consequences on the quality of measured signals. In order to study this effect, we study a range of about 20 different digital resolutions. The amount of time for NMR signal measurements for both ^13^C- and ^15^N-resolved NOESY spectra is kept fixed, whereas the level of NUS sparseness and the corresponding digital resolution are allowed to vary. We study computationally a set of about 400 protein molecules obtained from the Protein Data Bank at all digital resolutions, aiming to understand the adequate number of points required for any particular protein molecule to obtain an accurate structure. Moreover, we analyze peak overlap for each protein using its chemical shifts, and study quantitatively how this affect protein structure calculations at all resolutions.

Such a large-scale study would demand huge spectrometer allocation times, if experimental NMR measurements were performed for all proteins and at all digital resolutions. Even for several proteins it would take an unreasonable amount of measurement time, as obtaining a complete experimental set for backbone and side-chain resonance assignment and NOE distance restraint collection for the structure calculation of one protein at one resolution usually takes about one to two weeks. Even with already available chemical shift assignments, the measurement of two 3D NOESY experiments can take several days. Owing to these limitations, the study is unrealistic to be performed experimentally. We thus tried to model everything as realistically as possible: experimentally obtained chemical shift values for ^13^C- and ^15^N-resolved NOESY peak lists were taken from BMRB database, inter-atomic distances were derived from PDB structures, and back-calibrated into peak volumes. All other experimental and NUS sampling parameters such as spectral widths, numbers of time-domain points, and spectrometer carrier positions were taken equal to those used for previously performed experiments for medium sized protein molecules [29], [30]. More detailed description of the experimental parameters used in modeling the spectral resolution is given in the *Materials and Methods* section and in Table S1. We perform automated NOESY cross peak assignment and structure calculations using distance restraints from the modeled NOESY peak lists [28] and dihedral angle ranges obtained from backbone chemical shifts. Consequently, we provide qualitative results on how the digital resolution affects protein structure calculations. The effects of the digital resolution on the signal-to-noise ratio per unit of measurement time, the total number of peaks, peak overlap, and protein structure calculations were evaluated.

## Theory

### S/N for Uniformly and Non-uniformly Sampled Data

The digital resolution Δ*f* of a spectrum depends on the largest evolution time, which is proportional to the number of data points *N* sampled in the time domain. For uniform sampling, it is given by Δ*f* = 1/(*N*Δ*t*), where Δ*t* is the dwell time between two consecutive sampled points in the time-domain.

The height *S* of a signal in a Fourier-transformed NMR spectrum is proportional to the integral of its corresponding time-domain NMR signal, or for digital sampling on a grid FID it is given over the acquisition time *t*_max_ by the integral
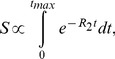
(1)where *R*_2_ is the transverse relaxation rate constant of the resonance signal and *t* corresponds to the evolution time of the sampled data point [31].

For large biomolecules the transverse relaxation rate *R*_2_ is proportional to their molecular rotational correlation time *τ*_c_ [32]–[34]. An estimate of *τ*_c_ for globular proteins may be given as *τ*_c_ ≈ *M*×4×10^−11^, where *M* is the molecular mass in Dalton. Thus the transverse relaxation rate constant of a protein may be written in terms of molecular mass as *R*_2_ = *kM*. For NOESY spectra the proportionality constant *k* = 0.003 Da^−1^s^−1^ can be determined using average curves of transverse relaxation times of H^α^ and H^β^ atoms versus rotational correlation times [33].

In the case of Fourier processing, the integral in equation 1 becomes a sum when data points are measured at discrete time points. It can be expressed in terms of the molecular mass as
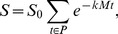
(2)where *P* is the set of all uniformly or non-uniformly sampled points and *S*_0_ the proportionality constant for the signals.

Assuming that for a fixed number of FIDs the amount of noise does not depend on the uniform or non-uniform sampling schedule, the improvement of the signal-to-noise (S/N) ratio for non-uniformly sampled data (S/N)_NUS_ over uniformly sampled data (S/N)_US_ becomes
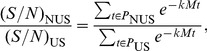
(3)where *P*_NUS_ and *P*_US_ are the sets of non-uniformly and uniformly sampled points in the time domain, respectively. Eq. 3 provides the comparative gain in signal sensitivity for non-uniform vs. uniform sampling schedules obtained as a function of digital resolution.

### Minimal Reasonable Number of Increments for a Given Molecular Weight

Peak resolution is also limited by the natural linewidth of the peaks. For a Lorentzian lineshape, the natural linewidth (full-width at half-maximum) of the peaks is *L* = *R*_2_/π and thus proportional to the overall rotational correlational time *τ*_c_ of the protein and the molecular size of the molecule [32]. The linewidth can be expressed approximately in terms of molecular mass *M* as *L* = *kM*/π. Practically, the spectral resolution is limited by the lifetime of the detected coherence. It should therefore be comparable with the natural linewidth, i.e. 2Δ*f* ≈ *L*. Additionally, one should consider also the unresolved homonuclear couplings and limitations imposed by constant-time evolution. The minimal reasonable number of increments can thus be expressed in terms of the linewidth as *N* ≈ 1/(*L*Δ*t*) ≈ 2π/(*kM*Δ*t*). The proportionality constant *k* can be calculated using the transverse relaxation times of the detected coherences and the molecular rotational correlation times [33]. The dwell time Δ*t* between two consecutive sampled points is dependent on the corresponding spectral width.

## Materials and Methods

### Experimental Parameters for Calculating Spectral Resolution

The spectral resolutions for ^13^C- and ^15^N-resolved NOESY peak lists were modeled based on the experimental parameters such as spectral widths, spectrometer carrier positions, and the numbers of sampled points in the direct, and ^1^H and ^13^C or ^15^N indirect dimensions. The parameters were chosen from the already performed NUS experiments for the protein RcsD-ABL-HPt (23 kDa) and two fused proteins, namely, Ub2_NBR1-LIR (12.4 kDa), and Ub2_p62-LIR (12 kDa) [29], [30]. The prime objective of using parameters equal to the previously performed experiments was to model the spectral resolution corresponding to the measured numbers of points in a typical spectral width setting. Additionally, the same set of parameters was used to generate NUS schedules (described below) for both the indirect dimensions in each NOESY spectra. In order to achieve similar digital resolutions in both ^13^C- and ^15^N-resolved NOESY spectra, the same set of numbers of points being studied was used in ^1^H indirect dimensions. Assuming an inter-scan delay (D1) of one second, the total measurement time for the given number of FIDs was assumed to be 48 hours. The entire set of parameters is listed in Table S1.

### Protein Data Bank NMR Structures Data Set

A set of experimentally determined and uniformly referenced chemical shifts for 400 protein structures was obtained from RefDB [35]. Protein structures were chosen so that the molecular sizes are above 10 kDa and the chemical shifts data are available for five or more types of backbone atoms (C’, C^α^, C^β^, H^N^, H^α^, N). The experimentally determined chemical shift values had originally been deposited in the Biological Magnetic Resonance Data Bank (BMRB) [36]. The corresponding three-dimensional protein structures were obtained from the Protein Data Bank (PDB) [37]. The optimal amino acid residue ranges for the PDB structures were obtained using the CYRANGE program [38] for maintaining consistency in reporting RMSD values of calculated atomic coordinates. From the 400 initially selected protein structures, nineteen proteins structures, for which optimal residue ranges cannot be computed with CYRANGE because only one conformer is available in the PDB, were removed from the analysis.

### Spectral Digital Resolution as a Function of the Number of Sampled Points

If *N* points are uniformly sampled in the time-domain then after Fourier transformation *N* points are used to represent the signal in frequency domain. The minimum separation between two adjacent data points in the frequency domain defines the digital resolution in an NMR spectrum. In other words, the resolution is determined by the number of points sampled in the time-domain signal. Changing the digital resolution in frequency domain is equivalent to changing the number of sampled points in the corresponding time-domain. Since we study the effects of high digital resolution on peak overlap and protein structure calculations, we refer to the observed changes interchangeably as a function of the digital resolution of the number of sampled points.

### Sampling Schedules and S/N Calculation

In order to study the effect of the digital resolution on protein structure calculations, we varied the digital resolution for the ^1^H indirect dimension by changing the maximal number of points from 28 to 1250 (Table S1). The numbers of sampled points for the carbon and nitrogen dimensions of ^13^C- and ^15^N-resolved NOESY spectra were set to 64 complex points each. Uniformly sampled schedules, containing all linear points, were generated for comparison calculations. Non-uniformly sampled schedules were prepared using *nussampler* [39]. In the context of NUS, a fixed total measurement time refers to measuring a fixed number of FIDs for each NOESY spectrum. The current version allows generating schedules with options for incremental matched sampling with and without repetitions of sampled points, examples are given in Figure S2. For all calculations in this paper repetitions of sampled points were allowed. The signal-to-noise improvement ratios were calculated using equation 3 with uniformly and non-uniformly sampled schedules.

### Peak Lists Preparation

The chemical shift table for each protein was obtained from experimentally determined chemical shifts values deposited in RefDB [35]. The ^13^C- and ^15^N-resolved NOESY peak lists for all structures in the dataset were back-calculated with CYANA [40]–[43] using the chemical shift tables and a calibration of inter-nuclear spatial distances in the range between 2.5 and 4.5 Å into peak volumes. The peak lists were refined based on two different criteria. First, the peaks below a certain signal intensity threshold level (2.5 times the noise level) were removed from the lists. Secondly, remaining peaks were removed if they overlapped with any other peak in all dimensions (i.e. closer than the digital resolutions or the estimated linewidths, whichever is higher). This means that after removal of any overlapped peaks, the peak lists essentially had no peak overlap. The size of the peak lists is the direct consequence of the amount of peak overlap present in the corresponding NOESY spectra at a given digital resolution. The peak lists were produced separately for all structures in the dataset and for all digital resolutions.

### Chemical Shift Spectral Overlap Index

A chemical shift spectral overlap (CSSO) index was defined as the average number of overlaps present for each peak in a peak list before the above-mentioned removal of overlaps. The overlap in a cross peak was checked using the chemical shifts values in each of the dimensions. Chemical shift difference values between a cross peak and any other cross peaks in all three dimensions were calculated. These differences were compared simultaneously with the digital resolution and the natural linewidth in each of the dimensions. A cross peak was counted as overlapped if the corresponding differences were less than the digital resolution or the natural linewidth, whichever is higher, in all the dimensions. The cross peak was retained if no overlap was observed with any other cross peaks in any one of the dimensions. The index was calculated separately for ^13^C- and ^15^N-resolved NOESY peak lists and studied as a function of digital resolution. We further analyzed changes in CSSO indices by grouping calculated protein structures based on molecular sizes or the “final” RMSD values (obtained at the highest digital resolution by sampling 1250 points).

### CYANA Structure Calculation Protocol

Protein structure calculations with the CYANA software package can be performed using ^13^C- and ^15^N-resolved NOESY peaks and information obtained from backbone chemical shifts. The former yield inter-atomic spatial upper distance limits; the latter provide backbone torsion angle restraints. The inter-atomic upper distance limits were obtained with CYANA using a calibration of ^13^C- and ^15^N-resolved NOESY peaks in the range of spatial distances 2.5–4.5 Å, with the median of the distribution set to 4.0 Å. The *φ* and *ψ* torsion angle restraints were generated with the TALOS+ software [44] on the basis of the backbone and C^β^ chemical shifts. Structure calculations were performed using the standard CYANA protocol that uses all this information [2], [42]. The heavy-atom RMSD values between the atomic coordinates of the resulting protein structures and the corresponding reference structures from the PDB were obtained using the optimal amino acid residue ranges determined by CYRANGE [38].

The method was applied to the 381 protein structures at all digital resolutions. The results were characterized by calculating the mean RMSD value of the distribution. We fitted the histogram of all final RMSD values to a gamma distribution, since all the RMSD values are non-negative. The function *fitdist* from the R software environment for statistical computing (http://www.r-project.org/) package *fitdistrplus* was used for performing the maximum likelihood fitting. Additionally, we analyzed the 381 proteins by dividing them into three different groups by molecular size. The size ranges were 10–15 kDa, 15–20 kDa, and 20–35 kDa with 280, 76, and 25 protein structures, respectively.

## Results

**Figure 1:**
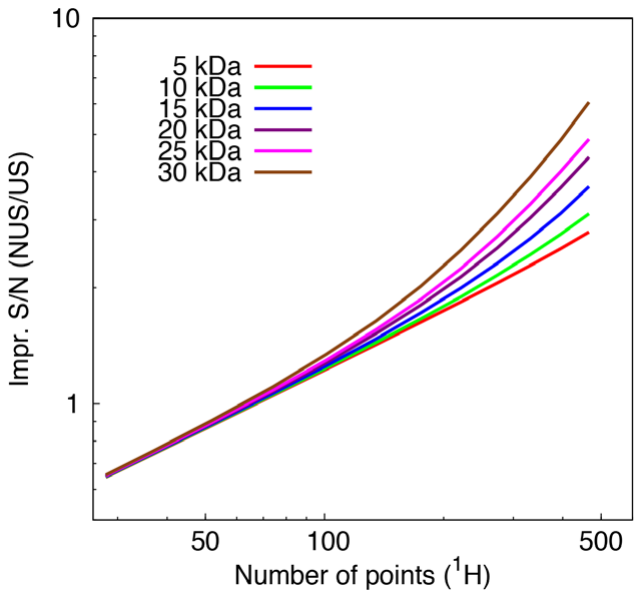
Improvement in signal-to-noise ratio per unit of measurement time by non-uniform sampling. The improvement in S/N ratio in ^13^C-resolved NOESY peaks for non-uniformly over uniformly sampled schedules using the parameters in Table S1 is shown for the six proteins with PDB IDs 2BBX (5 kDa), 1D5G (10 kDa), 1XKE (15 kDa), 1JBJ (20 kDa), 1TTE (25 kDa), and 2JT2 (30 kDa).

Increasing the digital resolution has important consequences for the success of the protein structure calculation protocol. First of all, it is reflected in the RMSD values of the calculated protein structures from the corresponding reference structures. Additionally, we analyzed the average S/N ratio of peaks per unit of measurement time (Figure 1), peak counts for ^13^C- and ^15^N-resolved NOESY peak lists (Figure S1), and chemical shift spectral overlap (CSSO) indices (Table S3).

**Figure 2:**
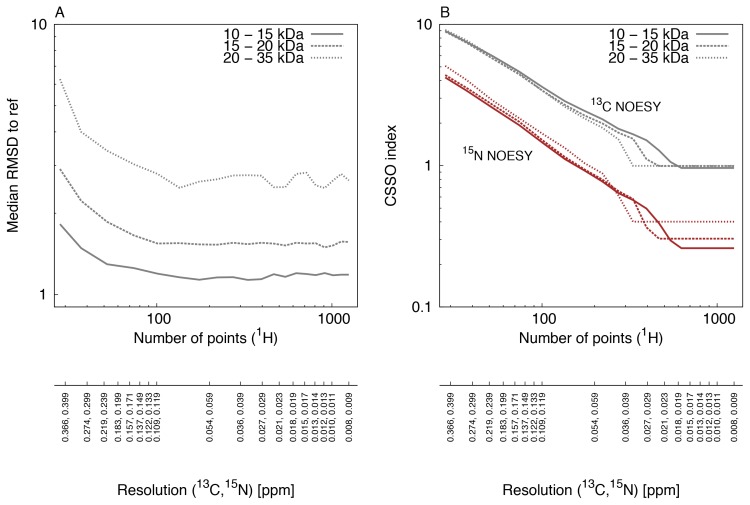
Median RMSDs to reference structures and chemical shift spectral overlap (CSSO) indices for protein structures of different size groups. (A) Median of the heavy atom RMSD to the reference structures are shown for 280 protein structures in the molecular size range from 10 to 15 kDa (solid), 76 protein structures in the molecular size range 15–20 kDa (dashed) and 25 protein structures in the molecular size range 20–35 kDa (dotted). RMSD values were calculated for the residue ranges determined by the CYRANGE algorithm. (B) Average CSSO index for ^13^C-resolved NOESY (black) and ^15^N-resolved NOESY (red) peak lists for the proteins structures of the same size-groups as in A.

**Figure 3:**
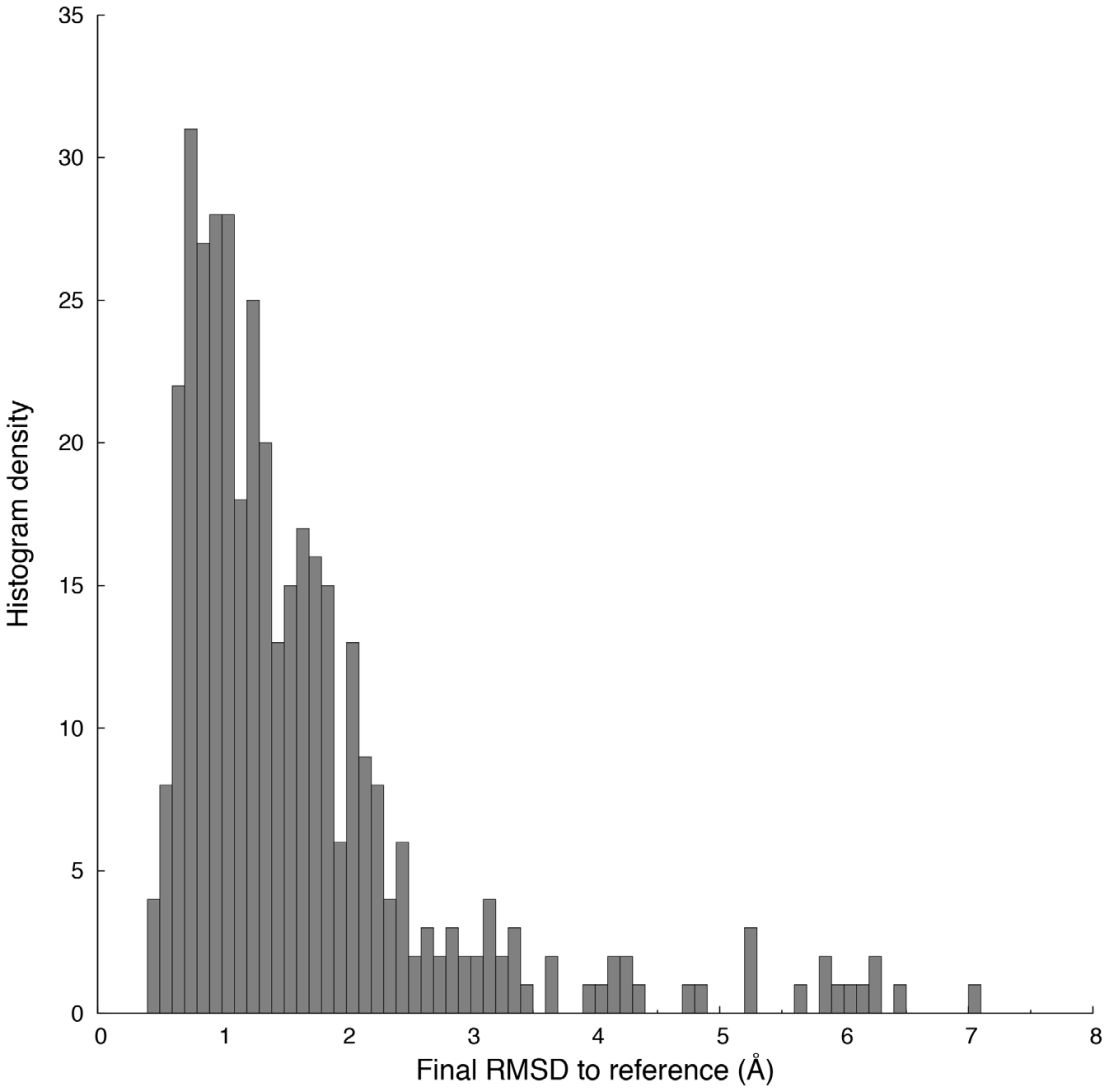
Heavy-atom RMSD values obtained at the highest resolution. The histogram shows the heavy atom RMSD values of 381 protein structures. obtained at the highest digital resolution corresponding to 1250 sampling points.

**Figure 4:**
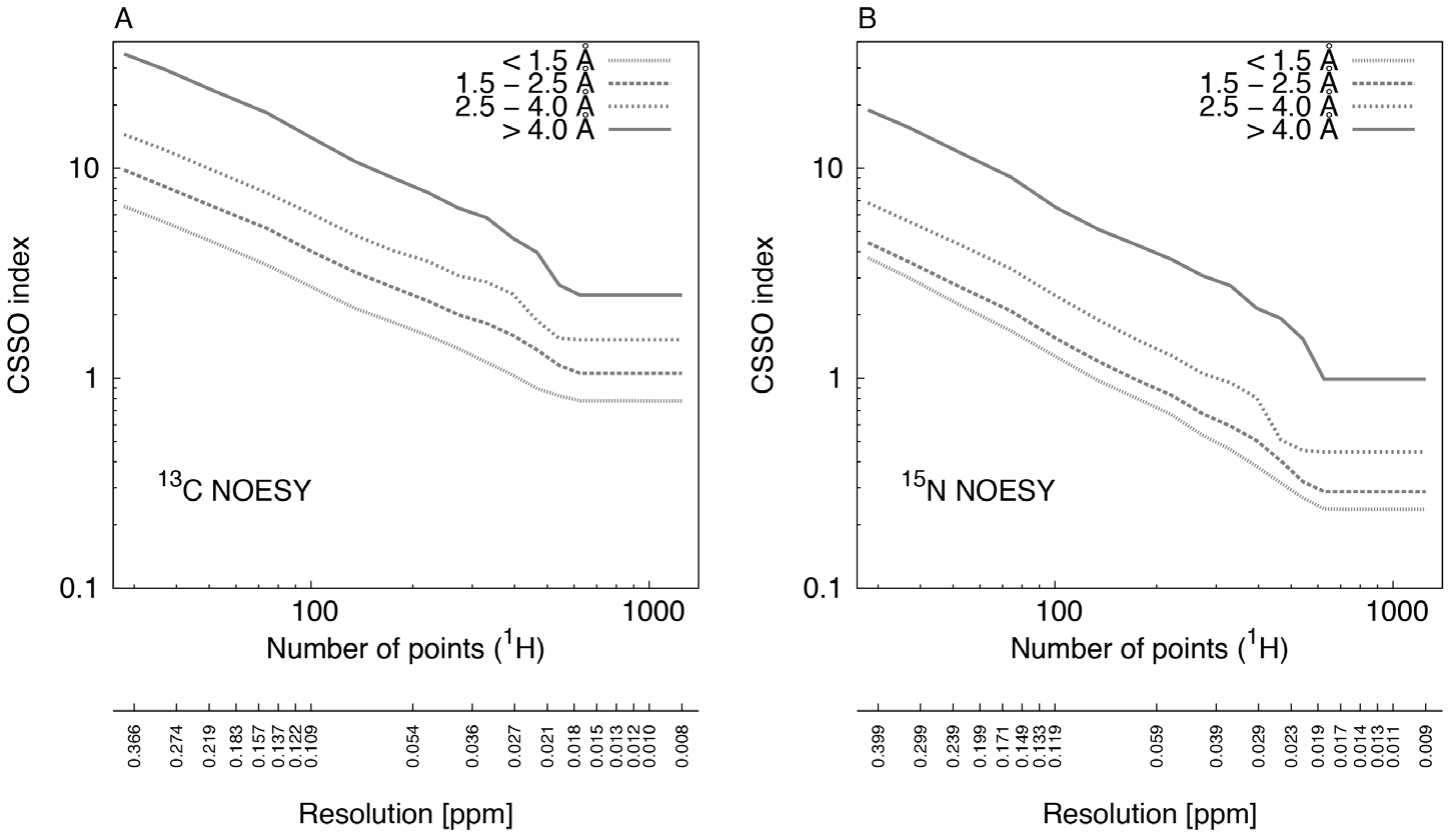
Chemical shift spectral overlap (CSSO) indices as a function of the digital resolution. (A) ^13^C-resolved NOESY peak lists. The fine dotted line shows the average CSSO index for 149 protein structures with heavy atom RMSD to the reference structure below 1.5 Å calculated at the highest digital resolution (obtained by sampling 1250 points). Dashed, dotted, and solid lines correspond to 210, 14, and 4 protein structures with RMSD values of 1.5–2.5, 2.5–4.0, and more than 4.0 Å, respectively. (B) Same data for ^15^N-resolved NOESY peak lists.

We obtained for each of the 381 protein structures the RMSD values as a function of digital resolution, to which we refer here as ‘profiles’. All profiles are shown in Figure S4. These profiles were grouped by molecular size or by RMSD values of calculated structures. The result is shown in Figure 2. The histogram of all RMSD values obtained from the profiles at the highest resolution is shown in Figure 3. The RMSD values and the fitted gamma distribution show a statistically significant fit. For the same data, we computed the CSSO indices, which are shown in Figure 4.

### Improvement in S/N Ratio Per Unit of Measurement Time

To illustrate the effects of digital resolution on S/N ratios, the method was applied to a set of six proteins of varying molecular sizes, increasing in steps of approximately 5 kDa from 5 to 30 kDa. Figure 1 shows the improvement of the S/N ratios per unit of measurement time for ^13^C-resolved NOESY peaks of the six protein structures, due to using NUS instead of uniform sampling as described in the *S/N for non-uniformly sampled data* part of the *Theory* section using the parameters given in Table S1. The improvement increases with the number of sampled points and the size of the protein. The latter dependence is explained by the decrease of the transverse relaxation time for large proteins. Table 2 lists the improvements in S/N ratio for ^13^C- and ^15^N-resolved NOESY spectra at the optimal resolution calculated at *t*_max_ = 1.26 *T*_2_ [31] for the six proteins. Figure S2 shows two NUS schedules sampling the indirect dimensions of ^13^C-resolved NOESY signals of a 33 kDa (2LQN) protein, and the difference between sampling schedules with or without repetitions of sampled points.

### Peak Counts

The peak counts gradually increase with increase in digital resolution for proteins of all sizes (Figure S1). For the 5 kDa protein, an increase in peak counts is observed until more than 1000 points are sampled. Similarly, for a protein of 10 kDa size, the count saturates after sampling about 600 points. The trend is similar for proteins of all sizes: it is inverse proportional to size. Larger proteins require smaller numbers of points for peak counts to saturate, beyond which there is no significant improvement in peak counts.

### Heavy Atom Root-Mean-Square Deviation (RMSD)

The protein structure dataset was divided into three classes based on the molecular sizes ranging between 10–15 kDa, 15–20 kDa, and 20–25 kDa. The median heavy-atom root-mean-square deviation (RMSD) of the protein structures varies with increasing digital resolution. Figure 2a shows changes in RMSD values for the three different size-groups. All groups show a similar trend for the RMSD profiles: high value at low resolution, then a rapid decrease, and a plateau after around 200 points. Protein structures with molecular sizes 10–15 kDa show a highest RMSD value of 1.88 Å when using 28 points and a lowest RMSD value of 1.14 Å at 331 points. Similarly, the corresponding high/low values for the size range 15–20 kDa are 3.01 Å and 1.57 Å. For the large size protein structures of 20–25 kDa, the RMSD values vary between 6.85 Å and 2.89 Å. It is clear that a roughly two-fold improvement in RMSD values at the highest resolution with respect to the lowest resolution can be obtained for proteins structures of all sizes.

A histogram of the final RMSD values of the calculated protein structures is shown in Figure 3. The majority of the RMSD values are below 2 Å with an average RMSD value of 1.63 Å for the whole dataset. A low Kolmogorov-Smirnov statistic value 0.0861 shows a statistically significant fit (Figure S3) between the histogram of the final RMSD values and the fitted gamma distribution. Table S2 shows the peak counts for ^13^C- and ^15^N-resolved NOESY peak lists, molecular weights, and various RMSD values for the 381 calculated protein structures.

### Chemical Shift Spectral Overlap Indices

Figure 2b shows the ^13^C- and ^15^N-resolved NOESY CSSO indices as a function of the number of sampled points for the protein structures grouped on the basis of molecular sizes. As expected, all groups show the highest peak overlap at the lowest resolution and the lowest peak overlap at the highest resolution. The average CSSO indices at the lowest resolution for the ^13^C- and ^15^N-resolved NOESY peak lists of all protein structures are 9 and 5 peaks, respectively. Essentially, this means that each peak is overlapped on average with 9 or 5 other peaks. The CSSO indices for all groups decrease as a function of resolution until a certain point. We refer to this as the critical point of digital resolution. The index remains stable beyond the critical resolution. It is interesting that the CSSO indices for the ^13^C-resolved NOESY peak lists for the protein structures of all sizes are around 1 peak at the highest digital resolution. The CSSO indices drop below 1 peak beyond a critical resolution for ^15^N-resolved NOESY peak-lists for protein structures of all sizes.

In order to assess the impact of digital resolution on the amount of peak overlap and on the RMSD values of calculated protein structures, the structures were grouped on the basis of the final heavy-atom RMSD values. Figure 4a and 4b show changes in the average CSSO indices for ^13^C- and ^15^N-resolved NOESY peak lists. With higher peak overlap, protein structures result in higher RMSD values whereas with lower peak overlap, lower RMSD values are observed. The highest CSSO index with 34.95 peaks is observed at the lowest resolution for the group of protein structures with RMSD values above 4 Å, which drops to 2.48 peaks at the highest resolution for ^13^C-resolved NOESY peak lists. For structures with RMSD values less than 1.5 Å, the CSSO index drops below 1 peak at the highest resolution, whereas for those with RMSD values higher than 4.0 Å, a significant change in the index between the lowest and the highest resolution is observed in both NOESY peak lists. It is also evident that no further decrease of CSSO index is possible beyond the critical resolutions for all protein structures. Table 1 lists the RMSD and ^13^C- and ^15^N-resolved NOESY CSSO indices as a function of the digital resolution obtained at varying numbers of points for three protein structures of different sizes. Table S3 lists CSSO indices for the ^13^C- and ^15^N-resolved NOESY peak lists obtained at three different numbers of points for 381 calculated protein structures.

**Table 1:** The 3D NOESY digital resolutions, RMSD values, and ^13^C- and ^15^N-resolved NOESY CSSO indices, for three calculated protein structures of different molecular sizes, are listed.

Protein PDB ID (Molecular weight)			1D5G (10 kDa)			1JBJ (15 kDa)			2JT2 (30 kDa)		
^1^H Linewidth (ppm)			0.0159			0.0328			0.0498		
	NOESY digital resolution (ppm)			NOESY CSSO index			NOESY CSSO index			NOESY CSSO index	
Number of points	^13^C	^15^N	RMSD	^13^C	^15^N	RMSD	^13^C	^15^N	RMSD	^13^C	^15^N
28	0.3927	0.4283	3.69	3.4	3.54	7.36	9.56	4.08	6.45	11.19	9.44
37	0.2971	0.3241	3.2	2.8	2.81	3.18	7.82	3.29	3.94	9.27	7.64
52	0.2114	0.230	2.48	2.18	2.11	2.37	5.98	2.43	2.33	7.17	5.72
74	0.1485	0.1620	2.12	1.74	1.57	2.08	4.56	1.81	1.84	5.51	4.31
101	0.1088	0.118	1.79	1.32	1.14	2.18	3.39	1.33	1.5	4.15	3.15
135	0.0814	0.0888	1.83	1.07	0.88	2.35	2.75	1.06	1.47	3.21	2.39
175	0.0628	0.068	1.74	0.95	0.74	2.06	2.28	0.83	1.51	2.71	1.95
221	0.0497	0.0542	1.72	0.81	0.6	2.03	1.99	0.73	1.38	1.44	0.9
273	0.0402	0.0439	1.67	0.73	0.48	2	1.7	0.63	1.49	1.44	0.9
331	0.0332	0.0362	1.64	0.65	0.39	2.68	1.13	0.38	1.43	1.44	0.9
395	0.0278	0.0303	1.76	0.61	0.34	2.57	1.13	0.38	1.47	1.44	0.9
466	0.0235	0.0257	1.81	0.58	0.3	2.5	1.13	0.38	1.39	1.44	0.9
542	0.0202	0.0221	1.65	0.55	0.23	2.14	1.13	0.38	1.46	1.44	0.9
625	0.0175	0.0191	1.7	0.55	0.23	2.64	1.13	0.38	1.44	1.44	0.9
714	0.0154	0.0167	1.84	0.44	0.12	2.25	1.13	0.38	1.39	1.44	0.9
809	0.0135	0.0148	1.73	0.44	0.12	2.03	1.13	0.38	1.41	1.44	0.9
910	0.0120	0.0131	1.73	0.44	0.12	2.03	1.13	0.38	1.39	1.44	0.9
1017	0.0108	0.0117	1.71	0.44	0.12	2.64	1.13	0.38	1.41	1.44	0.9
1130	0.0097	0.0106	1.71	0.44	0.12	2.68	1.13	0.38	1.38	1.44	0.9
1250	0.0087	0.0095	1.71	0.44	0.12	2.64	1.13	0.38	1.41	1.44	0.9

The linewidth corresponds to the ^1^H indirect dimension. The CSSO indices where the calculated linewidth becomes larger at critical and all subsequent higher digital resolutions, are underlined.

**Table 2:** Improvements in S/N for ^13^C- and ^15^N-resolved NOESY spectra at the optimal resolution calculated at *t*_max_ = 1.26 *T*_2_ [31].

			^13^C-resolved NOESY		^15^N-resolved NOESY	
PDB ID	Molecularweight, Da	*T*_2,_ s	*N*(*t*_max_)	Impr.S/N	*N*(*t*_max_)	Impr.S/N
2BBX	5440	0.061	1015	4.84	1102	5.04
1D5G	10008	0.033	552	3.57	599	3.72
1XKE	15117	0.022	365	2.90	396	3.03
1JBJ	20602	0.016	268	2.49	291	2.59
1TTE	24154	0.014	228	2.30	248	2.39
2JT2	31267	0.011	176	2.02	191	2.11

Numbers of points to be sampled correspond to the ^1^H indirect acquisition dimension.

### Adequate Digital Resolution as a Function of Molecular Weight

**Figure 5:**
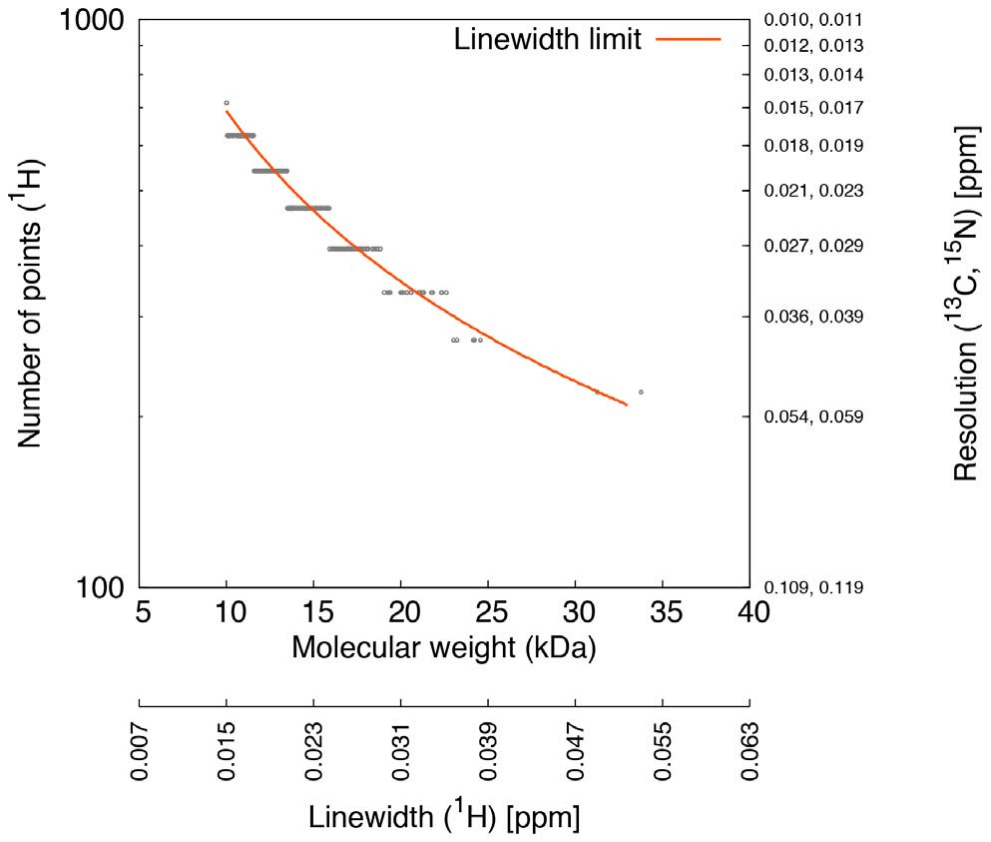
Adequate number of sampled data points as a function of the molecular weight. The adequate number of points is defined as the number of sampled points where the CSSO index stabilizes for the ^13^C-resolved NOESY peaks. Each open circle represents one protein. The red line indicates the number of points where the resolution becomes smaller than the linewidth. The digital resolutions corresponding to the number of sampled points are on the right vertical axis. Calculated linewidths of the ^1^H indirect dimension of ^13^C-resolved NOESY peaks are shown below the horizontal axis. The adequate number of points for each protein (open circles) is taken from the numbers of points chosen for the calculations of Table S1.

Figure 5 shows the number of points and the corresponding digital resolution adequate for sampling ^1^H indirect dimension of 3D NOESY spectra for 381 proteins with molecular size between 10 and 33 kDa. The minimal number of points that can be sampled decreases with increasing molecular weight. From the chosen numbers of points for this study, for a 10 kDa protein, minimally 714 points need to be sampled whereas the maximally possible resolution for a 33 kDa protein can be obtained by sampling 221 points. The number of points where the corresponding digital resolution becomes smaller than the calculated natural linewidth are indicated by a red line. The adequate resolutions for protein structures of three different sizes for ^13^C- and ^15^N-resolved NOESY spectra are highlighted in grey in Table 1. Therefore, the number of points that would cause the corresponding resolution to cross the natural linewidth limit may be referred to as the adequate number of points for a protein of a particular molecular size.

## Discussion

We studied the effects of digital resolution on the accuracy of protein structure calculations and on several other quantitative measures, namely, S/N ratios per unit of measurement time, peak counts, and CSSO indices.

The number of sampled points in the ^1^H indirect dimension primarily affects the spectral resolution. We employed NUS in both indirect dimensions of 3D NOESY experiments simultaneously, aiming at having better spectral resolution for both dimensions. The resolution in the ^1^H indirect dimension was varied from low to maximally possible in a realistic time period. The number of points was kept fixed at maximally 64 hyper-complex points in the ^13^C and ^15^N dimensions for two different reasons. In many experiments for backbone and side-chain resonance assignment, the constant-time acquisition in the ^15^N dimension limits the number of measured data points to about 80 complex points at most. In order to obtain a resolution in NOESY experiments that is consistent with that of the backbone and side-chain assignment set of experiments, we used 64 complex points in the ^13^C and ^15^N indirect dimensions. The second reason is, for the range of protein molecular sizes used in this work, owing to the high ^15^N signal dispersion, the presently set number of points is sufficient to resolve nearly all signals, provided that the ^1^H dimension is resolved. This number of points may not be sufficient in the ^13^C dimension for larger protein molecules. However, using the present resolution comparable to the best cases sampled uniformly, and similar to that used for ^15^N NOESY signals, the effects of changes of the ^1^H resolution can be studied more exhaustively. Since the resolution obtained using 64 complex points is not limited by the constant-time period, it could be increased for larger proteins.

The improvement in the S/N ratio (Figure 1 and Table 2) in comparison to uniform sampling is mainly due to exponential or matched sampling of NUS data. It increases with resolution and is observed for protein structures of all sizes. The comparative gain in signal sensitivity is particularly significant given the equal amount of noise obtained at fixed maximum acquisition time. The comparative gain in S/N ratio obtained as a function of digital resolution was studied for proteins of different molecular sizes. The rationale for the new sampling strategy of allowing repetitions of sampled points when generating sampling schedules with the *nussampler* program is that with repetitions of sampling points more signal is sampled at smaller acquisition times, which is more pronounced for quickly decaying magnetizations of large proteins.

Increasing digital resolution implies a decreasing separation between adjacent points in the frequency domain. The effect of this separation is reflected on peak counts (Figure S1) as more peaks can be separated from overlaps. Although the effect is observed for proteins of all sizes it is more pronounced for smaller proteins.

Higher digital resolution improves the accuracy of protein structures of all molecular sizes (Figure 2a). At higher digital resolutions, on average a nearly two-fold improvement relative to the lowest resolution can be obtained in RMSD values for protein structures of molecular sizes from 20 to 35 kDa. The improvement relative to a commonly used experimental setup may vary, however, it underscores the importance of using the higher digital resolution. Higher RMSD values are observed for proteins with larger molecular sizes, and lower values with smaller sizes. The drop in RMSD values is observed due to more structural information being available from many peaks as they become better resolved at higher digital resolutions. The CSSO indices for the ^13^C- and ^15^N-resolved NOESY peak lists in Figure 2b supports this result. The CSSO index quantifies the amount of peak overlap present at a given digital resolution. Thus, peaks become less overlapped due to higher evolution times, up to a critical resolution in the point range from 300 to 700. Above the critical digital resolution the peaks are maximally resolved. The number of points for achieving the critical resolution decreases with increasing molecular size. For instance, the numbers of points at critical resolutions for proteins with molecular sizes between 10 and 15 kDa are higher than those required for protein structures between 15 and 20 kDa, which in turn are higher than for sizes between 20 and 25 kDa. This means that small proteins can offer considerably more resolution, compared to what is normally practiced by NMR spectroscopists. It is also clear that peak resolution does not improve much beyond the critical digital resolution, which is close to the intrinsic natural linewidth defined by the transverse relaxation. Therefore, the high digital resolution provided by non-uniform sampling cannot improve the peak resolution beyond the natural linewidth. This can be observed in Figure S5 where the CSSO indices in all protein structures are seen stabilized beyond the natural linewidth resolution limit. For any molecular size, preferably a digital resolution corresponding to the natural linewidths of the molecule under study needs to be obtained for the maximum possible peak resolution. Hence, digital resolutions close or slightly above the limit of the natural linewidth may also be referred to as *adequate* digital resolutions. It should be noted that if no selective deuterium or other isotope labeling schemes such as SAIL [45], [46] are utilized, the natural linewidth is also limited by homonuclear coupling, which may exceed the linewidth defined by relaxation.

It is often argued that for small molecules full sampling at low resolution can give good results in terms of acceptable accuracy of the calculated protein structure. Nonetheless, as this work shows, higher resolutions can bring further improvement both for small and large molecules.

Some of the qualitative effects of better resolution might be difficult to quantify, for example, when it helps avoiding falsely assigned chemical shifts or obtaining structures with wrongly calculated parts, usually associated with misinterpretation due to peak overlap. Still, the degree of overlap could be a quantitative predictor of the resulting structural accuracy.

It is worthwhile noting that the CSSO index at highest digital resolution falls below 1 peak for ^15^N-resolved NOESY peak lists for protein structures of all sizes. This is important for the success of automated protein structure calculation methods as, if the majority of the peaks become non-overlapped, there appears a possibility of unambiguous peak-picking and consequently error-free peak assignment and structure calculation.

For digital resolutions obtained at small numbers of between 30 and 80 points, the peak overlap is high, and the RMSD values of the calculated protein structures are high. Structure calculations work well above resolutions obtained at around 200 points for different protein sizes and RMSD groups, beyond which there is nearly no improvement in the structure calculations. The *de novo* protein structure determination procedure in solution NMR spectroscopy has several steps. These include signal acquisition with sufficient time-domain sampling, data processing, peak picking, chemical shift assignments, obtaining inter-nuclear spatial upper distance limits, generation of torsion angle restraints, and finally the structure calculation. Higher resolution would help in nearly all of the steps. In particular, the determination of chemical shifts and their assignments have important consequences on the quality of resulting protein structure calculations [47]. High resolution is crucial for obtaining precision in measuring chemical shift values. Moreover, peak overlap severely impedes the performance of automated peak picking procedures and the precision of the chemical shift values. Both steps are essential for the unambiguous assignment of the chemical shift values and for the success of protein structure calculations. An improvement in the RMSD values of the calculated protein structures should have been observed to continue up to the critical resolution, as seen in Figure 2b. This contrasts with Figure 2a, where the calculated structures of any molecular size do not seem to improve significantly beyond sampling about 200 points. Yet any NOESY assignment algorithm, such as network-anchoring in CYANA [27], will not be able to deal with very high overlap, as observed at resolutions obtained at around 100 points here.

The conclusion is to use an adequate digital resolution, equal or slightly above the limit of the intrinsic or natural linewidth specific to a protein molecule, as it can help minimize peak overlap and thus improve the accuracy of chemical shift values and protein structures. Apart from the chosen numbers of points in this study, the exact numbers of points adequate for a protein of a particular size would depend on the resolution where the natural linewidth of the indirectly sampled dimension exceeds the corresponding digital resolution. The detailed analysis given in the present study provides a basis for the optimal choice of adequate digital resolutions necessary for enhanced accuracy of protein structure calculations of varying sizes. This may become an important step towards raising the current molecular size limit for protein structures that can be solved by solution NMR spectroscopy.

## Supporting Information

Figure S1 **Peak count numbers for ^13^C- and ^15^N-resolved NOESY peak lists**. Peak count numbers are shown for protein structures for various molecular sizes ranging from 5 kDa to 30 kDa. (A) Peak count numbers for ^13^C-resolved NOESY peak lists. (B) Peak count numbers for ^15^N-resolved NOESY peak lists.
(PDF)

Figure S2 **NUS sampling schedules for a large protein**. Two NUS sampling schedules for the indirect dimensions of ^13^C-resolved NOESY signals of a 33 kDa protein (2LQN) with and without repetitions of sampled points are shown. Color codes represent the frequency of the repetition of a point. Red, green, blue, magenta, and grey dots indicate two, three, four, five, and no repetitions, respectively.
(PDF)

Figure S3 **Gamma distribution plot for the final RMSD values and theoretical quantiles plot.** The final RMSD values are fitted to a gamma distribution using maximum likelihood fitting. Data on X-axis stands for the final RMSD values and density on Y-axis represents the probability of distribution density. The reference distribution (gamma distribution) is plotted using cumulative distribution function. QQ-plot represents theoretical quantiles and PP-plot represents theoretical probabilities of the final RMSD data.
(PDF)

Figure S4 **Heavy-atom RMSD values of calculated protein structures.** Heavy-atom RMSD values of calculated structures to corresponding reference structures are plotted as a function of number of sampled points for separately for all protein molecules the dataset. Values in brackets in figure legends refer to the molecular weight of protein molecules in Dalton.
(PDF)

Figure S5 **Chemical shift spectral overlap index for 13C-resolved NOESY peak lists.** Chemical shift spectral overlap index for ^13^C-resolved NOESY peak lists is plotted as a function of number of sampled points. Values in brackets in figure legends refer to the molecular weight of protein molecules in Dalton.
(PDF)

Table S1 Parameters for simulation and preparation of ^13^C- and ^15^N-resolved NOESY peak lists obtained from experimental data.
(PDF)

Table S2 Peak counts for ^13^C- and ^15^N-resolved NOESY peak lists, molecular weight, and various RMSD values for 381 calculated protein structures.
(PDF)

Table S3 Chemical shift spectral overlap indices for the ^13^C- and ^15^N-resolved NOESY peak lists at various resolutions for 381 calculated protein structures.
(PDF)
