# Supplementary figures and images for: Effects of NMR Spectral Resolution on Protein Structure Calculation
Source: PLoS One. 2013 Jul 16;8(7):e68567. doi: 10.1371/journal.pone.0068567 (PMC3713035; doi:10.1371/journal.pone.0068567)

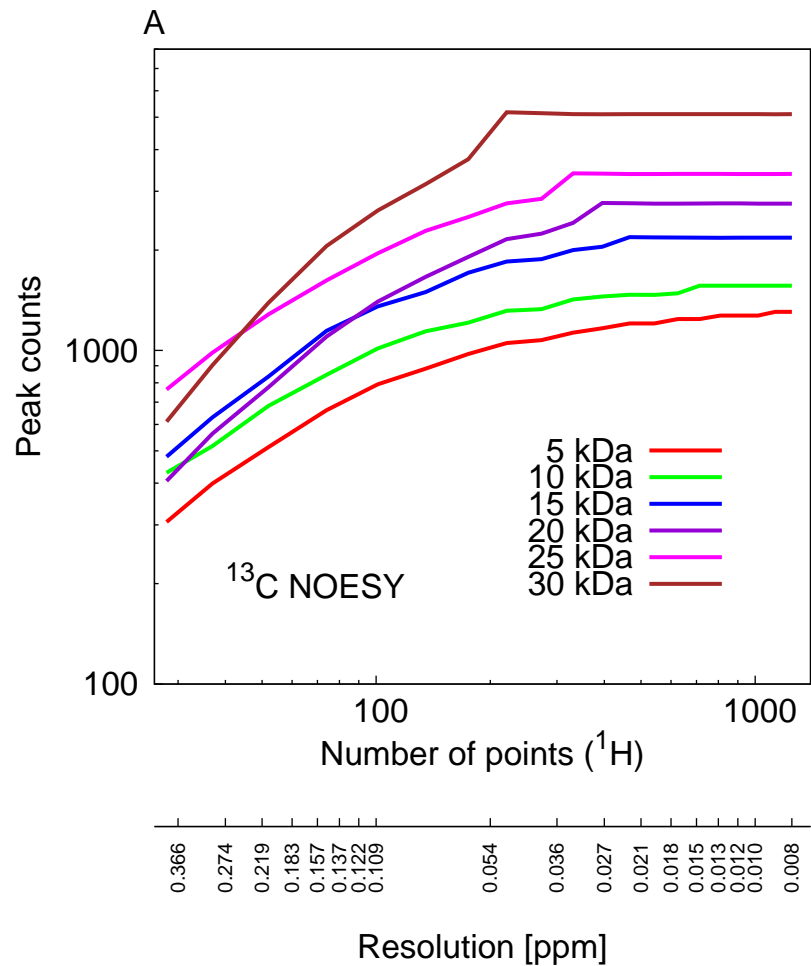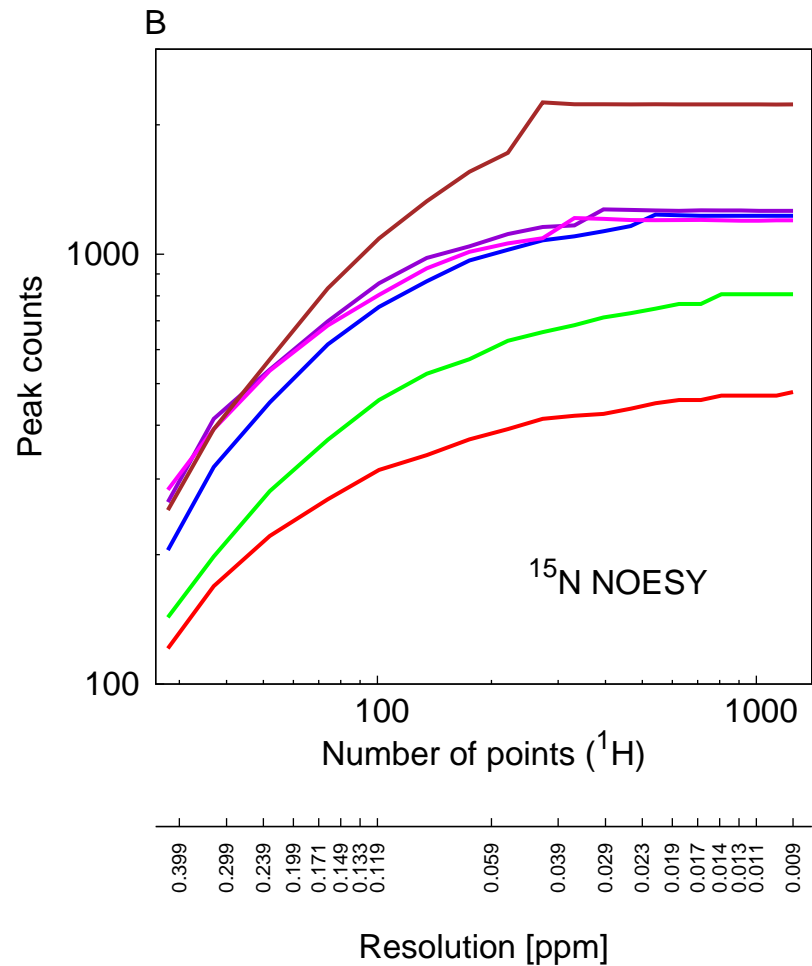

Supplement: Figure S1 — Peak count numbers for 13C- and 15N-resolved NOESY peak lists. Peak count numbers are shown for protein structures for various molecular sizes ranging from 5 kDa to 30 kDa. (A) Peak count numbers for 13C-resolved NOESY peak lists. (B) Peak count numbers for 15N-resolved NOESY peak lists. (PDF) [file pone.0068567.s001.pdf]

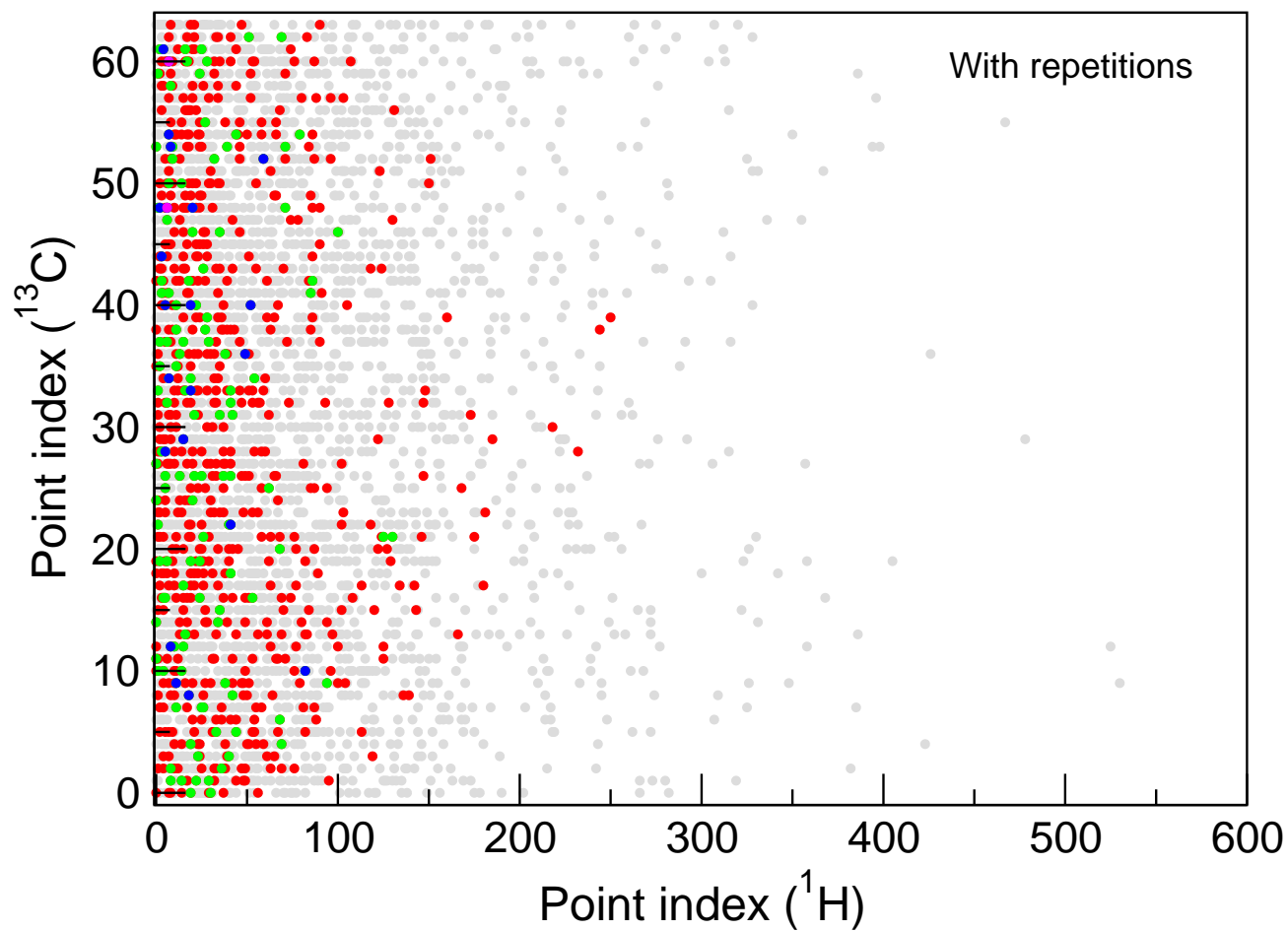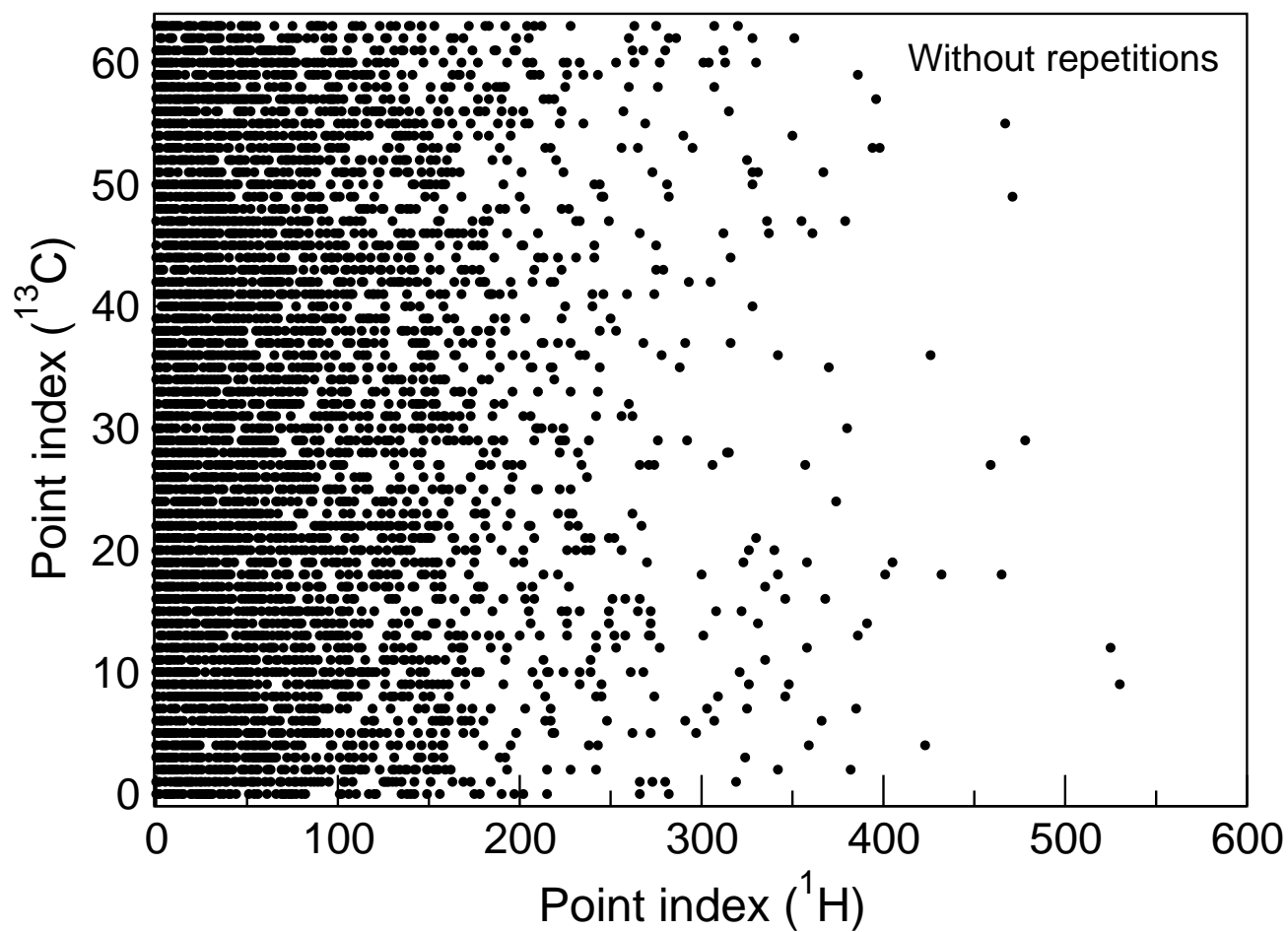

Supplement: Figure S2 — NUS sampling schedules for a large protein. Two NUS sampling schedules for the indirect dimensions of 13C-resolved NOESY signals of a 33 kDa protein (2LQN) with and without repetitions of sampled points are shown. Color codes represent the frequency of the repetition of a point. Red, green, blue, magenta, and grey dots indicate two, three, four, five, and no repetitions, respectively. (PDF) [file pone.0068567.s002.pdf]

**Empirical and theoretical distr.**

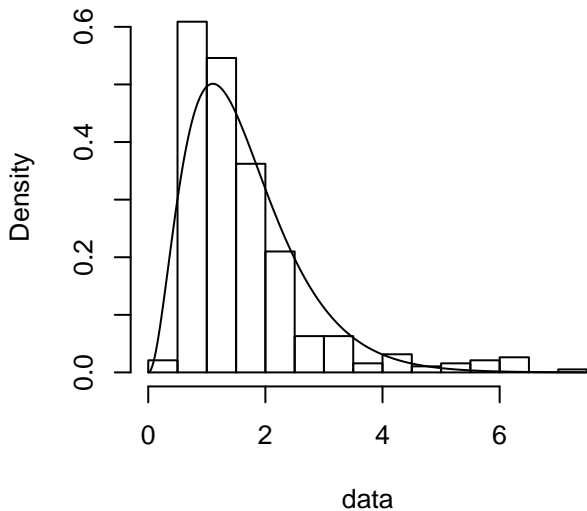

**QQ-plot**

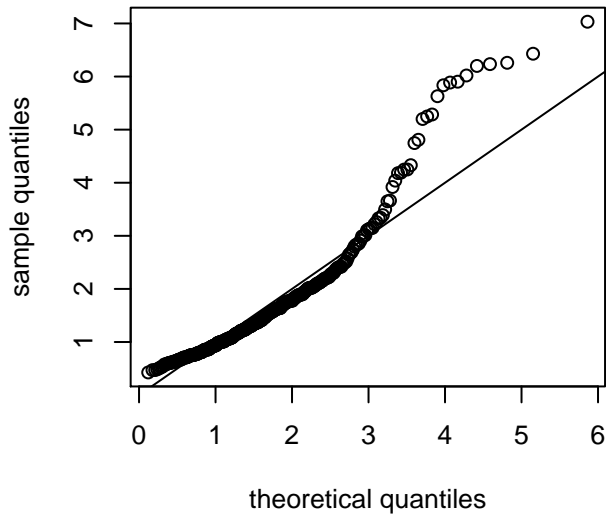

**Empirical and theoretical CDFs**

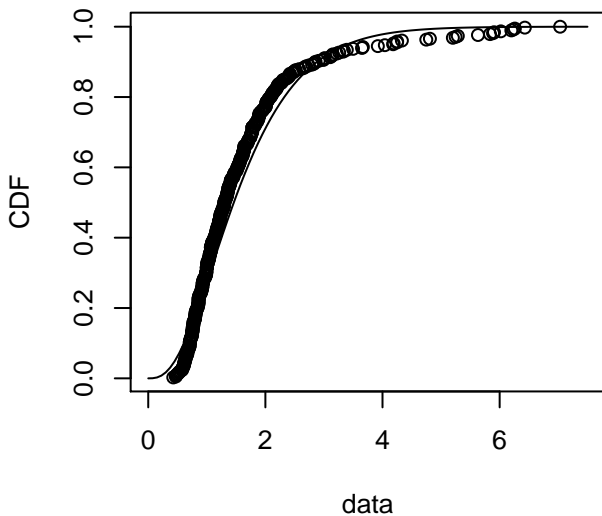

**PP-plot**

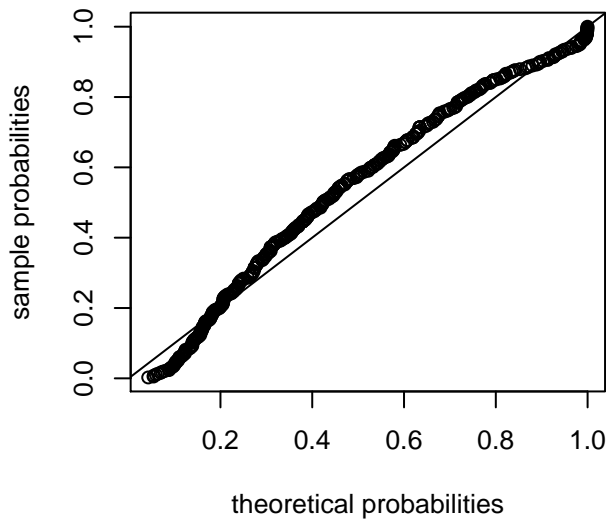

Supplement: Figure S3 — Gamma distribution plot for the final RMSD values and theoretical quantiles plot. The final RMSD values are fitted to a gamma distribution using maximum likelihood fitting. Data on X-axis stands for the final RMSD values and density on Y-axis represents the probability of distribution density. The reference distribution (gamma distribution) is plotted using cumulative distribution function. QQ-plot represents theoretical quantiles and PP-plot represents theoretical probabilities of the final RMSD data. (PDF) [file pone.0068567.s003.pdf]
