# Supplementary material for: Effects of NMR Spectral Resolution on Protein Structure Calculation
Source: PLoS One. 2013 Jul 16;8(7):e68567. doi: 10.1371/journal.pone.0068567 (PMC3713035; doi:10.1371/journal.pone.0068567)
Supplement: Figure S4 — Heavy-atom RMSD values of calculated protein structures. Heavy-atom RMSD values of calculated structures to corresponding reference structures are plotted as a function of number of sampled points for separately for all protein molecules the dataset. Values in brackets in figure legends refer to the molecular weight of protein molecules in Dalton. (PDF) [file pone.0068567.s004.pdf]

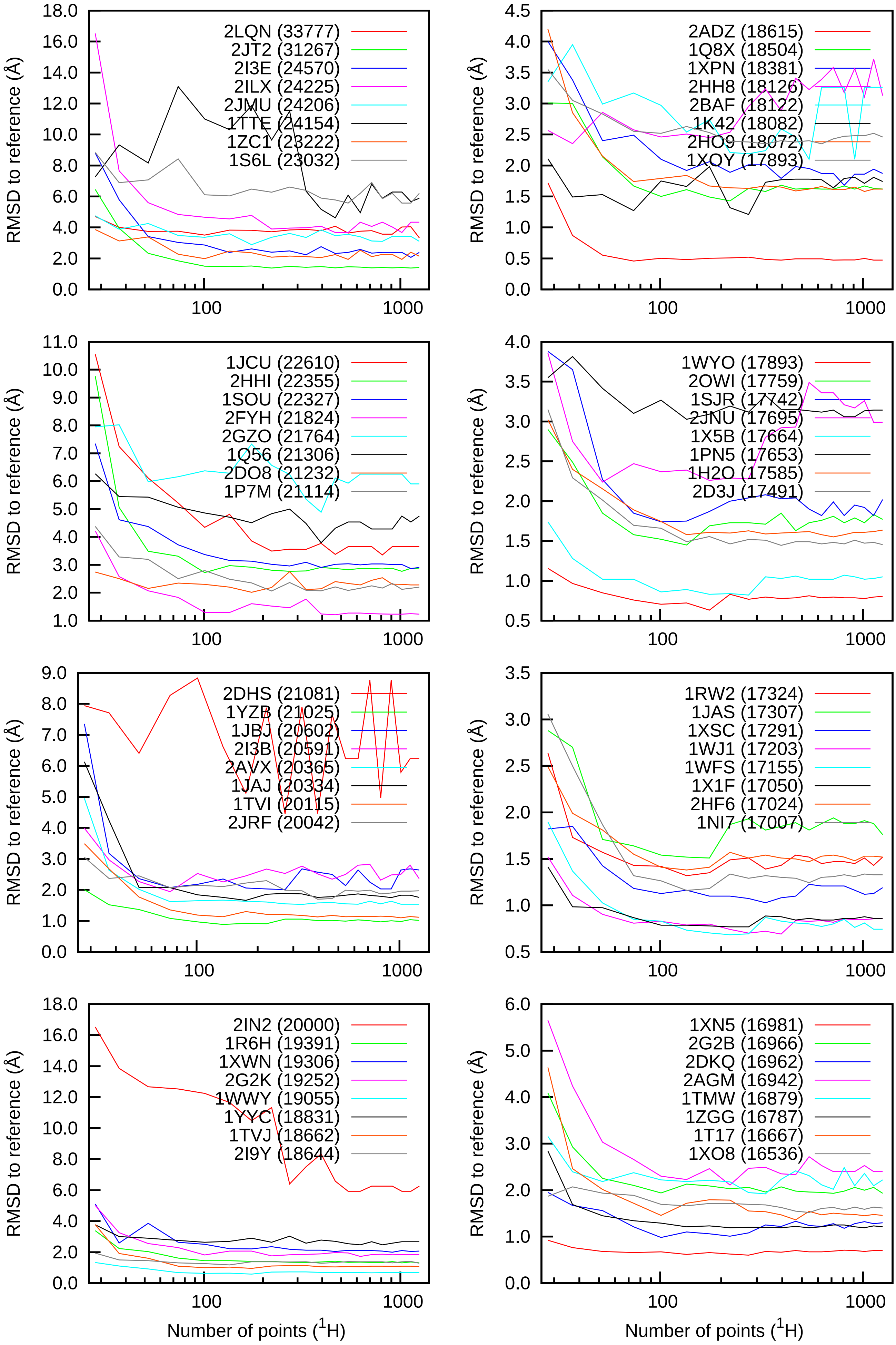

RMSD to reference (Å)
Number of points (^{1}H)
2LQN (33777)
2JT2 (31267)
2I3E (24570)
2ILX (24225)
2JMU (24206)
1TTE (24154)
1ZC1 (23222)
1S6L (23032)
2ADZ (18615)
1Q8X (18504)
1XPN (18381)
2HH8 (18126)
2BAF (18122)
1K42 (18082)
2HO9 (18072)
1XQY (17893)
1JCU (22610)
2HHI (22355)
1SOU (22327)
2FYH (21824)
2GZO (21764)
1Q56 (21306)
2DO8 (21232)
1P7M (21114)
1WYO (17893)
2OWI (17759)
1SJR (17742)
2JNU (17695)
1X5B (17664)
1PN5 (17653)
1H2O (17585)
2D3J (17491)
2DHS (21081)
1YZB (21025)
1JBJ (20602)
2I3B (20591)
2AVX (20365)
1JAJ (20334)
1TVI (20115)
2JRF (20042)
1RW2 (17324)
1JAS (17307)
1XSC (17291)
1WJ1 (17203)
1WFS (17155)
1X1F (17050)
2HF6 (17024)
1NI7 (17007)
2IN2 (20000)
1R6H (19391)
1XWN (19306)
2G2K (19252)
1WWY (19055)
1YYC (18831)
1TVJ (18662)
2I9Y (18644)
1XN5 (16981)
2G2B (16966)
2DKQ (16962)
2AGM (16942)
1TMW (16879)
1ZGG (16787)
1T17 (16667)
1XO8 (16536)

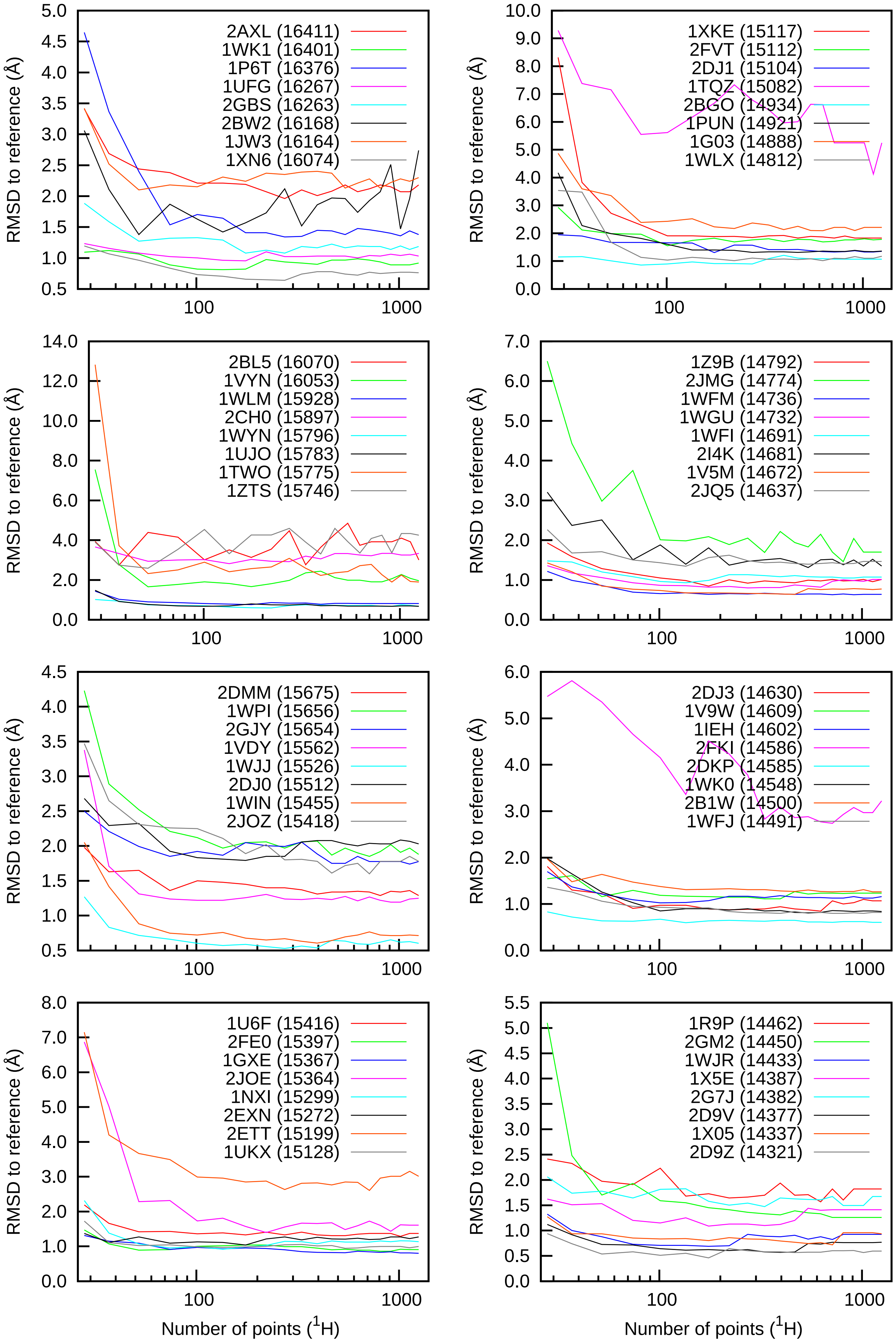

RMSD to reference (Å)
2AXL (16411)
1WK1 (16401)
1P6T (16376)
1UFG (16267)
2GBS (16263)
2BW2 (16168)
1JW3 (16164)
1XN6 (16074)
1XKE (15117)
2FVT (15112)
2DJ1 (15104)
1TQZ (15082)
2BGO (14934)
1PUN (14921)
1G03 (14888)
1WLX (14812)
2BL5 (16070)
1VYN (16053)
1WLM (15928)
2CH0 (15897)
1WYN (15796)
1UJO (15783)
1TWO (15775)
1ZTS (15746)
1Z9B (14792)
2JMG (14774)
1WFM (14736)
1WGU (14732)
1WFI (14691)
2I4K (14681)
1V5M (14672)
2JQ5 (14637)
2DMM (15675)
1WPI (15656)
2GJY (15654)
1VDY (15562)
1WJJ (15526)
2DJ0 (15512)
1WIN (15455)
2JOZ (15418)
2DJ3 (14630)
1V9W (14609)
1IEH (14602)
2FKI (14586)
2DKP (14585)
1WK0 (14548)
2B1W (14500)
1WFJ (14491)
1U6F (15416)
2FE0 (15397)
1GXE (15367)
2JOE (15364)
1NXI (15299)
2EXN (15272)
2ETT (15199)
1UKX (15128)
1R9P (14462)
2GM2 (14450)
1WJR (14433)
1X5E (14387)
2G7J (14382)
2D9V (14377)
1X05 (14337)
2D9Z (14321)
Number of points (^{1}H)

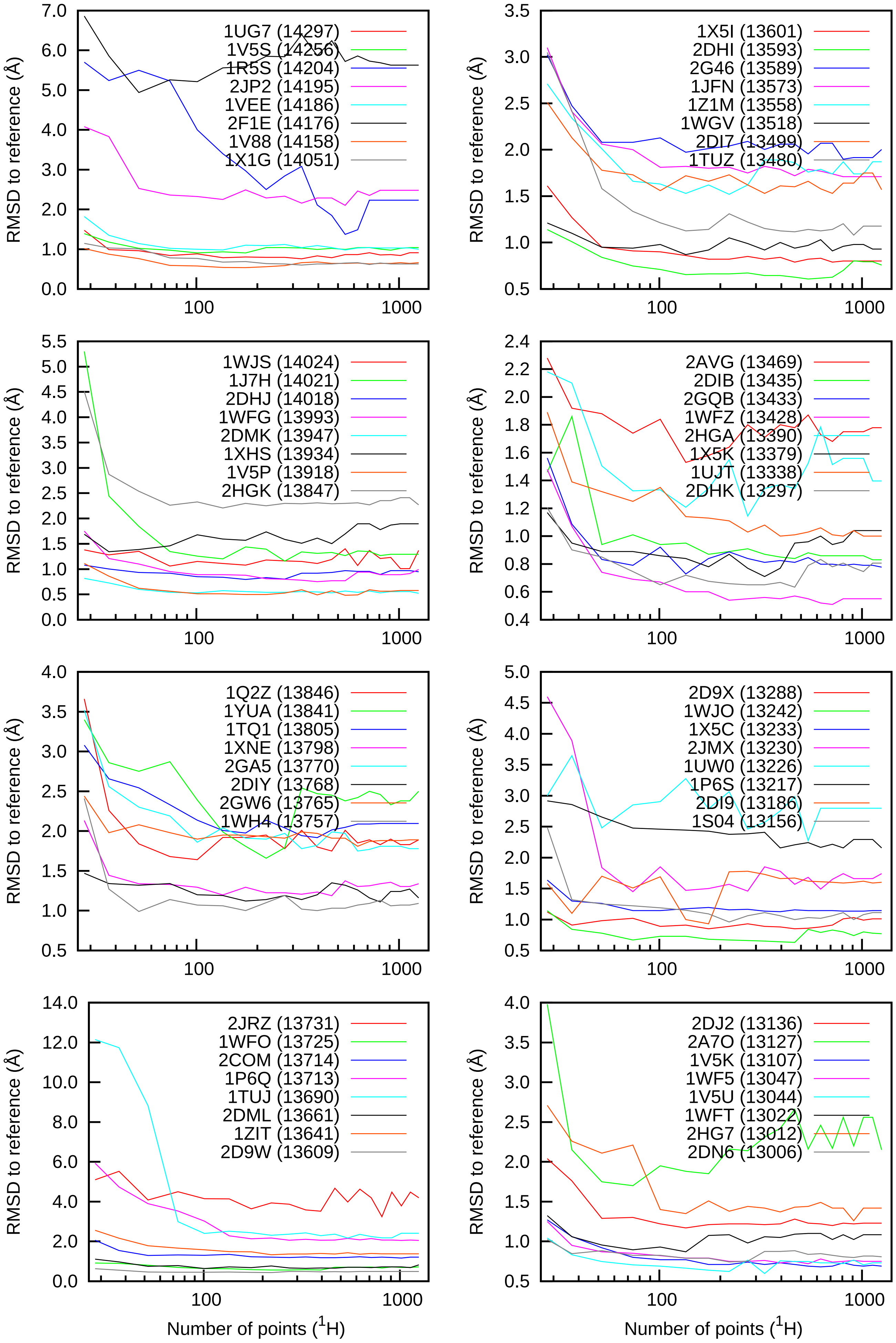

1UG7 (14297)
1V5S (14256)
1R5S (14204)
2JP2 (14195)
1VEE (14186)
2F1E (14176)
1V88 (14158)
1X1G (14051)
1X5I (13601)
2DHI (13593)
2G46 (13589)
1JFN (13573)
1Z1M (13558)
1WGV (13518)
2DI7 (13499)
1TUZ (13480)
1WJS (14024)
1J7H (14021)
2DHJ (14018)
1WFG (13993)
2DMK (13947)
1XHS (13934)
1V5P (13918)
2HGK (13847)
2AVG (13469)
2DIB (13435)
2GQB (13433)
1WFZ (13428)
2HGA (13390)
1X5K (13379)
1UJT (13338)
2DHK (13297)
1Q2Z (13846)
1YUA (13841)
1TQ1 (13805)
1XNE (13798)
2GA5 (13770)
2DIY (13768)
2GW6 (13765)
1WH4 (13757)
2D9X (13288)
1WJO (13242)
1X5C (13233)
2JMX (13230)
1UW0 (13226)
1P6S (13217)
2DI9 (13186)
1S04 (13156)
2JRZ (13731)
1WFO (13725)
2COM (13714)
1P6Q (13713)
1TUJ (13690)
2DML (13661)
1ZIT (13641)
2D9W (13609)
2DJ2 (13136)
2A7O (13127)
1V5K (13107)
1WF5 (13047)
1V5U (13044)
1WFT (13022)
2HG7 (13012)
2DN6 (13006)
RMSD to reference (Å)
Number of points (1H)

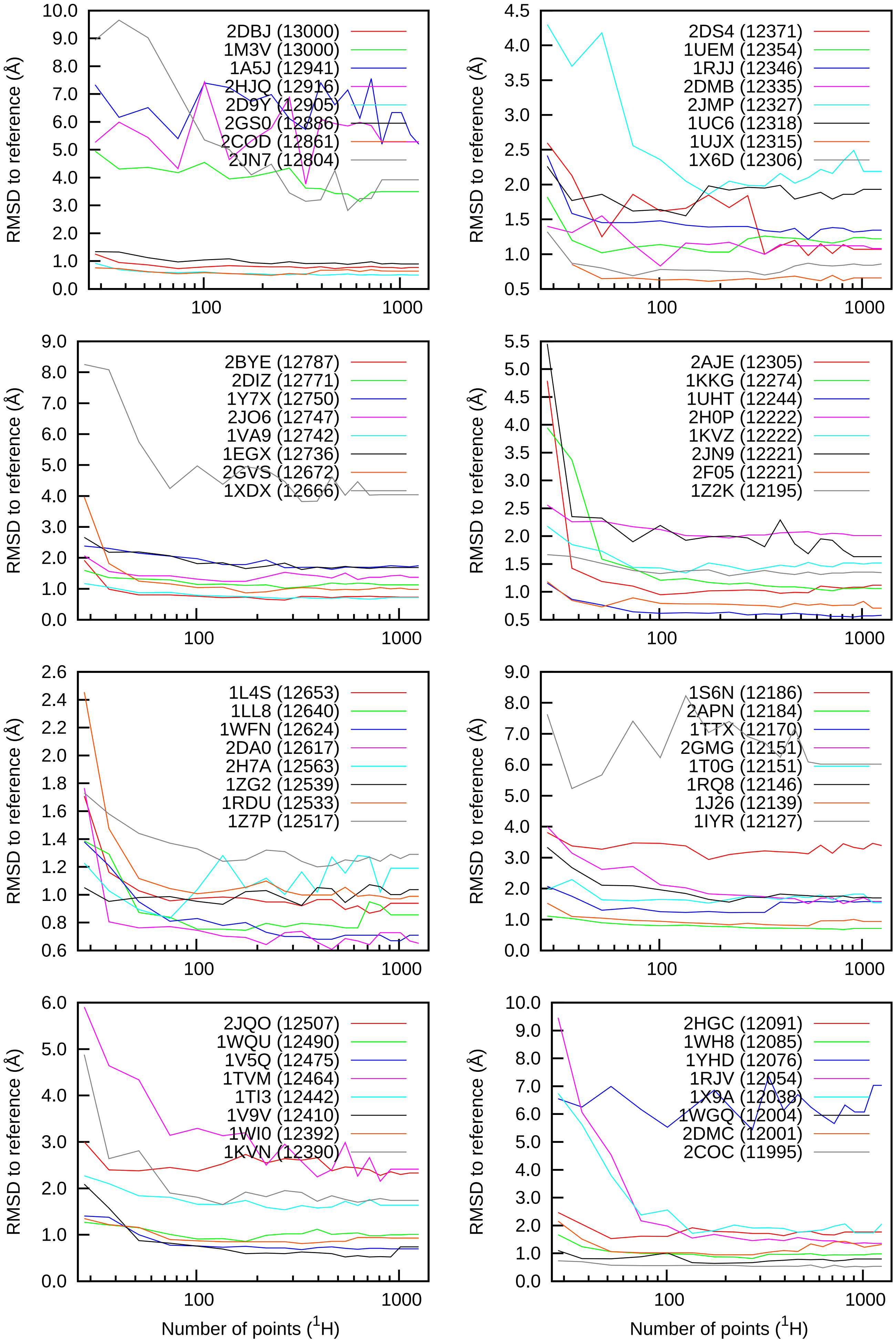

RMSD to reference (Å)
2DBJ (13000)
1M3V (13000)
1A5J (12941)
2HJQ (12916)
2D9Y (12905)
2GS0 (12886)
2COD (12861)
2JN7 (12804)
2DS4 (12371)
1UEM (12354)
1RJJ (12346)
2DMB (12335)
2JMP (12327)
1UC6 (12318)
1UJX (12315)
1X6D (12306)
2BYE (12787)
2DIZ (12771)
1Y7X (12750)
2JO6 (12747)
1VA9 (12742)
1EGX (12736)
2GVS (12672)
1XDX (12666)
2AJE (12305)
1KKG (12274)
1UHT (12244)
2H0P (12222)
1KVZ (12222)
2JN9 (12221)
2F05 (12221)
1Z2K (12195)
1L4S (12653)
1LL8 (12640)
1WFN (12624)
2DA0 (12617)
2H7A (12563)
1ZG2 (12539)
1RDU (12533)
1Z7P (12517)
1S6N (12186)
2APN (12184)
1TTX (12170)
2GMG (12151)
1T0G (12151)
1RQ8 (12146)
1J26 (12139)
1IYR (12127)
2JQO (12507)
1WQU (12490)
1V5Q (12475)
1TVM (12464)
1TI3 (12442)
1V9V (12410)
1WI0 (12392)
1KVN (12390)
2HGC (12091)
1WH8 (12085)
1YHD (12076)
1RJV (12054)
1X9A (12038)
1WGQ (12004)
2DMC (12001)
2COC (11995)
100
1000
Number of points (1H)

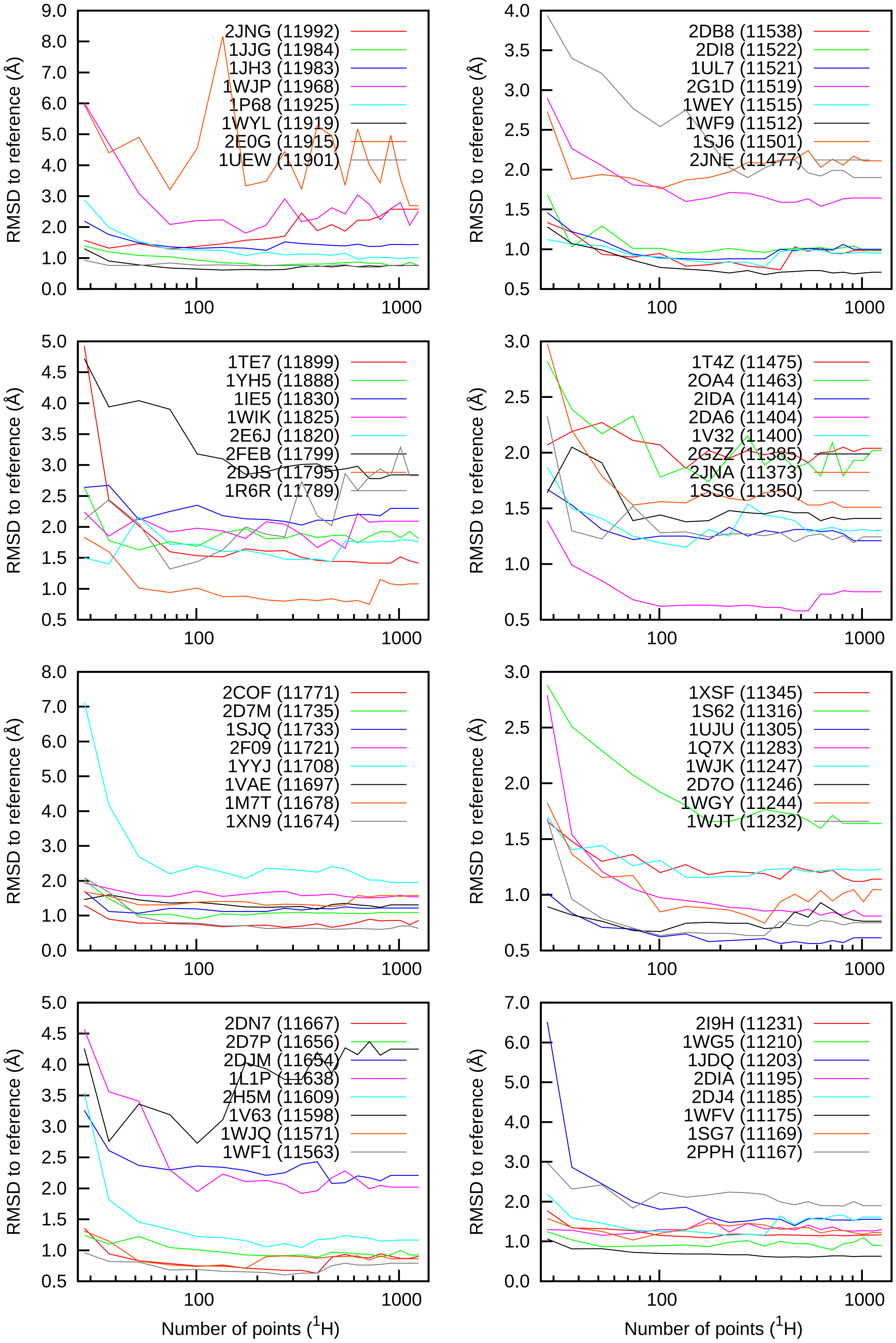
RMSD to reference (Å)
Number of points (^{1}H)
100
1000
2JNG (11992)
1JJG (11984)
1JH3 (11983)
1WJP (11968)
1P68 (11925)
1WYL (11919)
2E0G (11915)
1UEW (11901)
2DB8 (11538)
2DI8 (11522)
1UL7 (11521)
2G1D (11519)
1WEY (11515)
1WF9 (11512)
1SJ6 (11501)
2JNE (11477)
1TE7 (11899)
1YH5 (11888)
1IE5 (11830)
1WIK (11825)
2E6J (11820)
2FEB (11799)
2DJS (11795)
1R6R (11789)
1T4Z (11475)
2OA4 (11463)
2IDA (11414)
2DA6 (11404)
1V32 (11400)
2GZZ (11385)
2JNA (11373)
1SS6 (11350)
2COF (11771)
2D7M (11735)
1SJQ (11733)
2F09 (11721)
1YYJ (11708)
1VAE (11697)
1M7T (11678)
1XN9 (11674)
1XSF (11345)
1S62 (11316)
1UJU (11305)
1Q7X (11283)
1WJK (11247)
2D7O (11246)
1WGY (11244)
1WJT (11232)
2DN7 (11667)
2D7P (11656)
2DJM (11654)
1L1P (11638)
2H5M (11609)
1V63 (11598)
1WJQ (11571)
1WF1 (11563)
2I9H (11231)
1WG5 (11210)
1JDQ (11203)
2DIA (11195)
2DJ4 (11185)
1WFV (11175)
1SG7 (11169)
2PPH (11167)

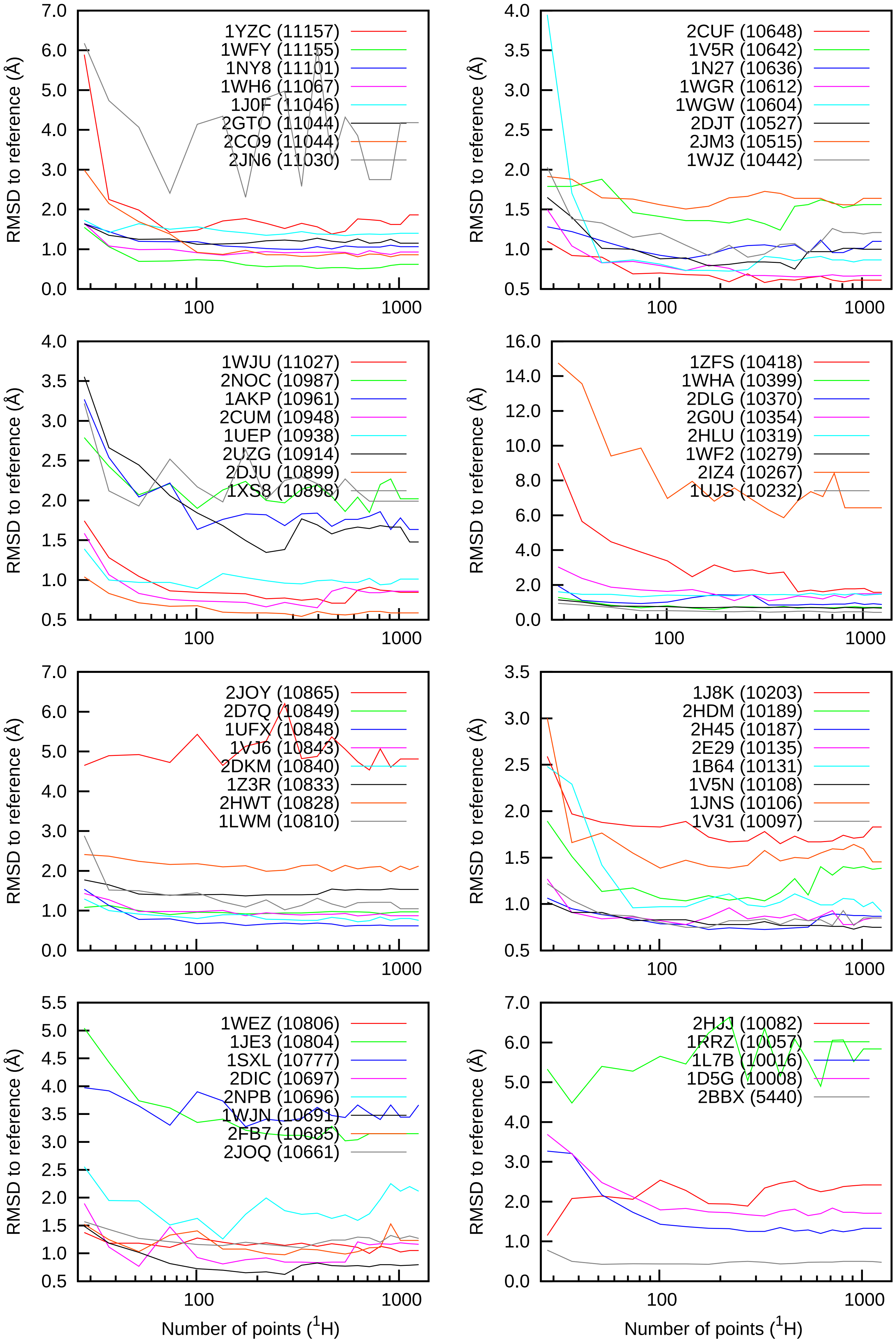
RMSD to reference (Å)
Number of points (^{1}H)
100
1000
1YZC (11157)
1WFY (11155)
1NY8 (11101)
1WH6 (11067)
1J0F (11046)
2GTO (11044)
2CO9 (11044)
2JN6 (11030)
2CUF (10648)
1V5R (10642)
1N27 (10636)
1WGR (10612)
1WGW (10604)
2DJT (10527)
2JM3 (10515)
1WJZ (10442)
1WJU (11027)
2NOC (10987)
1AKP (10961)
2CUM (10948)
1UEP (10938)
2UZG (10914)
2DJU (10899)
1XS8 (10898)
1ZFS (10418)
1WHA (10399)
2DLG (10370)
2G0U (10354)
2HLU (10319)
1WF2 (10279)
2IZ4 (10267)
1UJS (10232)
2JOY (10865)
2D7Q (10849)
1UFX (10848)
1VJ6 (10843)
2DKM (10840)
1Z3R (10833)
2HWT (10828)
1LWM (10810)
1J8K (10203)
2HDM (10189)
2H45 (10187)
2E29 (10135)
1B64 (10131)
1V5N (10108)
1JNS (10106)
1V31 (10097)
1WEZ (10806)
1JE3 (10804)
1SXL (10777)
2DIC (10697)
2NPB (10696)
1WJN (10691)
2FB7 (10685)
2JOQ (10661)
2HJJ (10082)
1RRZ (10057)
1L7B (10016)
1D5G (10008)
2BBX (5440)
