# Supplementary material for: Effects of NMR Spectral Resolution on Protein Structure Calculation
Source: PLoS One. 2013 Jul 16;8(7):e68567. doi: 10.1371/journal.pone.0068567 (PMC3713035; doi:10.1371/journal.pone.0068567)
Supplement: Figure S5 — Chemical shift spectral overlap index for 13C-resolved NOESY peak lists. Chemical shift spectral overlap index for 13C-resolved NOESY peak lists is plotted as a function of number of sampled points. Values in brackets in figure legends refer to the molecular weight of protein molecules in Dalton. (PDF) [file pone.0068567.s005.pdf]

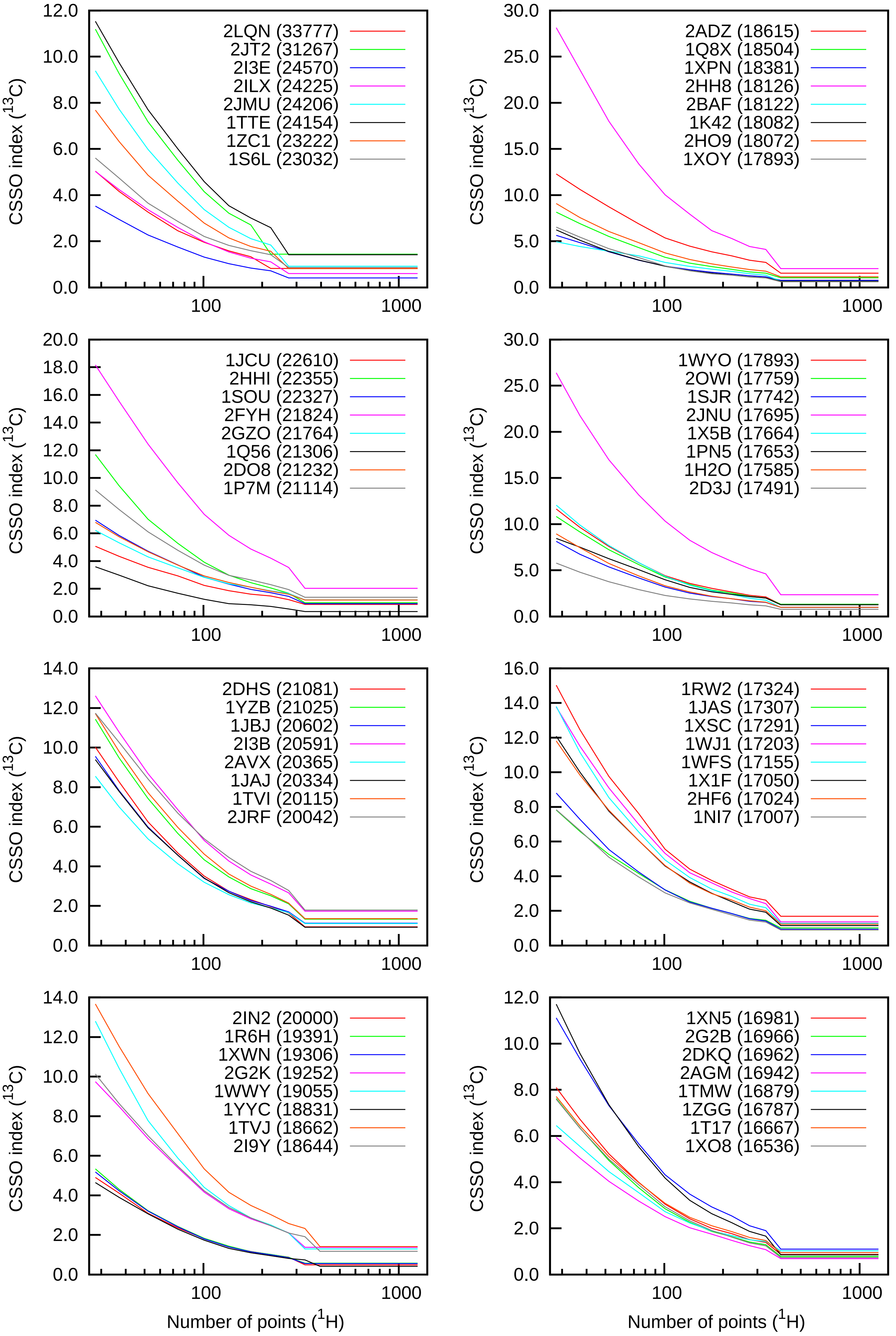

CSSO index (^{13}C)
Number of points (^{1}H)
100
1000
2LQN (33777)
2JT2 (31267)
2I3E (24570)
2ILX (24225)
2JMU (24206)
1TTE (24154)
1ZC1 (23222)
1S6L (23032)
2ADZ (18615)
1Q8X (18504)
1XPN (18381)
2HH8 (18126)
2BAF (18122)
1K42 (18082)
2HO9 (18072)
1XOY (17893)
1JCU (22610)
2HHI (22355)
1SOU (22327)
2FYH (21824)
2GZO (21764)
1Q56 (21306)
2DO8 (21232)
1P7M (21114)
1WYO (17893)
2OWI (17759)
1SJR (17742)
2JNU (17695)
1X5B (17664)
1PN5 (17653)
1H2O (17585)
2D3J (17491)
2DHS (21081)
1YZB (21025)
1JBJ (20602)
2I3B (20591)
2AVX (20365)
1JAJ (20334)
1TVI (20115)
2JRF (20042)
1RW2 (17324)
1JAS (17307)
1XSC (17291)
1WJ1 (17203)
1WFS (17155)
1X1F (17050)
2HF6 (17024)
1NI7 (17007)
2IN2 (20000)
1R6H (19391)
1XWN (19306)
2G2K (19252)
1WWY (19055)
1YYC (18831)
1TVJ (18662)
2I9Y (18644)
1XN5 (16981)
2G2B (16966)
2DKQ (16962)
2AGM (16942)
1TMW (16879)
1ZGG (16787)
1T17 (16667)
1XO8 (16536)

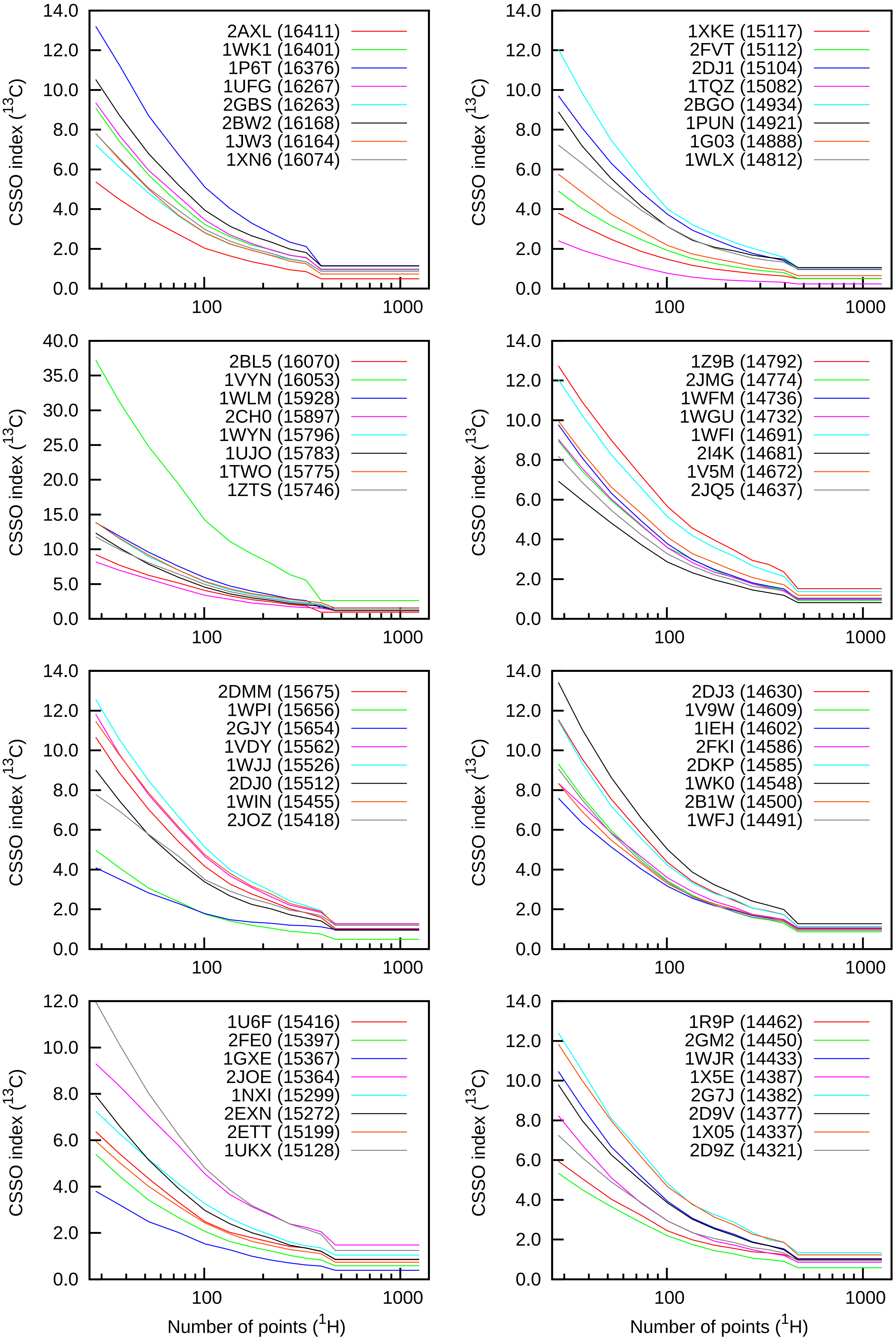
CSSO index (^{13}C)
Number of points (^{1}H)
2AXL (16411)
1WK1 (16401)
1P6T (16376)
1UFG (16267)
2GBS (16263)
2BW2 (16168)
1JW3 (16164)
1XN6 (16074)
1XKE (15117)
2FVT (15112)
2DJ1 (15104)
1TQZ (15082)
2BGO (14934)
1PUN (14921)
1G03 (14888)
1WLX (14812)
2BL5 (16070)
1VYN (16053)
1WLM (15928)
2CH0 (15897)
1WYN (15796)
1UJO (15783)
1TWO (15775)
1ZTS (15746)
1Z9B (14792)
2JMG (14774)
1WFM (14736)
1WGU (14732)
1WFI (14691)
2I4K (14681)
1V5M (14672)
2JQ5 (14637)
2DMM (15675)
1WPI (15656)
2GJY (15654)
1VDY (15562)
1WJJ (15526)
2DJ0 (15512)
1WIN (15455)
2JOZ (15418)
2DJ3 (14630)
1V9W (14609)
1IEH (14602)
2FKI (14586)
2DKP (14585)
1WK0 (14548)
2B1W (14500)
1WFJ (14491)
1U6F (15416)
2FE0 (15397)
1GXE (15367)
2JOE (15364)
1NXI (15299)
2EXN (15272)
2ETT (15199)
1UKX (15128)
1R9P (14462)
2GM2 (14450)
1WJR (14433)
1X5E (14387)
2G7J (14382)
2D9V (14377)
1X05 (14337)
2D9Z (14321)

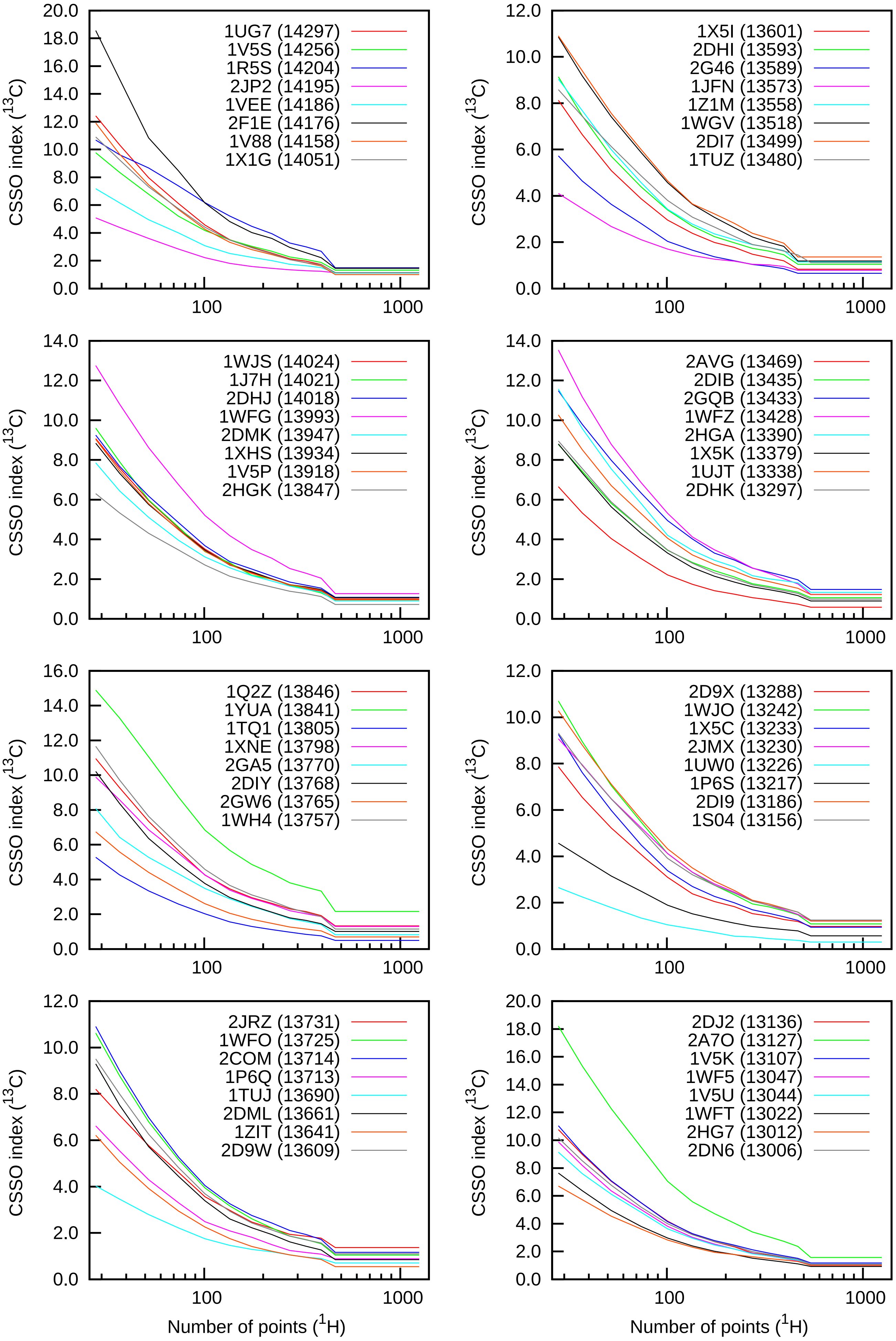
CSSO index (13C)
Number of points (1H)
1UG7 (14297)
1V5S (14256)
1R5S (14204)
2JP2 (14195)
1VEE (14186)
2F1E (14176)
1V88 (14158)
1X1G (14051)
1X5I (13601)
2DHI (13593)
2G46 (13589)
1JFN (13573)
1Z1M (13558)
1WGV (13518)
2DI7 (13499)
1TUZ (13480)
1WJS (14024)
1J7H (14021)
2DHJ (14018)
1WFG (13993)
2DMK (13947)
1XHS (13934)
1V5P (13918)
2HGK (13847)
2AVG (13469)
2DIB (13435)
2GQB (13433)
1WFZ (13428)
2HGA (13390)
1X5K (13379)
1UJT (13338)
2DHK (13297)
1Q2Z (13846)
1YUA (13841)
1TQ1 (13805)
1XNE (13798)
2GA5 (13770)
2DIY (13768)
2GW6 (13765)
1WH4 (13757)
2D9X (13288)
1WJO (13242)
1X5C (13233)
2JMX (13230)
1UW0 (13226)
1P6S (13217)
2DI9 (13186)
1S04 (13156)
2JRZ (13731)
1WFO (13725)
2COM (13714)
1P6Q (13713)
1TUJ (13690)
2DML (13661)
1ZIT (13641)
2D9W (13609)
2DJ2 (13136)
2A7O (13127)
1V5K (13107)
1WF5 (13047)
1V5U (13044)
1WFT (13022)
2HG7 (13012)
2DN6 (13006)

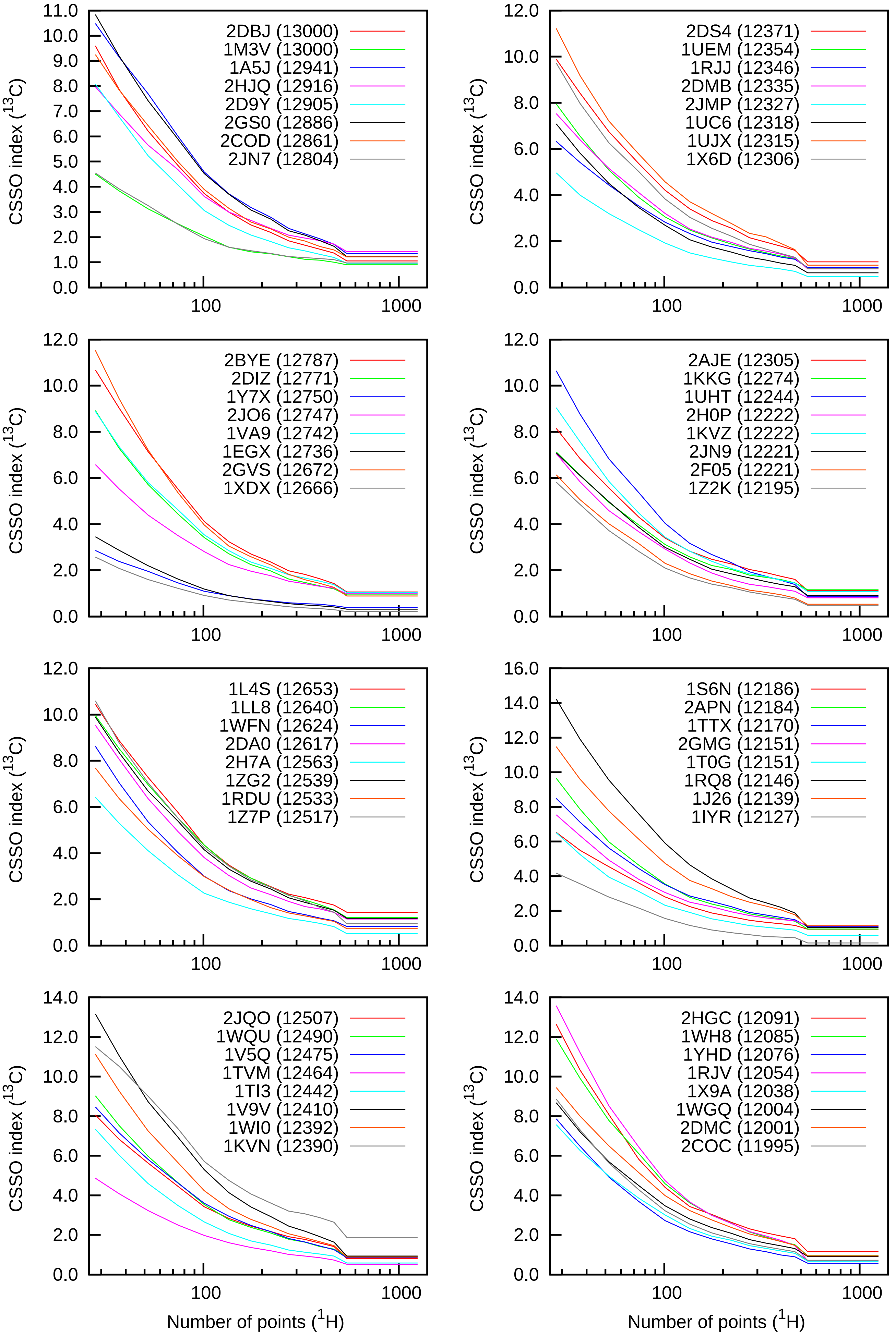

2DBJ (13000)
1M3V (13000)
1A5J (12941)
2HJQ (12916)
2D9Y (12905)
2GS0 (12886)
2COD (12861)
2JN7 (12804)
2DS4 (12371)
1UEM (12354)
1RJJ (12346)
2DMB (12335)
2JMP (12327)
1UC6 (12318)
1UJX (12315)
1X6D (12306)
2BYE (12787)
2DIZ (12771)
1Y7X (12750)
2JO6 (12747)
1VA9 (12742)
1EGX (12736)
2GVS (12672)
1XDX (12666)
2AJE (12305)
1KKG (12274)
1UHT (12244)
2H0P (12222)
1KVZ (12222)
2JN9 (12221)
2F05 (12221)
1Z2K (12195)
1L4S (12653)
1LL8 (12640)
1WFN (12624)
2DA0 (12617)
2H7A (12563)
1ZG2 (12539)
1RDU (12533)
1Z7P (12517)
1S6N (12186)
2APN (12184)
1TTX (12170)
2GMG (12151)
1T0G (12151)
1RQ8 (12146)
1J26 (12139)
1IYR (12127)
2JQO (12507)
1WQU (12490)
1V5Q (12475)
1TVM (12464)
1TI3 (12442)
1V9V (12410)
1WI0 (12392)
1KVN (12390)
2HGC (12091)
1WH8 (12085)
1YHD (12076)
1RJV (12054)
1X9A (12038)
1WGQ (12004)
2DMC (12001)
2COC (11995)
CSSO index (^{13}C)
Number of points (^{1}H)
100
1000

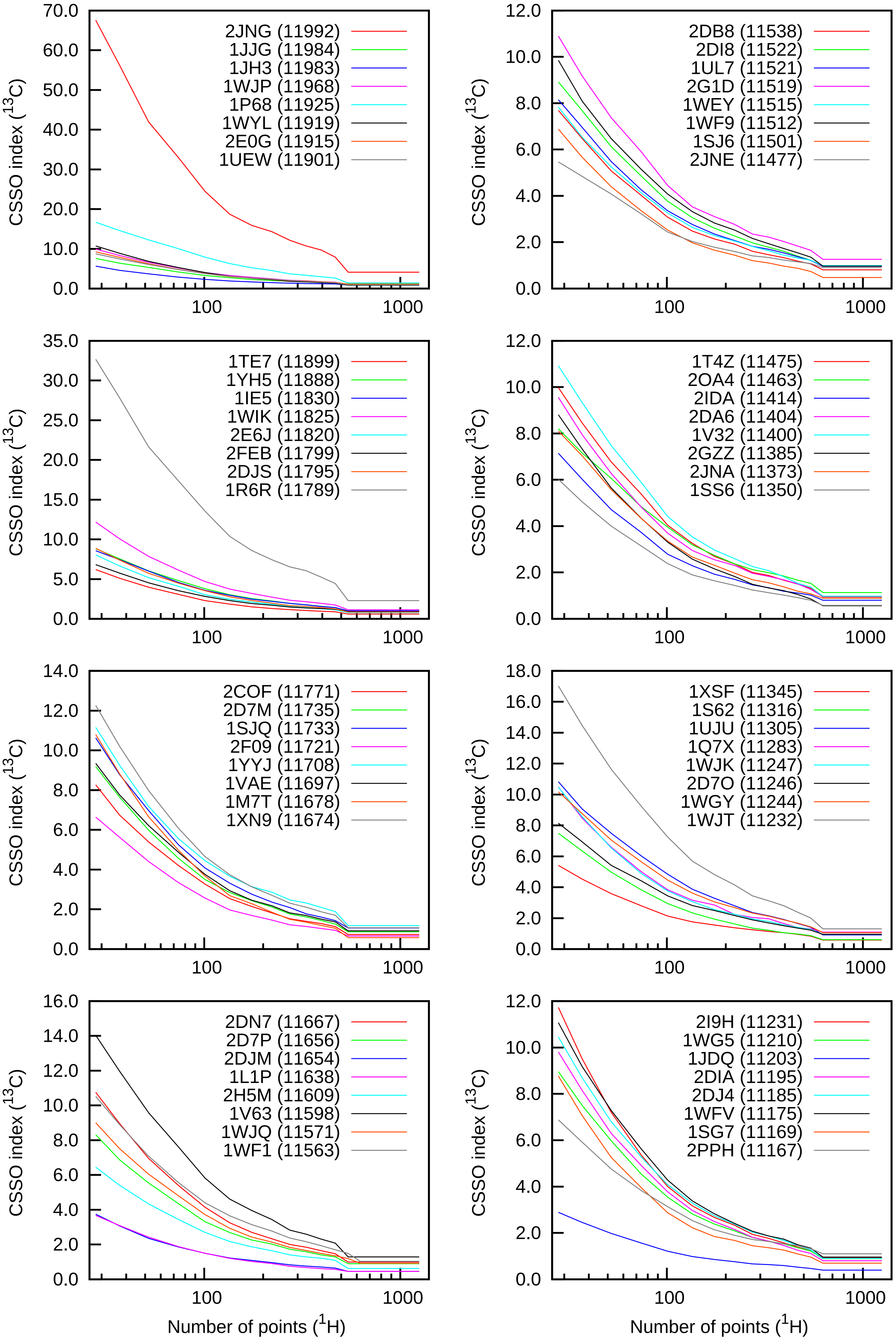

2JNG (11992)
1JJG (11984)
1JH3 (11983)
1WJP (11968)
1P68 (11925)
1WYL (11919)
2E0G (11915)
1UEW (11901)
2DB8 (11538)
2DI8 (11522)
1UL7 (11521)
2G1D (11519)
1WEY (11515)
1WF9 (11512)
1SJ6 (11501)
2JNE (11477)
1TE7 (11899)
1YH5 (11888)
1IE5 (11830)
1WIK (11825)
2E6J (11820)
2FEB (11799)
2DJS (11795)
1R6R (11789)
1T4Z (11475)
2OA4 (11463)
2IDA (11414)
2DA6 (11404)
1V32 (11400)
2GZZ (11385)
2JNA (11373)
1SS6 (11350)
2COF (11771)
2D7M (11735)
1SJQ (11733)
2F09 (11721)
1YYJ (11708)
1VAE (11697)
1M7T (11678)
1XN9 (11674)
1XSF (11345)
1S62 (11316)
1UJU (11305)
1Q7X (11283)
1WJK (11247)
2D7O (11246)
1WGY (11244)
1WJT (11232)
2DN7 (11667)
2D7P (11656)
2DJM (11654)
1L1P (11638)
2H5M (11609)
1V63 (11598)
1WJQ (11571)
1WF1 (11563)
2I9H (11231)
1WG5 (11210)
1JDQ (11203)
2DIA (11195)
2DJ4 (11185)
1WFV (11175)
1SG7 (11169)
2PPH (11167)
CSSO index (^{13}C)
Number of points (^{1}H)
100
1000

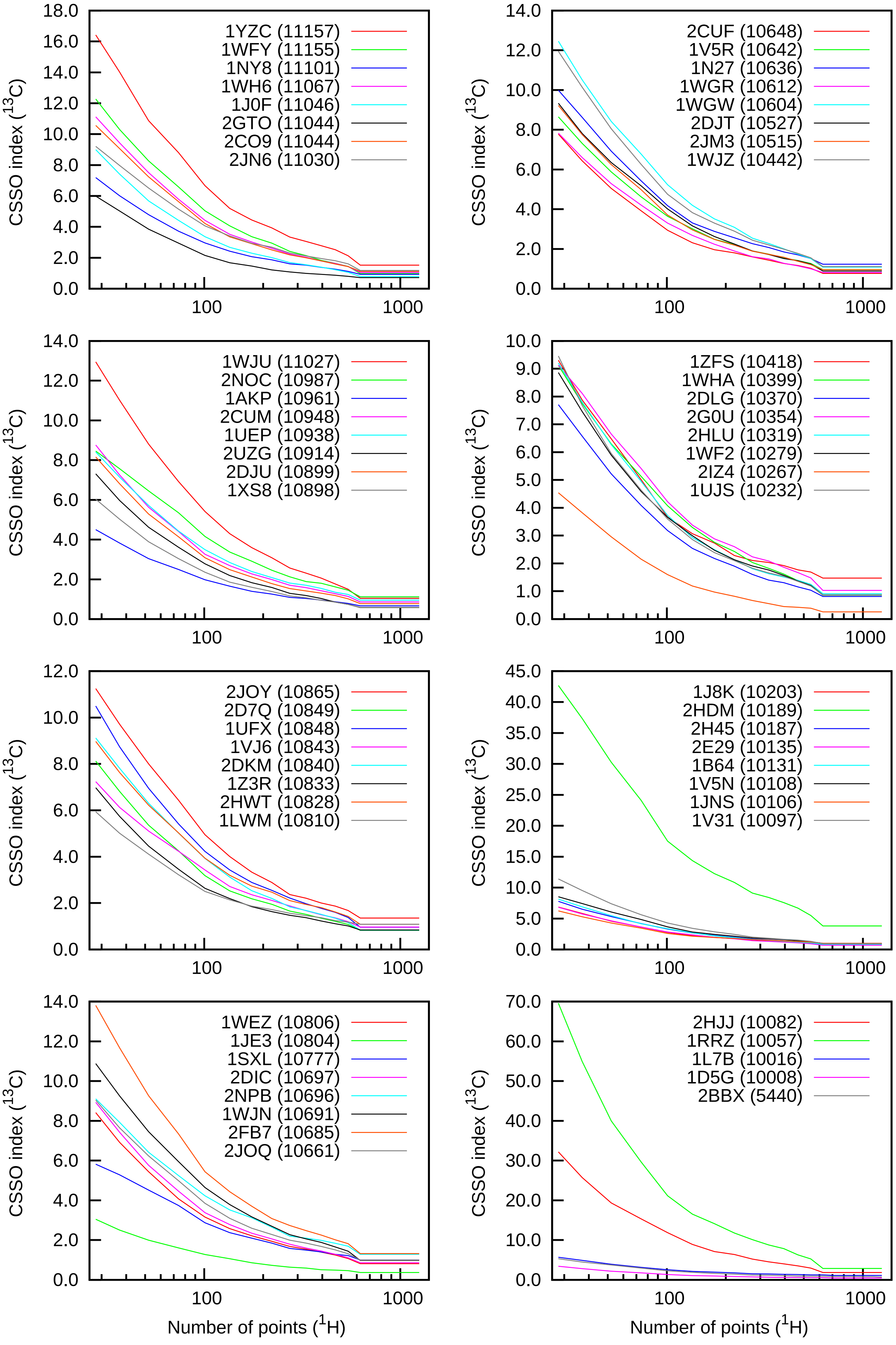
1YZC (11157)
1WFY (11155)
1NY8 (11101)
1WH6 (11067)
1J0F (11046)
2GTO (11044)
2CO9 (11044)
2JN6 (11030)
2CUF (10648)
1V5R (10642)
1N27 (10636)
1WGR (10612)
1WGW (10604)
2DJT (10527)
2JM3 (10515)
1WJZ (10442)
1WJU (11027)
2NOC (10987)
1AKP (10961)
2CUM (10948)
1UEP (10938)
2UZG (10914)
2DJU (10899)
1XS8 (10898)
1ZFS (10418)
1WHA (10399)
2DLG (10370)
2G0U (10354)
2HLU (10319)
1WF2 (10279)
2IZ4 (10267)
1UJS (10232)
2JOY (10865)
2D7Q (10849)
1UFX (10848)
1VJ6 (10843)
2DKM (10840)
1Z3R (10833)
2HWT (10828)
1LWM (10810)
1J8K (10203)
2HDM (10189)
2H45 (10187)
2E29 (10135)
1B64 (10131)
1V5N (10108)
1JNS (10106)
1V31 (10097)
1WEZ (10806)
1JE3 (10804)
1SXL (10777)
2DIC (10697)
2NPB (10696)
1WJN (10691)
2FB7 (10685)
2JOQ (10661)
2HJJ (10082)
1RRZ (10057)
1L7B (10016)
1D5G (10008)
2BBX (5440)
CSSO index (^{13}C)
Number of points (^{1}H)
100
1000
