# Supplementary material for: Effects of NMR Spectral Resolution on Protein Structure Calculation
Source: PLoS One. 2013 Jul 16;8(7):e68567. doi: 10.1371/journal.pone.0068567 (PMC3713035; doi:10.1371/journal.pone.0068567)
Supplement: Table S1 — Parameters for simulation and preparation of 13C- and 15N-resolved NOESY peak lists obtained from experimental data. (PDF) [file pone.0068567.s006.pdf]

Supporting Information Table S1

Parameters for simulation and preparation of ^{13}C- and ^{15}N-resolved NOESY spectra obtained from the experimental data

**NMR Spectra**	^{13}C-resolved NOESY	^{15}N-resolved NOESY
Number of transients	8	8
Inter-scan delay (D1), sec	1.0	1.0
Time domain sizes, complex points		
^{13}C, indirect	64	-
^{15}N, indirect	-	64
^{1}H, indirect (points studied)	28, 37, 52, 74, 101, 135, 175, 221, 273, 331, 395, 466, 542, 625, 714, 809, 910, 1017, 1130, 1250	28, 37, 52, 74, 101, 135, 175, 221, 273, 331, 395, 466, 542, 625, 714, 809, 910, 1017, 1130, 1250
^{1}H, direct detection	1024	1024
Spectral Width, Hz		
^{13}C, indirect	10752.6881	-
^{15}N, indirect	-	2000.0000
^{1}H, indirect	6596.3058	7194.2446
^{1}H, direct detection	6602.1126	7211.5384
Spectrometer Carrier Position, MHz		
^{13}C, indirect	150.8458	-
^{15}N, indirect	-	60.7924
^{1}H, indirect	599.8828	599.8828
^{1}H, direct detection	599.8828	599.8828
Number of FIDs	4320	4320
NUS sampling bias, T_2, sec		
^{13}C indirect	0.1	-
^{15}N indirect	-	0.1
^{1}H, direct detection	1.0	1.0
Calculated T_2 (^{1}H direct), sec		
PDB ID (Mw),(Da)		
2BBX (5440)	0.061275	0.061275
1D5G (10008)	0.033307	0.033307
1XKE (15117)	0.022050	0.022050
1JBJ (20602)	0.016180	0.016180
1TTE (24154)	0.013800	0.013800
2JT2 (31267)	0.010661	0.010661
