# Supplementary material for: Effects of NMR Spectral Resolution on Protein Structure Calculation
Source: PLoS One. 2013 Jul 16;8(7):e68567. doi: 10.1371/journal.pone.0068567 (PMC3713035; doi:10.1371/journal.pone.0068567)
Supplement: Table S2 — Peak counts for 13C- and 15N-resolved NOESY peak lists, molecular weight, and various RMSD values for 381 calculated protein structures. (PDF) [file pone.0068567.s007.pdf]

Supporting Information Table S2

Peak counts for ^{13}C- and ^{15}N-resolved NOESY peak lists, molecular weight, and various RMSD values for 381 calculated protein structures

	NOESY peak lists peak counts			Root Mean Square Deviation				
PDB ID	^{13}C-resolved	^{15}N-resolved	Molecular Weight*	Mean	Median	Min	Max	Final†
2LQN	5075	1843	33777	3.81700	3.79000	3.33000	4.70000	3.33000
2JT2	11332	4018	31267	1.87450	1.45000	1.38000	6.45000	1.41000
2I3E	3225	1980	24570	2.99920	2.39211	2.06591	8.80732	2.38305
2ILX	3412	1956	24225	5.04752	4.33099	3.67653	16.52510	4.33099
2JMU	7328	2387	24206	3.52006	3.42131	2.88354	4.75646	3.10607
1TTE	6208	1627	24154	7.80849	6.57142	4.62368	13.09020	5.88708
1ZC1	5365	1742	23222	2.38600	2.25500	1.94000	3.86000	2.14000
1S6L	4640	1474	23032	6.44235	6.19972	5.57528	8.84017	6.19972
1JCU	2962	1770	22610	4.44142	3.65022	3.35400	10.55890	3.65022
2HHI	8914	2565	22355	3.35905	2.86662	2.72471	9.77299	2.85716
1SOU	5827	1496	22327	3.43100	3.03000	2.87000	7.35000	2.91000
2FYH	10559	2314	21824	1.60350	1.28000	1.21000	4.22000	1.23000
2GZO	4676	1094	21764	6.31354	6.25167	4.88583	8.02083	5.90333
1Q56	1311	1163	21306	4.73511	4.62534	3.79034	6.26719	4.74730
2DO8	5728	1473	21232	2.33141	2.29765	2.01765	2.75882	2.28118
1P7M	6311	2029	21114	2.46354	2.22847	2.06232	4.37876	2.19915
2DHS	6912	2306	21081	6.82446	6.50953	4.45047	8.83610	6.23391
1YZB	9622	2583	21025	1.09058	1.01221	0.88224	2.00866	1.01615
1JBJ	4877	1621	20602	2.61972	2.35693	2.00380	7.35821	2.64436
2I3B	7022	1564	20591	2.59009	2.51210	1.94269	3.99065	2.36376
2AVX	6324	1758	20365	1.82750	1.60000	1.53000	4.95000	1.54000
1JAJ	6963	2098	20334	2.17100	1.83000	1.66000	6.13000	1.76000
1TVI	6640	1848	20115	1.39400	1.16457	1.09915	3.48936	1.11660
2JRF	5300	1518	20042	2.08500	1.99000	1.70000	3.05000	1.97000
2IN2	813	800	20000	8.94109	7.04149	5.91893	16.53220	6.25910
1R6H	4447	1537	19391	1.55579	1.37469	1.30431	3.38747	1.30432
1XWN	3707	1326	19306	2.42781	2.12792	1.99610	5.10325	2.07143
2G2K	3307	1126	19252	2.15334	1.85537	1.72119	5.01552	1.84149
1WWY	10880	2765	19055	0.74091	0.68141	0.58544	1.32443	0.67181
1YYC	3349	1354	18831	2.75450	2.67000	2.47000	3.76000	2.67000
1TVJ	9667	2485	18662	1.27300	1.09000	0.95000	3.77000	1.07000
2I9Y	5439	1485	18644	1.38084	1.37416	1.17000	1.93953	1.28779
2ADZ	5475	1443	18615	0.57044	0.49333	0.45778	1.72000	0.47111
1Q8X	6821	1857	18504	1.77200	1.62500	1.43000	3.01000	1.62000
1XPN	4474	1242	18381	2.14650	1.94500	1.68000	4.00000	1.87000
2HH8	10297	2878	18126	2.98367	3.03064	2.35286	3.71809	3.12746
2BAF	907	504	18122	2.85800	2.98000	2.10000	3.95000	3.26000
1K42	7770	2247	18082	1.68250	1.74500	1.21000	2.11000	1.74000
2HO9	6007	1694	18072	1.86950	1.65000	1.58000	4.20000	1.62000
1XOY	4492	1506	17893	2.55750	2.47500	2.35000	3.55000	2.46000

1WYO	8194	2046	17893	0.80432	0.78581	0.63226	1.15613	0.80387
2OWI	5390	1558	17759	1.81400	1.73000	1.45000	2.90000	1.77000
1SJR	3990	874	17742	2.12200	1.97000	1.74000	3.88000	2.02000
2JNU	6872	2339	17695	2.86950	2.92500	2.24000	3.86000	2.99000
1X5B	7098	1851	17664	1.03600	1.02000	0.82000	1.74000	1.05000
1PN5	1901	426	17653	3.20720	3.14293	3.02683	3.81463	3.14293
1H2O	6092	1921	17585	1.76105	1.60849	1.55000	3.02201	1.63774
2D3J	4019	2032	17491	1.65518	1.49116	1.44456	3.15007	1.45388
1RW2	6450	1833	17324	1.53200	1.47000	1.32000	2.64000	1.52000
1JAS	6390	1559	17307	1.88950	1.87500	1.51000	2.88000	1.76000
1XSC	8484	2085	17291	1.22550	1.16371	1.02839	1.84930	1.19077
1WJ1	7732	1945	17203	0.85991	0.83248	0.69293	1.52060	0.85654
1WFS	8938	2181	17155	0.87921	0.80639	0.68421	1.89624	0.74286
1X1F	7572	1518	17050	0.87994	0.85936	0.76984	1.41436	0.85936
2HF6	6691	1836	17024	1.58950	1.52500	1.38000	2.49000	1.52000
1NI7	6085	1743	17007	1.46887	1.31465	1.16135	3.05667	1.32858
1XN5	7498	1839	16981	0.68900	0.68258	0.60227	0.92349	0.69864
2G2B	3685	1313	16966	2.17500	2.04500	1.93000	4.09000	1.93000
2DKQ	6610	1484	16962	1.27600	1.24500	0.98000	1.95000	1.30000
2AGM	3560	1354	16942	2.70500	2.43000	2.11000	5.65000	2.40000
1TMW	4689	1640	16879	2.25545	2.21660	1.91953	3.15352	2.22117
1ZGG	6341	1923	16787	1.33950	1.21500	1.19000	2.84000	1.21000
1T17	5785	1743	16667	1.76770	1.51941	1.36124	4.63972	1.45710
1XO8	4933	1536	16536	1.69407	1.64910	1.51852	2.06984	1.61525
2AXL	3380	1431	16411	2.23850	2.16500	1.96000	3.41000	2.18000
1WK1	7020	1860	16401	0.93842	0.92827	0.81223	1.12165	0.91860
1P6T	4586	1666	16376	1.75011	1.43804	1.34022	4.64674	1.37935
1UFG	6448	1697	16267	1.04659	1.03212	0.95496	1.23469	1.03212
2GBS	6532	1721	16263	1.24647	1.18529	1.08015	1.88309	1.18529
2BW2	6555	1777	16168	1.93100	1.90000	1.38000	3.06000	2.74000
1JW3	4686	1537	16164	2.32950	2.28000	2.10000	3.42000	2.30000
1XN6	7183	1783	16074	0.78935	0.76015	0.63964	1.19584	0.76015
2BL5	3477	1456	16070	3.74891	3.92099	2.74182	4.85091	3.00066
1VYN	13801	3713	16053	2.29900	1.98500	1.65000	7.55000	1.96000
1WLM	6621	1694	15928	0.85852	0.81069	0.75604	1.43010	0.81069
2CH0	3249	879	15897	3.17850	3.23500	2.82000	3.66000	3.34000
1WYN	7023	1808	15796	0.72350	0.71000	0.59000	1.02000	0.67000
1UJO	7274	1799	15783	0.75671	0.69118	0.67323	1.48110	0.67323
1TWO	6395	1963	15775	3.01450	2.46000	1.89000	12.83000	1.89000
1ZTS	6393	1523	15746	3.87120	4.00400	2.59200	4.59200	4.24800
2DMM	7024	1431	15675	1.42650	1.36000	1.29000	1.98000	1.29000
1WPI	3391	1091	15656	2.17200	1.99500	1.85000	4.23000	1.88000
2GJY	2630	1195	15654	1.91549	1.85865	1.74135	2.49925	1.77744
1VDY	6306	1535	15562	1.37200	1.23934	1.19344	3.37836	1.24853
1WJJ	6757	1462	15526	0.65047	0.61134	0.52983	1.27160	0.60319
2DJ0	6262	1437	15512	2.04423	2.02960	1.79360	2.68096	2.02960

1WIN	6333	1624	15455	0.81162	0.71797	0.60649	2.05135	0.71351
2JOZ	4848	1247	15418	2.00000	1.80500	1.60000	3.47000	1.78000
1U6F	3423	1175	15416	1.41850	1.37000	1.29000	2.19000	1.37000
2FE0	5316	1636	15397	0.97150	0.92000	0.86000	1.47000	0.91000
1GXE	1906	1018	15367	0.91407	0.85422	0.80008	1.31141	0.80008
2JOE	5374	1515	15364	2.11202	1.63976	1.39614	6.86827	1.61165
1NXI	4728	1257	15299	1.16399	1.13041	0.91757	2.31757	1.13514
2EXN	5374	1485	15272	1.20844	1.21545	1.03781	1.37439	1.27155
2ETT	3138	915	15199	3.23355	2.95815	2.60426	7.15037	3.01259
1UKX	6097	1399	15128	1.04073	0.99567	0.94417	1.72525	0.99567
1XKE	3000	1394	15117	2.35400	1.89000	1.83000	8.32000	1.83000
2FVT	4219	1278	15112	1.84400	1.76500	1.55000	2.93000	1.78000
2DJ1	6344	1515	15104	1.49800	1.41000	1.31000	1.95000	1.35000
1TQZ	2360	1082	15082	6.19934	6.09058	4.12826	9.28397	5.24826
2BGO	6920	1884	14934	1.03850	1.07000	0.86000	1.21000	1.07000
1PUN	3802	1473	14921	1.60697	1.35866	1.31181	4.16969	1.35866
1G03	2767	1185	14888	2.49750	2.22000	2.09000	4.87000	2.21000
1WLX	4647	1817	14812	1.36202	1.10090	1.01902	3.53926	1.17369
1Z9B	4208	1224	14792	1.07547	0.98763	0.84654	1.92974	1.01039
2JMG	4246	1335	14774	2.39050	1.99500	1.46000	6.50000	1.70000
1WFM	5586	1279	14736	0.70481	0.64750	0.63000	1.21625	0.63875
1WGU	6654	1598	14732	0.94005	0.90193	0.79965	1.35754	1.01351
1WFI	6053	1475	14691	1.10900	1.07500	0.93000	1.48000	1.07000
2I4K	3793	1121	14681	1.67260	1.50698	1.31302	3.20794	1.35032
1V5M	6197	1246	14672	0.78834	0.76195	0.63929	1.42726	0.77310
2JQ5	5269	1430	14637	1.51738	1.45395	1.34661	2.26387	1.46371
2DJ3	6492	1420	14630	1.03180	0.97248	0.84606	1.80881	1.06973
1V9W	6654	1623	14609	1.23819	1.23101	1.11126	1.60941	1.23580
1IEH	4643	1432	14602	1.17081	1.13896	1.02313	1.69878	1.15826
2FKI	4501	1213	14586	3.68300	3.15500	2.74000	5.81000	3.22000
2DKP	6301	1241	14585	0.64284	0.63080	0.59760	0.83000	0.60590
1WK0	6303	1438	14548	0.97950	0.85500	0.81000	1.98000	0.84000
2B1W	4033	1115	14500	1.36431	1.31011	1.26299	1.96989	1.26299
1WFJ	6527	1471	14491	0.89800	0.82000	0.80000	1.36000	0.82000
1R9P	3218	847	14462	1.84487	1.82303	1.56667	2.41172	1.82303
2GM2	3644	989	14450	1.64200	1.35500	1.26000	5.10000	1.26000
1WJR	6345	1362	14433	0.87549	0.88400	0.69018	1.32364	0.92655
1X5E	6345	1474	14387	1.31650	1.40500	1.09000	1.62000	1.41000
2G7J	6118	1619	14382	1.64706	1.63477	1.47413	2.06000	1.67257
2D9V	5420	1366	14377	0.71881	0.72936	0.56881	1.11927	0.77064
1X05	6264	1296	14337	0.87778	0.84760	0.71942	1.27346	0.93442
2D9Z	4914	1280	14321	0.59984	0.57982	0.46248	0.93876	0.59363
1UG7	6161	1414	14297	0.88597	0.86081	0.75928	1.47441	0.90937
1V5S	5322	1462	14256	1.02775	1.01895	0.90779	1.38947	1.03747
1R5S	2196	1080	14204	3.03300	2.36500	1.37000	5.70000	2.23000
2JP2	3543	1102	14195	2.52773	2.41289	2.10140	4.08193	2.48263

1VEE	5819	1604	14186	1.09800	1.03500	0.98000	1.82000	1.00000
2F1E	8081	1653	14176	5.75000	5.70500	4.94000	6.86000	5.63000
1V88	7101	1572	14158	0.66017	0.64407	0.53390	1.01695	0.66102
1X1G	5994	1202	14051	0.71643	0.64509	0.59955	1.14598	0.62991
1WJS	5504	1197	14024	1.19350	1.17000	1.01000	1.40000	1.37000
1J7H	3708	1379	14021	1.58921	1.32095	1.15810	5.30190	1.29381
2DHJ	5590	1185	14018	0.92246	0.93248	0.79376	1.07890	0.94789
1WFG	6092	1496	13993	0.94000	0.89000	0.75000	1.75000	0.99000
2DMK	5692	1373	13947	0.57249	0.55647	0.52275	0.81784	0.52275
1XHS	4830	1369	13934	1.67432	1.68225	1.34413	1.89514	1.89514
1V5P	5951	1477	13918	0.58824	0.56269	0.48125	1.11058	0.57750
2HGK	3957	979	13847	2.45631	2.30308	2.20615	4.51385	2.27077
1Q2Z	4851	1374	13846	1.96200	1.87000	1.64000	3.66000	1.89000
1YUA	5512	1155	13841	2.41650	2.43500	1.66000	3.40000	2.50000
1TQ1	2685	906	13805	2.17425	2.09625	1.91750	3.07938	2.09625
1XNE	5812	1218	13798	1.33695	1.30349	1.18899	2.13138	1.33872
2GA5	3719	1262	13770	2.01600	1.88000	1.75000	3.52000	1.78000
2DIY	6118	1362	13768	1.23800	1.22000	1.11000	1.47000	1.16000
2GW6	3815	1290	13765	1.95000	1.91000	1.81000	2.44000	1.89000
1WH4	6098	1246	13757	1.14450	1.07000	0.99000	2.41000	1.09000
2JRZ	3818	1408	13731	4.18400	4.14500	3.24000	5.52000	4.19000
1WFO	6576	1421	13725	0.68447	0.68895	0.56368	0.91263	0.71579
2COM	6282	1465	13714	1.28350	1.21000	1.16000	2.06000	1.21000
1P6Q	2256	1365	13713	2.63400	2.12000	2.05000	5.93000	2.05000
1TUJ	2005	1181	13690	3.65840	2.40545	2.17058	12.14070	2.41355
2DML	6936	1662	13661	0.74143	0.71429	0.63810	1.10476	0.81905
1ZIT	4064	1111	13641	1.52778	1.38205	1.32564	2.55727	1.37265
2D9W	5036	1087	13609	0.48541	0.48881	0.43450	0.63138	0.48881
1X5I	5227	1127	13601	0.89400	0.82000	0.79000	1.61000	0.80000
2DHI	5996	1366	13593	0.73550	0.68646	0.60708	1.13943	0.75651
2G46	3174	939	13589	2.08867	2.05111	1.89704	3.02370	2.00296
1JFN	2481	871	13573	1.88700	1.79000	1.71000	3.10000	1.71000
1Z1M	2840	870	13558	1.83300	1.77500	1.52000	2.71000	1.87000
1WGV	5683	1261	13518	0.97800	0.96500	0.87000	1.21000	0.93000
2DI7	5188	1131	13499	1.71500	1.65000	1.53000	2.51000	1.57000
1TUZ	4767	1397	13480	1.35070	1.17758	1.08091	3.04939	1.17758
2AVG	3843	1308	13469	1.77950	1.76500	1.53000	2.28000	1.78000
2DIB	5577	1411	13435	0.96150	0.87000	0.83000	1.86000	0.83000
2GQB	4835	1393	13433	0.86654	0.81184	0.72928	1.56176	0.77744
1WFZ	6596	1570	13428	0.64750	0.55000	0.51000	1.48000	0.55000
2HGA	5295	1593	13390	1.50191	1.45099	1.14457	2.18099	1.39691
1X5K	5367	1124	13379	0.92350	0.94500	0.71000	1.17000	1.04000
1UJT	5256	1294	13338	1.14200	1.05000	1.00000	1.89000	1.00000
2DHK	5835	1242	13297	0.76760	0.75893	0.63316	1.20561	0.80663
2D9X	5140	1355	13288	0.94100	0.91000	0.85000	1.12000	1.01000
1WJO	5868	1478	13242	0.75850	0.75500	0.63000	1.14000	0.77000

1X5C	6524	1300	13233	1.18643	1.14551	1.12626	1.63645	1.14551
2JMX	5099	1381	13230	1.90550	1.66000	1.45000	4.60000	1.74000
1UW0	1866	777	13226	0.00000	0.00000	0.00000	0.00000	2.79792
1P6S	2357	918	13217	2.37906	2.33398	2.15704	2.91537	2.15704
2DI9	5615	1263	13186	1.54750	1.60500	0.93000	1.78000	1.60000
1S04	6342	1458	13156	1.17100	1.10000	0.96000	2.49000	1.11000
2DJ2	6569	1393	13136	1.29650	1.22500	1.17000	2.04000	1.23000
2A7O	6775	1687	13127	2.28850	2.16500	1.70000	3.98000	2.15000
1V5K	6987	1600	13107	0.77350	0.71000	0.68000	1.27000	0.69000
1WF5	5711	1301	13047	0.80300	0.75500	0.72000	1.25000	0.75000
1V5U	5509	1225	13044	0.73332	0.73080	0.59640	1.04160	0.73080
1WFT	4347	883	13022	1.04725	1.06617	0.86841	1.32411	1.08336
2HG7	2685	706	13012	1.59400	1.42000	1.26000	2.71000	1.42000
2DN6	6005	1268	13006	0.83324	0.82667	0.74212	1.02394	0.80788
2DBJ	5018	1123	13000	0.81622	0.78644	0.73525	1.25644	0.77248
1M3V	2666	884	13000	3.85161	3.61413	3.14184	4.95058	3.49689
1A5J	4294	1263	12941	6.45549	6.42634	5.20119	7.56040	5.20119
2HJQ	4344	1222	12916	5.55206	5.38087	3.76888	7.43786	5.28551
2D9Y	5324	1187	12905	0.55987	0.51687	0.49515	0.92081	0.50384
2GS0	5582	1586	12886	0.98838	0.92829	0.88685	1.34270	0.89514
2COD	4469	929	12861	0.62271	0.63742	0.49032	0.76000	0.63742
2JN7	2383	854	12804	4.83100	3.92000	2.82000	9.66000	3.92000
2BYE	3449	860	12787	0.80794	0.74530	0.63800	1.91980	0.73080
2DIZ	5870	1366	12771	1.18150	1.13500	1.02000	1.60000	1.13000
1Y7X	1688	930	12750	1.83944	1.73717	1.62509	2.38160	1.74651
2JO6	4831	1280	12747	1.42800	1.40500	1.24000	2.07000	1.37000
1VA9	5574	1433	12742	0.77135	0.71882	0.66353	1.17039	0.71882
1EGX	1809	1084	12736	1.82300	1.69500	1.62000	2.66000	1.69000
2GVS	4487	1486	12672	1.20100	1.01000	0.87000	3.96000	0.98000
1XDX	1361	897	12666	4.74550	4.31000	3.82000	8.25000	4.04000
1L4S	4900	1353	12653	0.99292	0.94777	0.86729	1.70776	0.93882
1LL8	5011	1365	12640	0.86946	0.81726	0.74452	1.38635	0.85577
1WFN	5352	1210	12624	0.79250	0.71000	0.67000	1.38000	0.71000
2DA0	4957	1146	12617	0.75643	0.71571	0.60857	1.76571	0.65143
2H7A	3319	994	12563	1.12125	1.16007	0.83056	1.28194	1.19167
1ZG2	3834	945	12539	1.00269	1.00481	0.92372	1.07180	1.03654
1RDU	4888	1249	12533	1.11235	1.00290	0.97084	2.45458	0.98916
1Z7P	5221	1494	12517	1.31750	1.28000	1.20000	1.73000	1.29000
2JQO	4316	1161	12507	2.48000	2.42000	2.28000	3.00000	2.33000
1WQU	5097	1131	12490	1.02700	1.01000	0.86000	1.27000	1.01000
1V5Q	4528	1102	12475	0.79906	0.71532	0.68043	1.40447	0.69787
1TVM	2566	941	12464	3.00266	2.62161	2.15129	5.90516	2.41258
1TI3	3972	1095	12442	1.71600	1.64000	1.54000	2.27000	1.64000
1V9V	5689	1192	12410	0.76548	0.62176	0.52264	2.09055	0.73890
1WI0	5702	1246	12392	0.94000	0.91500	0.81000	1.35000	0.93000
1KVN	4188	1043	12390	2.03950	1.79500	1.65000	4.88000	1.74000

2DS4	5423	1328	12371	1.41800	1.17500	0.98000	2.60000	1.07000
1UEM	4833	1095	12354	1.20200	1.20500	1.02000	1.82000	1.22000
1RJJ	3657	1191	12346	1.43393	1.37682	1.21302	2.41719	1.34583
2DMB	4025	988	12335	1.14650	1.12000	0.83000	1.55000	1.08000
2JMP	3094	995	12327	2.45450	2.19000	1.86000	4.30000	2.19000
1UC6	3925	1006	12318	1.86600	1.87500	1.55000	2.26000	1.93000
1UJX	6071	1495	12315	0.62569	0.64776	0.00000	0.85429	0.65714
1X6D	4873	1282	12306	0.82750	0.83000	0.69000	1.32000	0.86000
2AJE	3065	1032	12305	1.25773	1.07709	0.94987	4.79177	1.11949
1KKG	4494	1438	12274	1.39900	1.10000	1.02000	3.95000	1.06000
1UHT	5762	1321	12244	0.64451	0.60117	0.54990	1.15573	0.57786
2H0P	3933	886	12222	2.08850	2.03500	1.97000	2.56000	2.01000
1KVZ	4609	1252	12222	1.53600	1.49000	1.34000	2.18000	1.52000
2JN9	3718	985	12221	2.09546	1.92522	1.63109	5.45478	1.63109
2F05	3114	1037	12221	0.79462	0.76923	0.70769	1.18461	0.70769
1Z2K	3552	933	12195	1.31027	1.34976	0.00000	1.66806	1.34573
1S6N	4184	1200	12186	3.30650	3.30500	2.94000	3.81000	3.39000
2APN	4505	1658	12184	0.77907	0.72544	0.67913	1.11130	0.71000
1TTX	4105	1305	12170	1.48250	1.56000	1.22000	2.07000	1.58000
2GMG	3656	1015	12151	2.00061	1.72000	1.51608	3.99856	1.58701
1T0G	3332	1185	12151	1.72450	1.68000	1.53000	2.29000	1.55000
1RQ8	4693	1235	12146	1.90500	1.75500	1.56000	3.33000	1.70000
1J26	4907	1205	12139	0.95078	0.93739	0.79511	1.52326	0.93739
1IYR	404	537	12127	6.50550	6.16000	5.23000	8.23000	6.02000
2HGC	4337	1153	12091	1.77722	1.76957	1.53043	2.46783	1.76957
1WH8	5418	1143	12085	1.00300	0.97000	0.82000	1.67000	0.98000
1YHD	4061	1203	12076	6.33522	6.24165	5.44094	7.35106	7.03271
1RJV	2784	1119	12054	2.31150	1.50500	1.36000	9.46000	1.36000
1X9A	3346	992	12038	2.45550	1.91500	1.72000	6.74000	2.06000
1WGQ	5067	1242	12004	0.78580	0.78670	0.64202	1.10319	0.79575
2DMC	4951	1039	12001	1.22100	1.16000	0.95000	2.16000	1.32000
2COC	5210	1165	11995	0.56174	0.54949	0.48186	0.73546	0.53258
2JNG	16817	4517	11992	1.93904	1.87770	1.29238	2.57540	2.57540
1JJG	2809	721	11984	0.88941	0.83294	0.74824	1.39059	0.75529
1JH3	3497	1151	11983	1.46000	1.43500	1.25000	2.19000	1.44000
1WJP	4560	1208	11968	2.72994	2.47369	1.80713	6.01387	2.51813
1P68	5209	1574	11925	1.25450	1.11000	0.96000	2.89000	1.01000
1WYL	5443	1290	11919	0.74800	0.73500	0.61000	1.30000	0.76000
2E0G	4135	1195	11915	4.28841	4.20577	2.69135	8.16058	2.69135
1UEW	4300	971	11901	0.76464	0.76000	0.70089	0.92889	0.76844
1TE7	3917	1237	11899	1.74003	1.51125	1.41360	4.92900	1.41360
1YH5	3926	1074	11888	1.87238	1.83810	1.62857	2.63809	1.80952
1IE5	4066	1164	11830	2.23700	2.19000	2.03000	2.67000	2.30000
1WIK	4849	1061	11825	1.98804	2.05931	1.65670	2.23979	2.09170
2E6J	5091	1139	11820	1.66500	1.72000	1.40000	2.16000	1.76000
2FEB	3084	847	11799	3.16850	2.95500	2.78000	4.72000	2.84000

2DJS	4849	1176	11795	1.00200	0.91000	0.75000	1.83000	1.08000
1R6R	8082	2712	11789	2.33150	2.31000	1.32000	3.29000	2.83000
2COF	4657	1091	11771	0.80049	0.77006	0.66400	1.30033	0.85767
2D7M	5832	1354	11735	1.12850	1.07500	0.90000	2.02000	1.09000
1SJQ	4531	1231	11733	1.21000	1.21000	1.07000	1.70000	1.22000
2F09	2853	876	11721	1.61000	1.58000	1.51000	1.94000	1.54000
1YYJ	4371	1626	11708	2.54750	2.25000	1.95000	7.13000	1.95000
1VAE	4539	1054	11697	1.32450	1.31000	1.18000	1.60000	1.31000
1M7T	4790	1396	11678	1.44350	1.40500	1.26000	1.68000	1.58000
1XN9	5870	1441	11674	0.80135	0.64052	0.60779	2.08520	0.63584
2DN7	5325	1222	11667	0.84277	0.86769	0.62769	1.35692	0.90462
2D7P	4873	1173	11656	0.98061	0.93725	0.88596	1.24034	0.92326
2DJM	2104	1193	11654	2.31550	2.23000	2.08000	3.26000	2.21000
1L1P	1846	1065	11638	2.34550	2.08500	1.92000	4.57000	2.02000
2H5M	3909	1201	11609	1.33733	1.17972	1.04449	3.54382	1.16573
1V63	5252	1277	11598	3.84500	4.09000	2.73000	4.37000	4.25000
1WJQ	4599	901	11571	0.88670	0.86565	0.70783	1.31044	0.86087
1WF1	4670	1206	11563	0.73800	0.76000	0.60000	0.96000	0.79000
2DB8	4056	970	11538	0.94484	0.94484	0.74105	1.33390	0.98190
2DI8	4876	1167	11522	1.04850	1.01000	0.95000	1.69000	0.99000
1UL7	4275	1013	11521	1.00250	1.00000	0.87000	1.46000	1.00000
2G1D	3864	1089	11519	1.76303	1.64324	1.53946	2.89730	1.64324
1WEY	4644	927	11515	0.94267	0.94987	0.77716	1.12257	0.94987
1WF9	5259	1394	11512	0.78350	0.72500	0.68000	1.28000	0.71000
1SJ6	3305	1125	11501	2.06600	2.08500	1.76000	2.73000	2.11000
2JNE	2674	658	11477	2.33100	2.02500	1.90000	3.94000	1.90000
1T4Z	4708	1216	11475	2.03150	2.02000	1.86000	2.27000	2.04000
2OA4	3690	955	11463	2.02150	1.94500	1.74000	2.82000	2.02000
2IDA	4176	1106	11414	1.29650	1.27500	1.21000	1.67000	1.21000
2DA6	4919	1296	11404	0.73200	0.70500	0.58000	1.39000	0.75000
1V32	5086	1121	11400	1.35800	1.31000	1.15000	1.87000	1.30000
2GZZ	4579	1348	11385	1.49200	1.43000	1.38000	2.05000	1.41000
2JNA	2524	708	11373	1.67450	1.56000	1.51000	2.98000	1.51000
1SS6	2911	1015	11350	1.31911	1.25333	1.19111	2.32889	1.24444
1XSF	2322	1441	11345	1.23750	1.20000	1.12000	1.66000	1.14000
1S62	3051	880	11316	1.84483	1.70759	1.59435	2.88071	1.63965
1UJU	4911	1320	11305	0.63862	0.60963	0.56274	1.01463	0.61390
1Q7X	3378	1575	11283	1.01957	0.86522	0.80870	2.79130	0.80870
1WJK	5787	1144	11247	1.26091	1.22299	1.15662	1.69701	1.23247
2D7O	4671	1129	11246	0.77513	0.76212	0.66918	0.92941	0.76212
1WGY	4267	850	11244	1.02558	0.97535	0.74512	1.82512	1.04651
1WJT	5180	1159	11232	0.77280	0.73920	0.63360	1.68000	0.74880
2I9H	4945	1348	11231	1.20650	1.16000	1.09000	1.77000	1.17000
1WG5	5121	1108	11210	0.94650	0.92500	0.79000	1.24000	0.90000
1JDQ	1044	598	11203	1.95286	1.56108	1.39243	6.52108	1.55676
2DIA	4702	1046	11195	1.30784	1.29316	1.15579	1.57263	1.29790

2DJ4	5521	1346	11185	1.47900	1.53000	1.15000	2.19000	1.61000
1WFV	4985	1272	11175	0.68267	0.63825	0.60375	1.06088	0.62963
1SG7	4031	1005	11169	1.30159	1.29087	1.03627	1.57227	1.22387
2PPH	3083	976	11167	2.10050	2.00000	1.84000	2.98000	1.90000
1YZC	3674	1315	11157	1.89450	1.68000	1.38000	5.89000	1.86000
1WFY	4928	937	11155	0.66808	0.59747	0.50973	1.54589	0.61836
1NY8	4036	1199	11101	1.11949	1.06256	0.99615	1.64128	1.06256
1WH6	4607	1149	11067	0.96500	0.92500	0.85000	1.65000	0.93000
1J0F	5027	1306	11046	1.43400	1.40000	1.34000	1.73000	1.40000
2GTO	2759	768	11044	1.22306	1.19944	1.12389	1.63389	1.15222
2CO9	3938	971	11044	1.10350	0.87000	0.81000	2.99000	0.86000
2JN6	3387	1107	11030	3.93791	4.16045	2.30727	6.17727	4.18091
1WJU	4857	1002	11027	0.89700	0.84434	0.70816	1.74316	0.84434
2NOC	2701	673	10987	2.12000	2.06000	1.85000	2.79000	2.02000
1AKP	3199	1104	10961	1.90069	1.79071	1.63500	3.27000	1.63500
2CUM	4346	860	10948	0.84378	0.84000	0.65124	1.58562	0.85888
1UEP	4170	876	10938	1.00500	0.99000	0.89000	1.39000	1.01000
2UZG	2209	697	10914	1.82123	1.66464	1.34488	3.55500	1.47655
2DJU	4282	972	10899	0.63378	0.58641	0.54130	1.03750	0.58641
1XS8	2514	966	10898	2.18650	2.08500	1.93000	3.22000	1.99000
2JOY	3540	824	10865	4.96753	4.84706	4.53647	6.20235	4.80941
2D7Q	4842	1157	10849	0.96900	0.96000	0.90000	1.13000	0.97000
1UFX	4972	961	10848	0.72926	0.66648	0.60852	1.53580	0.61818
1VJ6	2993	851	10843	0.95972	0.91023	0.86605	1.43163	0.87488
2DKM	4098	808	10840	0.83958	0.80264	0.72055	1.29516	0.75703
1Z3R	4186	1034	10833	1.48953	1.51826	1.36979	1.77211	1.53263
2HWT	4271	953	10828	2.12550	2.12000	1.98000	2.41000	2.12000
1LWM	2833	883	10810	1.30000	1.21000	1.02000	2.88000	1.05000
1WEZ	3986	903	10806	1.14369	1.14461	0.99692	1.37539	1.05231
1JE3	665	296	10804	3.37400	3.15000	3.02000	5.04000	3.15000
1SXL	2562	732	10777	3.56442	3.49640	3.27419	3.97256	3.66419
2DIC	4948	1235	10697	1.06053	1.01966	0.76685	1.89607	1.16292
2NPB	3775	1216	10696	1.84966	1.74253	1.25977	2.55632	2.11494
1WJN	4830	1054	10691	0.82638	0.78375	0.62333	1.48500	0.79750
2FB7	3766	854	10685	1.16348	1.08870	0.97391	1.53043	1.23130
2JOQ	4299	1000	10661	1.24850	1.24000	1.10000	1.57000	1.27000
2CUF	4118	1035	10648	0.68450	0.63000	0.58000	1.10000	0.61000
1V5R	3974	759	10642	1.51900	1.54500	1.24000	1.88000	1.56000
1N27	4230	816	10636	1.03566	1.01854	0.87805	1.28195	1.09756
1WGR	3976	953	10612	0.76335	0.66952	0.65191	1.49762	0.66952
1WGW	4248	1218	10604	1.03544	0.86468	0.72494	3.94785	0.86468
2DJT	4012	997	10527	0.98100	0.97000	0.75000	1.65000	1.00000
2JM3	3997	871	10515	1.64651	1.63805	1.50451	1.91402	1.63805
1WJZ	4307	961	10442	1.17050	1.17000	0.90000	2.03000	1.21000
1ZFS	3287	1098	10418	2.90650	2.56000	1.57000	9.00000	1.57000
1WHA	3711	1068	10399	0.75839	0.70833	0.57611	1.27500	0.70833

2DLG	3879	874	10370	1.06300	0.92500	0.84000	1.94000	0.88000
2G0U	2510	878	10354	1.55657	1.48200	1.09250	3.03050	1.49150
2HLU	3826	1145	10319	1.44070	1.43736	1.31798	1.60449	1.47079
1WF2	4369	1049	10279	0.74350	0.70000	0.64000	1.14000	0.67000
2IZ4	484	497	10267	7.88950	6.93500	5.86000	14.75000	6.43000
1UJS	5473	1280	10232	0.52013	0.45867	0.42750	0.94406	0.42750
1J8K	3422	909	10203	1.80400	1.73500	1.65000	2.59000	1.83000
2HDM	7778	1915	10189	1.26046	1.22308	1.03385	1.89231	1.38462
2H45	4530	1227	10187	0.82942	0.85000	0.72474	1.06474	0.86790
2E29	3527	829	10135	0.87300	0.85000	0.78000	1.27000	0.85000
1B64	3866	1028	10131	1.17000	1.02000	0.92000	2.48000	0.92000
1V5N	3827	916	10108	0.80300	0.77500	0.73000	1.02000	0.75000
1JNS	3671	979	10106	1.59742	1.52427	1.38742	3.00135	1.45348
1V31	4801	937	10097	0.85450	0.83500	0.75000	1.22000	0.85000
2HJJ	8527	2077	10082	2.21400	2.32000	1.15000	2.54000	2.42000
1RRZ	5208	575	10057	5.63242	5.58316	4.48000	6.62737	5.83579
1L7B	2703	902	10016	1.56150	1.33000	1.20000	3.27000	1.33000
1D5G	1999	872	10008	1.96150	1.73500	1.64000	3.69000	1.71000
2BBX	1955	514	5440	0.48367	0.47667	0.42667	0.78000	0.47333

* Molecular weight specified in Dalton
† Final RMSD value calculated for 381 protein structures at the highest digital resolution (obtained by sampling 1250 points)
