# Supplementary material for: Effects of NMR Spectral Resolution on Protein Structure Calculation
Source: PLoS One. 2013 Jul 16;8(7):e68567. doi: 10.1371/journal.pone.0068567 (PMC3713035; doi:10.1371/journal.pone.0068567)
Supplement: Table S3 — Chemical shift spectral overlap indices for the 13C- and 15N-resolved NOESY peak lists at various resolutions for 381 calculated protein structures. (PDF) [file pone.0068567.s008.pdf]

Supporting Information Table S3

Chemical Shift Spectral Overlap indices for the ^{13}C- and ^{15}N-resolved NOESY peak lists obtained at three different numbers of sampled points for 381 calculated protein structures

		^{13}C-resolved NOESY peak lists CSSO index			^{15}N-resolved NOESY peak lists CSSO index		
PDB ID	Molecular Weight*	28 points	1250 points	Min RMSD†	28 points	1250 points	Min RMSD†
2LQN	33777	5.03807	0.82383	0.82383	3.19216	0.32118	0.32118
2JT2	31267	11.19140	1.43952	1.43952	9.44018	0.90030	0.90030
2I3E	24570	3.52718	0.41566	0.41566	6.91084	0.47416	0.47416
2ILX	24225	5.02584	0.59953	0.59953	5.63692	0.40103	0.40103
2JMU	24206	9.38951	0.91915	2.11144	6.80890	0.46619	1.30869
1TTE	24154	11.53180	1.41890	1.41890	6.02838	0.49599	0.49599
1ZC1	23222	7.68128	0.81844	0.81844	4.67281	0.30184	0.30184
1S6L	23032	5.60423	0.86146	0.86146	3.36785	0.26839	0.26839
1JCU	22610	5.06360	0.87212	0.87212	5.56122	0.49547	0.49547
2HHI	22355	11.70070	0.99618	3.91760	5.09730	0.36968	1.58421
1SOU	22327	6.95516	0.91840	0.91840	3.39060	0.27248	0.27248
2FYH	21824	18.16110	2.03544	2.03544	6.94194	0.49220	0.49220
2GZO	21764	6.21670	1.20642	1.20642	2.82904	0.32353	0.32353
1Q56	21306	3.59540	0.35556	0.35556	3.04754	0.23855	0.23855
2DO8	21232	6.79727	1.18210	2.11499	3.50648	0.33811	0.80300
1P7M	21114	9.13307	1.38906	2.29818	4.51804	0.41127	0.84133
2DHS	21081	10.02290	0.94382	1.64234	5.68783	0.43565	0.78435
1YZB	21025	11.43120	1.32862	3.46631	7.31626	0.64726	1.97827
1JBJ	20602	9.55779	1.12995	1.69575	4.08421	0.38019	0.63282
2I3B	20591	12.61430	1.74059	6.86345	4.40180	0.42876	2.16560
2AVX	20365	8.54922	1.10858	1.10858	5.83447	0.42237	0.42237
1JAJ	20334	9.39255	0.91965	2.20354	4.17686	0.30688	0.90440
1TVI	20115	11.72200	1.36087	1.36087	5.07926	0.39848	0.39848
2JRF	20042	11.73820	1.79562	1.79562	5.73545	0.51058	0.51058
2IN2	20000	4.89963	0.48823	0.48823	3.80856	0.28464	0.28464
1R6H	19391	5.33619	0.57600	0.57600	5.56891	0.43762	0.43762
1XWN	19306	5.17968	0.55499	0.55499	3.25758	0.17424	0.17424
2G2K	19252	9.74614	1.37352	1.37352	2.96429	0.23571	0.23571
1WWY	19055	12.79930	1.29115	2.86592	7.48967	0.57630	1.46285
1YYC	18831	4.64972	0.41161	0.41161	3.65579	0.19733	0.19733
1TVJ	18662	13.67040	1.41517	3.49136	8.40177	0.48487	1.63292
2I9Y	18644	10.13360	1.17062	3.38966	4.15010	0.30020	1.14402
2ADZ	18615	12.30650	1.54800	6.89267	5.62561	0.45233	2.67641
1Q8X	18504	8.14908	1.05825	1.96390	4.10589	0.24960	0.68287
1XPN	18381	5.63876	0.76858	0.76858	2.67799	0.17799	0.17799
2HH8	18126	28.13860	2.04276	23.56020	15.77720	0.86490	13.05920
2BAF	18122	4.95450	0.67037	0.67037	2.61446	0.38153	0.38153
1K42	18082	6.22798	0.67697	1.14760	3.72423	0.20080	0.49353
2HO9	18072	9.09615	1.14081	1.14081	3.72867	0.35190	0.35190
1XOY	17893	6.52876	0.61792	0.61792	3.43200	0.23067	0.23067

1WYO	17893	11.63480	1.29946	3.05936	5.68039	0.41667	1.22353
2OWI	17759	10.83400	1.31909	3.46062	7.15464	0.45232	1.82732
1SJR	17742	8.14458	0.98243	3.19729	3.64055	0.32489	1.32258
2JNU	17695	26.39940	2.35567	16.97410	14.44230	0.88727	9.06044
1X5B	17664	12.06230	0.99746	1.97180	6.99295	0.36640	0.93767
1PN5	17653	8.45594	1.27704	3.15145	3.74762	0.48095	1.28095
1H2O	17585	8.95301	0.98883	0.98883	4.71436	0.26736	0.26736
2D3J	17491	5.78769	0.77249	0.77249	3.77887	0.30800	0.30800
1RW2	17324	15.02420	1.68622	4.41465	7.64641	0.53859	1.91242
1JAS	17307	7.82425	1.01347	2.14787	3.82743	0.36446	0.90921
1XSC	17291	8.80727	0.93489	1.41283	4.15681	0.30207	0.53103
1WJ1	17203	13.77090	1.35931	1.35931	6.09489	0.35482	0.35482
1WFS	17155	13.80720	1.27206	2.83632	7.17333	0.45517	1.23586
1X1F	17050	12.08120	1.14539	2.10019	5.17328	0.35979	0.75529
2HF6	17024	11.81000	1.23351	3.60419	6.21639	0.38033	1.52022
1NI7	17007	7.83945	0.89291	2.45665	4.01382	0.30167	1.14335
1XN5	16981	8.09824	0.86893	1.50881	4.30224	0.23786	0.57174
2G2B	16966	7.62435	0.78337	0.78337	6.65187	0.43305	0.43305
2DKQ	16962	11.11210	1.10085	4.31890	4.18133	0.30717	1.48579
2AGM	16942	5.93641	0.68768	1.48002	2.54451	0.14837	0.48071
1TMW	16879	6.44800	1.02669	1.42900	3.31946	0.27417	0.46144
1ZGG	16787	11.71050	0.85304	0.85304	6.77830	0.39124	0.39124
1T17	16667	7.70687	0.95276	0.95276	7.05124	0.37075	0.37075
1XO8	16536	7.58758	0.73229	0.73229	4.38039	0.28497	0.28497
2AXL	16411	5.37107	0.49437	0.94309	4.61474	0.34807	0.74667
1WK1	16401	9.08896	0.97804	2.61734	4.30636	0.28803	1.11003
1P6T	16376	13.20870	1.14847	2.33712	3.95904	0.23133	0.56386
1UFG	16267	9.35455	0.96274	2.26389	4.41750	0.29923	0.91898
2GBS	16263	7.22341	0.86730	1.46981	3.27230	0.36268	0.62041
2BW2	16168	10.52190	1.13422	6.79340	5.77301	0.36477	3.55731
1JW3	16164	7.81923	0.72735	4.99701	5.11692	0.21163	2.89353
1XN6	16074	7.80131	0.87725	1.48697	3.73776	0.25211	0.52448
2BL5	16070	9.20887	0.96341	7.70844	5.57241	0.36138	4.59724
1VYN	16053	37.23550	2.61370	24.78620	31.24630	2.03992	20.91450
1WLM	15928	13.84670	1.54951	3.99002	6.70024	0.39337	1.36611
2CH0	15897	8.17515	1.16250	2.81098	3.99542	0.26575	1.08133
1WYN	15796	13.88400	1.31709	3.06228	6.75028	0.45394	1.21199
1UJO	15783	12.34230	1.19207	1.19207	6.38818	0.40602	0.40602
1TWO	15775	13.87290	1.52481	1.52481	7.38682	0.43843	0.43843
1ZTS	15746	11.78520	1.27352	8.05323	4.59987	0.26104	2.98616
2DMM	15675	10.65890	1.02622	1.02622	4.65404	0.27368	0.27368
1WPI	15656	4.97371	0.49394	0.49394	3.46544	0.14009	0.14009
2GJY	15654	4.09070	0.97637	0.97637	2.74853	0.34987	0.34987
1VDY	15562	11.81620	1.27841	1.27841	4.50098	0.23937	0.23937
1WJJ	15526	12.55640	1.20901	2.42068	4.55632	0.28846	0.64423
2DJ0	15512	9.00000	0.95077	2.24488	4.63312	0.29071	0.96855

1WIN	15455	11.46420	1.19646	1.89189	6.01236	0.38195	0.71075
2JOZ	15418	7.77984	1.20900	1.20900	3.67607	0.27881	0.27881
1U6F	15416	6.37284	0.84343	0.84343	4.36955	0.42772	0.42772
2FE0	15397	5.40188	0.58267	0.58267	3.03926	0.21350	0.21350
1GXE	15367	3.80000	0.39053	0.39053	3.15810	0.22332	0.22332
2JOE	15364	9.29881	1.47876	2.74553	3.63287	0.30484	0.75679
1NXI	15299	7.25116	1.04278	2.63702	3.47722	0.23981	1.00080
2EXN	15272	7.92288	0.85954	2.01416	4.47465	0.30156	0.93306
2ETT	15199	5.95658	0.73883	0.73883	3.74257	0.27283	0.27283
1UKX	15128	11.97210	1.24347	1.24347	6.30869	0.43073	0.43073
1XKE	15117	3.79693	0.49833	0.49833	3.26081	0.15274	0.15274
2FVT	15112	4.90577	0.51128	1.91645	2.75472	0.18082	0.98270
2DJ1	15104	9.71127	0.95866	2.47744	4.47979	0.30351	0.98873
1TQZ	15082	2.39932	0.22770	0.22770	2.22119	0.08550	0.08550
2BGO	14934	12.05240	1.05699	5.53081	5.45687	0.36209	2.36528
1PUN	14921	8.89726	1.05796	1.05796	5.91820	0.53579	0.53579
1G03	14888	5.75878	0.64687	0.64687	5.44699	0.31552	0.31552
1WLX	14812	7.22344	0.98255	0.98255	6.67808	0.48371	0.48371
1Z9B	14792	12.74630	1.51404	3.96573	5.29228	0.40723	1.27094
2JMG	14774	8.96557	0.92028	0.92028	8.40030	0.48759	0.48759
1WFM	14736	9.77742	0.99821	0.99821	3.86489	0.25923	0.25923
1WGU	14732	9.03670	0.98255	1.75120	4.69849	0.32412	0.70980
1WFI	14691	12.04830	1.36762	4.18952	4.40163	0.30497	1.18176
2I4K	14681	6.92633	0.81489	0.81489	5.03498	0.26906	0.26906
1V5M	14672	9.96253	1.17945	1.17945	4.92581	0.37258	0.37258
2JQ5	14637	8.18621	1.04845	2.64754	3.93258	0.21629	0.98034
2DJ3	14630	11.54180	1.12797	1.12797	3.69731	0.26026	0.26026
1V9W	14609	9.31619	0.86552	1.29332	5.12059	0.26840	0.52072
1IEH	14602	7.59241	1.03774	3.16023	3.71388	0.31276	1.29734
2FKI	14586	8.34305	1.06207	1.06207	4.74896	0.38940	0.38940
2DKP	14585	11.48560	1.11930	3.32327	3.61457	0.27530	0.91984
1WK0	14548	13.41370	1.27870	1.27870	4.28492	0.29749	0.29749
2B1W	14500	8.33126	0.99926	0.99926	5.48783	0.35708	0.35708
1WFJ	14491	9.06640	0.94096	0.94096	5.06075	0.29215	0.29215
1R9P	14462	5.93960	0.99689	0.99689	2.73722	0.20690	0.20690
2GM2	14450	5.33480	0.58219	0.58219	2.71617	0.15870	0.15870
1WJR	14433	10.46600	1.03423	2.58874	4.03245	0.24484	0.84366
1X5E	14387	8.24073	0.86039	1.92396	3.98229	0.26294	0.77248
2G7J	14382	12.40050	1.33508	1.99509	7.60322	0.50217	0.92251
2D9V	14377	9.79867	1.00887	1.48836	4.16324	0.28088	0.49559
1X05	14337	11.84180	1.22947	1.22947	5.68527	0.39070	0.39070
2D9Z	14321	7.24613	0.94173	2.05257	3.60911	0.30298	0.85714
1UG7	14297	12.41950	1.13046	1.96198	6.73153	0.47585	0.94602
1V5S	14256	9.76373	1.31753	3.04138	4.13874	0.28984	0.94918
1R5S	14204	10.67950	1.48858	1.48858	6.37430	0.67412	0.67412
2JP2	14195	5.08171	1.13373	1.13373	2.16423	0.29015	0.29015

1VEE	14186	7.17633	1.10236	1.10236	4.22778	0.30413	0.30413
2F1E	14176	18.55800	1.45610	10.83520	8.84639	0.45295	4.99575
1V88	14158	11.91040	1.00973	2.80451	5.47893	0.33589	1.13921
1X1G	14051	10.91020	1.14529	1.86540	5.00167	0.34114	0.68896
1WJS	14024	9.07275	0.99491	0.99491	3.98489	0.23678	0.23678
1J7H	14021	9.59697	0.94760	1.70881	4.86235	0.33940	0.74144
2DHJ	14018	9.24821	1.07701	2.50394	4.01527	0.29177	0.91773
1WFG	13993	12.75390	1.27013	2.03976	4.74631	0.28725	0.51678
2DMK	13947	7.85157	0.89800	0.89800	4.22238	0.35552	0.35552
1XHS	13934	8.84163	1.06343	7.32546	3.93397	0.38004	3.18562
1V5P	13918	9.03987	0.95576	0.95576	4.83345	0.36030	0.36030
2HGK	13847	6.30271	0.72235	2.14325	4.38027	0.26310	1.28058
1Q2Z	13846	10.96020	1.32508	4.25015	6.92251	0.47661	2.46930
1YUA	13841	14.89650	2.16092	4.34508	2.59356	0.15318	0.44386
1TQ1	13805	5.27510	0.50019	0.76148	3.66000	0.13333	0.29556
1XNE	13798	9.88874	1.30003	1.30003	3.67822	0.20957	0.20957
2GA5	13770	8.08241	0.81875	0.81875	4.16401	0.23885	0.23885
2DIY	13768	10.21860	1.01080	1.01080	4.41593	0.29351	0.29351
2GW6	13765	6.73983	0.69572	0.69572	4.50000	0.29128	0.29128
1WH4	13757	11.66220	1.15332	7.63427	4.29839	0.28871	2.62903
2JRZ	13731	8.19832	1.37146	1.37146	6.17689	0.52782	0.52782
1WFO	13725	10.62160	1.04597	1.53912	4.89187	0.26572	0.50601
2COM	13714	10.90790	1.16667	1.16667	6.01919	0.39479	0.39479
1P6Q	13713	6.62133	0.88978	0.88978	13.80280	1.03458	1.03458
1TUJ	13690	4.03202	0.70535	0.70535	3.81106	0.20255	0.20255
2DML	13661	9.29004	0.84589	3.38817	4.64855	0.28744	1.52295
1ZIT	13641	6.21587	0.55003	1.20996	3.59457	0.17014	0.57557
2D9W	13609	9.50656	1.10219	2.13002	3.85754	0.28492	0.73821
1X5I	13601	8.12833	0.82858	0.82858	3.32382	0.24799	0.24799
2DHI	13593	9.14391	1.04174	1.04174	4.38971	0.28824	0.28824
2G46	13589	5.72727	0.65593	0.65593	2.71383	0.12862	0.12862
1JFN	13573	4.10020	0.78465	0.78465	2.20347	0.19884	0.19884
1Z1M	13558	9.03458	1.14749	2.10727	2.79398	0.20139	0.52315
1WGV	13518	10.85860	1.19007	3.63467	4.29323	0.30279	1.19841
2DI7	13499	10.90540	1.36550	1.36550	3.55022	0.24000	0.24000
1TUZ	13480	8.58223	1.11951	1.11951	5.42631	0.36521	0.36521
2AVG	13469	6.65259	0.57858	1.74094	3.64055	0.16436	0.79263
2DIB	13435	8.77688	1.06121	1.06121	3.76085	0.24769	0.24769
2GQB	13433	11.49060	1.47815	4.01822	3.76208	0.33309	1.07138
1WFZ	13428	13.53840	1.23005	1.23005	6.15473	0.39770	0.39770
2HGA	13390	11.56740	1.33106	2.17432	8.28229	0.69313	1.21739
1X5K	13379	8.80955	0.90058	1.46689	3.77639	0.26297	0.50626
1UJT	13338	10.26250	1.21638	1.21638	4.20186	0.37267	0.37267
2DHK	13297	8.95317	0.99194	1.27157	3.98544	0.24757	0.34790
2D9X	13288	7.86560	0.97624	1.18855	4.20460	0.33803	0.44774
1WJO	13242	10.70490	1.08768	1.47049	4.95380	0.34919	0.49728

1X5C	13233	9.25898	0.93986	1.39307	3.85162	0.21947	0.41113
2JMX	13230	9.06774	1.21500	5.23306	4.64145	0.31564	2.26764
1UW0	13226	2.64946	0.30000	0.30000	2.89235	0.16602	0.16602
1P6S	13217	4.56402	0.56997	0.56997	3.36404	0.15790	0.15790
2DI9	13186	10.28240	1.21127	2.92530	3.66587	0.21480	0.81146
1S04	13156	9.31913	1.25347	2.41256	4.18871	0.28237	0.79752
2DJ2	13136	10.76120	1.05501	3.26070	4.90555	0.26244	1.13915
2A7O	13127	18.20680	1.57187	9.49298	10.93280	0.64485	5.35872
1V5K	13107	11.04370	1.17261	1.17261	6.57716	0.41656	0.41656
1WF5	13047	9.88011	1.00754	1.00754	4.87568	0.27027	0.27027
1V5U	13044	9.13902	1.07905	1.68563	4.22149	0.26415	0.52174
1WFT	13022	7.63372	0.92099	2.39576	3.13341	0.32611	0.96921
2HG7	13012	6.70773	1.00411	1.00411	3.16000	0.24571	0.24571
2DN6	13006	10.16600	1.07585	2.38240	4.62916	0.25198	0.86846
2DBJ	13000	9.59657	1.05188	1.36552	3.72605	0.29185	0.39749
1M3V	13000	4.50301	0.89850	0.89850	2.12528	0.14579	0.14579
1A5J	12941	10.48600	1.34375	1.34375	6.35800	0.42959	0.42959
2HJQ	12916	7.97234	1.42278	1.95897	3.75658	0.42599	0.59375
2D9Y	12905	8.07710	0.95713	1.31440	3.94920	0.28789	0.45555
2GS0	12886	10.84070	1.22166	1.22166	5.47342	0.38101	0.38101
2COD	12861	9.25162	1.21577	2.32041	3.59480	0.33369	0.74540
2JN7	12804	4.54859	0.97181	0.97181	2.64151	0.18868	0.18868
2BYE	12787	10.68370	1.06477	1.97909	3.63232	0.26230	0.60890
2DIZ	12771	8.92769	0.93418	1.62108	4.01912	0.24559	0.58971
1Y7X	12750	2.85612	0.38645	0.46730	2.55411	0.11905	0.20130
2JO6	12747	6.58031	0.98529	1.96352	2.54945	0.24490	0.59969
1VA9	12742	8.88973	1.03376	1.03376	4.20322	0.30273	0.30273
1EGX	12736	3.45313	0.31392	0.49584	3.22263	0.14471	0.33766
2GVS	12672	11.53450	0.88775	2.59049	6.39189	0.34595	1.32432
1XDX	12666	2.57269	0.21107	0.37048	4.37710	0.26263	0.56341
1L4S	12653	10.44830	1.43890	1.43890	5.18931	0.44543	0.44543
1LL8	12640	9.96164	1.21039	2.91988	4.46946	0.32524	1.06255
1WFN	12624	8.62589	0.81968	0.81968	3.41694	0.20266	0.20266
2DA0	12617	9.53706	1.14967	1.42880	4.07018	0.29298	0.39474
2H7A	12563	6.41654	0.51736	3.05946	4.56275	0.16802	2.05061
1ZG2	12539	9.89394	1.17607	1.86834	4.25346	0.24494	0.63046
1RDU	12533	7.68865	0.72634	0.72634	3.26790	0.10137	0.10137
1Z7P	12517	10.59640	0.94497	1.64295	4.75000	0.27151	0.61559
2JQO	12507	8.06032	0.80696	0.80696	3.14113	0.18875	0.18875
1WQU	12490	9.02770	0.90198	2.38342	3.79733	0.24000	0.81600
1V5Q	12475	8.46793	0.85139	1.64706	4.44708	0.27920	0.72080
1TVM	12464	4.87031	0.51641	0.51641	2.82781	0.07914	0.07914
1TI3	12442	7.34241	0.58245	1.23500	3.89715	0.14692	0.53627
1V9V	12410	13.17120	0.92838	0.92838	7.35750	0.38111	0.38111
1WI0	12392	11.13030	0.89080	1.85428	5.18065	0.27903	0.72581
1KVN	12390	11.51410	1.87661	4.75466	3.46962	0.25844	1.08390

2DS4	12371	9.89108	1.10762	1.10762	4.36309	0.25870	0.25870
1UEM	12354	7.95318	0.85022	5.08556	3.34252	0.24426	2.00735
1RJJ	12346	6.32922	0.86004	0.86004	3.07679	0.12321	0.12321
2DMB	12335	7.53172	0.80617	3.20776	3.01018	0.09572	0.98167
2JMP	12327	4.96503	0.47345	1.27137	3.24570	0.13145	0.71588
1UC6	12318	7.09109	0.63639	2.06481	3.82200	0.16400	0.93400
1UJX	12315	11.22110	0.96488	11.22110	4.41907	0.24580	4.41907
1X6D	12306	9.72837	0.82597	5.02157	4.42790	0.24922	2.08934
2AJE	12305	8.15103	1.11670	3.40046	11.45420	0.49318	3.70370
1KKG	12274	7.13057	1.15597	1.15597	5.30168	0.35056	0.35056
1UHT	12244	10.64320	0.85233	0.85233	4.48669	0.26464	0.26464
2H0P	12222	7.05373	0.80621	1.59562	3.65909	0.21364	0.59091
1KVZ	12222	9.04541	1.08408	2.82685	3.49117	0.21990	0.88283
2JN9	12221	7.08621	0.91379	0.91379	3.06435	0.18795	0.18795
2F05	12221	6.13964	0.53025	0.53025	5.41222	0.28516	0.28516
1Z2K	12195	5.81951	0.47998	3.71912	3.38943	0.09924	1.93312
1S6N	12186	6.52848	0.93585	1.87985	4.12730	0.30318	0.93635
2APN	12184	9.65503	0.95221	0.95221	6.54479	0.38378	0.38378
1TTX	12170	8.49915	1.09149	2.23274	4.54811	0.32795	0.98537
2GMG	12151	7.55014	1.13534	1.13534	5.27849	0.34093	0.34093
1T0G	12151	6.51173	0.59230	1.54600	3.68278	0.13401	0.71756
1RQ8	12146	14.22490	1.04630	3.26606	5.44508	0.36127	1.19609
1J26	12139	11.48580	1.14018	1.14018	5.68807	0.32694	0.32694
1IYR	12127	4.17588	0.14573	3.56784	5.83804	0.36912	5.21281
2HGC	12091	12.64330	1.15170	8.07897	7.85179	0.51439	4.87533
1WH8	12085	11.90720	0.94974	2.15262	5.82762	0.35356	0.95690
1YHD	12076	7.86930	0.57608	1.29667	4.66165	0.20719	0.68338
1RJV	12054	13.58600	0.69690	0.69690	5.98383	0.17610	0.17610
1X9A	12038	7.57425	0.67485	2.33114	4.05882	0.14199	0.96349
1WGQ	12004	8.68168	0.91365	2.36989	4.27832	0.24919	0.92557
2DMC	12001	9.44267	0.94196	2.05420	4.26137	0.23814	0.64666
2COC	11995	8.86779	0.72367	0.72367	4.46937	0.21743	0.21743
2JNG	11992	67.52920	4.09934	32.95700	38.67520	2.00355	18.68060
1JJG	11984	7.58045	1.17232	2.04567	2.15385	0.11469	0.36923
1JH3	11983	5.62246	0.88972	1.47293	3.93886	0.30393	0.74411
1WJP	11968	9.91217	1.03074	2.79622	5.76373	0.30283	1.25125
1P68	11925	16.70150	1.36498	1.36498	9.11607	0.51531	0.51531
1WYL	11919	10.74120	0.80927	3.08221	6.20717	0.28816	1.62773
2E0G	11915	9.25115	1.09373	1.09373	5.11354	0.34315	0.34315
1UEW	11901	8.75268	0.93200	0.93200	3.75337	0.22383	0.22383
1TE7	11899	6.18410	0.59167	0.59167	3.67506	0.16734	0.16734
1YH5	11888	8.82653	0.90051	6.03265	3.66854	0.21161	2.22285
1IE5	11830	8.56749	0.99557	1.75862	3.95337	0.26079	0.54577
1WIK	11825	12.17430	1.12079	1.12079	4.38483	0.20095	0.20095
2E6J	11820	8.03736	0.78151	6.64779	3.86408	0.22242	3.09797
2FEB	11799	6.82066	0.91098	0.91098	2.39477	0.18312	0.18312

2DJS	11795	8.87549	0.85195	0.85195	3.69231	0.18974	0.18974
1R6R	11789	32.66820	2.29173	17.35040	25.73320	1.56024	13.39620
2COF	11771	8.26790	0.72673	1.13094	3.37696	0.14931	0.28387
2D7M	11735	9.18366	0.87024	3.50120	4.71217	0.23591	1.63501
1SJQ	11733	10.62900	1.05945	6.97459	6.85061	0.48163	4.14531
2F09	11721	6.64348	0.65894	0.65894	2.71494	0.04598	0.04598
1YYJ	11708	11.15690	1.18076	1.18076	9.13827	0.56914	0.56914
1VAE	11697	9.34304	0.91860	1.50629	4.19466	0.17366	0.40458
1M7T	11678	10.79100	0.57525	1.04473	4.29065	0.21151	0.40863
1XN9	11674	12.26740	1.07469	1.07469	4.25923	0.23136	0.23136
2DN7	11667	10.75690	0.99492	1.65106	3.69737	0.21711	0.45724
2D7P	11656	8.31970	0.89542	1.39881	3.89889	0.29135	0.46615
2DJM	11654	3.74452	0.45853	0.63966	2.88964	0.19545	0.33024
1L1P	11638	3.67500	0.45217	0.67717	3.18791	0.11520	0.32295
2H5M	11609	6.44940	0.62619	1.28312	3.47113	0.19582	0.50377
1V63	11598	14.03660	1.29165	5.82348	8.76947	0.42958	3.35327
1WJQ	11571	8.99804	0.91356	2.42891	3.93743	0.22793	0.73073
1WF1	11563	10.52660	1.03431	2.37693	4.05500	0.19833	0.59333
2DB8	11538	7.69185	0.80642	1.33383	3.56639	0.19710	0.46058
2DI8	11522	8.91828	0.96591	3.06078	4.01550	0.18949	1.10594
1UL7	11521	8.15367	0.95713	2.34903	3.45780	0.22840	0.86197
2G1D	11519	10.89680	1.25920	1.25920	4.74238	0.33426	0.33426
1WEY	11515	7.83053	0.99051	1.63476	3.53529	0.19978	0.50163
1WF9	11512	9.85684	0.96554	1.93071	4.96974	0.30836	0.69885
1SJ6	11501	6.88390	0.47833	2.52865	4.40393	0.16086	1.44236
2JNE	11477	5.46777	0.90180	0.90180	2.48466	0.19632	0.19632
1T4Z	11475	9.98086	0.94215	3.25308	6.30248	0.25785	1.63636
2OA4	11463	8.20304	1.13518	2.74104	5.10643	0.29505	1.35511
2IDA	11414	7.14772	0.80288	0.80288	3.53455	0.12727	0.12727
2DA6	11404	9.55669	0.96438	1.26929	6.78915	0.40465	0.64961
1V32	11400	10.92240	0.97323	3.53228	4.93991	0.17579	1.28430
2GZZ	11385	8.81172	0.55893	2.57774	4.44411	0.18629	1.20119
2JNA	11373	8.10485	0.89039	0.89039	3.60969	0.26496	0.26496
1SS6	11350	6.02134	0.57556	0.57556	3.90486	0.27156	0.27156
1XSF	11345	5.40846	0.57858	0.57858	4.22718	0.16864	0.16864
1S62	11316	7.49557	0.60755	0.60755	3.79634	0.10526	0.10526
1UJU	11305	10.82490	0.91091	0.91091	4.76560	0.25419	0.25419
1Q7X	11283	10.48040	1.08778	1.08778	7.74379	0.57616	0.57616
1WJK	11247	10.47540	0.95036	2.55319	4.47100	0.26538	0.93849
2D7O	11246	8.14234	0.95134	3.44180	3.01336	0.14248	1.05254
1WGY	11244	10.18120	1.05750	2.13049	4.72749	0.24645	0.75592
1WJT	11232	17.03020	1.30344	3.12717	6.39202	0.32958	0.93322
2I9H	11231	11.73440	0.92367	2.67058	4.15201	0.26379	0.85097
1WG5	11210	8.96031	0.89423	0.89423	4.39746	0.20327	0.20327
1JDQ	11203	2.88825	0.39884	0.52216	2.78041	0.08108	0.23987
2DIA	11195	9.80835	0.79557	6.29003	3.32500	0.12692	1.91731

2DJ4	11185	10.45550	0.90481	2.38332	4.35224	0.22985	0.83433
1WFV	11175	11.07450	0.95843	1.34646	4.60979	0.24329	0.40758
1SG7	11169	8.77416	0.69466	3.95925	5.59159	0.20821	2.41041
2PPH	11167	6.87943	1.10172	3.83230	3.63093	0.32990	1.72165
1YZC	11157	16.41880	1.51963	2.52181	6.98396	0.57143	0.95493
1WFY	11155	12.27140	0.96912	0.96912	5.27175	0.35446	0.35446
1NY8	11101	7.18462	0.92208	1.52457	6.19447	0.35876	0.91199
1WH6	11067	11.11630	1.04673	3.51880	6.16273	0.39720	1.77953
1J0F	11046	8.99462	0.78271	1.06035	4.53077	0.23692	0.33077
2GTO	11044	5.99056	0.70905	2.14675	3.01575	0.17060	0.95276
2CO9	11044	10.56510	1.12106	1.12106	6.69430	0.36477	0.36477
2JN6	11030	9.20970	1.17184	2.93937	6.54133	0.43597	1.66757
1WJU	11027	12.94990	1.04762	1.49536	4.20482	0.18474	0.29920
2NOC	10987	8.45343	1.12134	1.12134	3.26237	0.15892	0.15892
1AKP	10961	4.50172	0.66708	0.66708	2.53734	0.11840	0.11840
2CUM	10948	8.75668	0.85853	1.42765	3.68618	0.25761	0.50586
1UEP	10938	8.41306	0.95437	3.49712	4.01149	0.25058	1.42529
2UZG	10914	7.30640	0.58557	1.59056	4.08104	0.17656	0.69754
2DJU	10899	8.16230	0.78064	1.41581	3.55901	0.16977	0.43064
1XS8	10898	6.03668	0.57177	3.89234	4.88958	0.24167	3.03958
2JOY	10865	11.24450	1.35484	1.35484	3.82396	0.18093	0.18093
2D7Q	10849	8.12035	0.82548	4.24731	3.39705	0.15812	1.55864
1UFX	10848	10.49820	0.96093	1.37898	4.29529	0.25340	0.37068
1VJ6	10843	7.23669	0.96217	0.96217	3.82722	0.25089	0.25089
2DKM	10840	9.12268	0.83089	0.83089	2.96259	0.12718	0.12718
1Z3R	10833	6.97033	0.84067	1.84689	2.81128	0.23152	0.58755
2HWT	10828	8.96647	1.08699	1.08699	3.18268	0.21964	0.21964
1LWM	10810	5.93633	1.08171	1.55854	3.79475	0.27594	0.68187
1WEZ	10806	8.41357	0.81106	0.81106	3.25084	0.17391	0.17391
1JE3	10804	3.04401	0.37026	0.46131	2.22759	0.06207	0.15172
1SXL	10777	5.82003	0.99609	2.09781	2.22590	0.22590	0.65840
2DIC	10697	8.93161	0.86281	5.76528	4.20993	0.27665	2.58747
2NPB	10696	9.10215	1.28045	3.51605	7.87107	0.60496	2.50083
1WJN	10691	10.87600	0.98176	2.27032	4.98282	0.30916	0.79771
2FB7	10685	13.80640	1.31809	2.73298	4.94340	0.21462	0.71934
2JOQ	10661	9.04403	0.99790	1.84253	3.64789	0.20322	0.46881
2CUF	10648	7.77967	0.76897	1.42850	4.54227	0.30321	0.74830
1V5R	10642	8.65373	0.96220	1.54435	3.32537	0.16202	0.37981
1N27	10636	9.98911	1.22775	3.31250	2.98025	0.18025	0.71605
1WGR	10612	7.82922	0.81209	1.00151	4.24287	0.16473	0.30201
1WGW	10604	12.45780	1.08439	3.08204	6.37624	0.27393	1.29373
2DJT	10527	9.33250	0.88517	1.40589	4.15136	0.30273	0.50051
2JM3	10515	9.22425	0.94863	2.96016	3.17457	0.19422	0.85318
1WJZ	10442	11.94610	1.10951	2.44083	5.23141	0.28063	0.85445
1ZFS	10418	9.30814	1.47516	1.47516	6.97436	0.47436	0.47436
1WHA	10399	9.12119	0.86100	2.76005	3.85499	0.14878	0.80038

2DLG	10370	7.71030	0.81332	1.15569	3.29263	0.18433	0.33410
2G0U	10354	9.13019	1.02875	2.08466	6.41055	0.40826	1.08945
2HLU	10319	9.20681	0.91309	4.95340	4.01405	0.23178	1.80685
1WF2	10279	8.86454	0.86500	0.86500	3.41131	0.16874	0.16874
2IZ4	10267	4.53975	0.25523	0.45188	6.34623	0.26069	0.61507
1UJS	10232	9.46186	0.86190	0.86190	4.31711	0.23548	0.23548
1J8K	10203	6.86358	0.87412	1.26932	2.82835	0.26578	0.42746
2HDM	10189	42.67910	3.78899	8.39835	17.30850	1.08958	2.97328
2H45	10187	7.79178	0.80150	1.52608	4.67977	0.25225	0.58641
2E29	10135	6.83840	0.69696	0.69696	3.89550	0.19927	0.19927
1B64	10131	8.12694	0.83834	0.83834	3.67319	0.21526	0.21526
1V5N	10108	8.52499	0.97200	0.97200	3.99560	0.24176	0.24176
1JNS	10106	6.24338	0.96644	1.77517	2.83864	0.19322	0.59610
1V31	10097	11.40520	0.93472	2.84672	3.59828	0.20838	0.77766
2HJJ	10082	32.17930	1.86363	32.17930	15.40700	0.61709	15.40700
1RRZ	10057	69.57790	2.86697	54.91500	20.52370	0.76274	16.23200
1L7B	10016	5.70115	1.11902	1.25399	4.22321	0.28571	0.40402
1D5G	10008	3.39890	0.44355	0.65028	3.53580	0.11547	0.39492
2BBX	5440	5.30836	0.70293	1.63058	2.85433	0.09449	0.62598

* Molecular weight is specified in Dalton
† CSSO index obtained at number of points where the calculated protein structures were found at the minimum RMSD value from the corresponding PDB reference structures
